# The NF-κB Isoform p65 iso5 Is Associated with Distinct Transcriptional Programs and Signaling Pathways

**DOI:** 10.3390/cells15181643

**Published:** 2026-09-10

**Authors:** Gaetano Spinelli, Ilaria Cosentini, Giuseppa Biddeci, Judit Mihaly-Bison, Gioacchin Iannolo, Giovanni Duro, Carmela Zizzo, Paolo Colomba, Johannes A. Schmid, Francesco Di Blasi

**Affiliations:** 1 Institute for Biomedical Research and Innovation, National Research Council of Italy (IRIB-CNR), Via Ugo La Malfa 153, 90146 Palermo, Italy; gaetano.spinelli@irib.cnr.it (G.S.); ilaria.cosentini@irib.cnr.it (I.C.); giuseppa.biddeci@irib.cnr.it (G.B.); giovanni.duro@irib.cnr.it (G.D.); carmela.zizzo@irib.cnr.it (C.Z.); paolo.colomba@irib.cnr.it (P.C.); 2 Center for Physiology and Pharmacology, Institute of Vascular Biology and Thrombosis Research, Medical University of Vienna, Schwarzspanierstraße 17, 1090 Vienna, Austria; judit.mihaly-bison@meduniwien.ac.at; 3 Department of Research, IRCCS ISMETT (Istituto Mediterraneo per i Trapianti e Terapie ad alta Specializzazione), Via E. Tricomi 5, 90127 Palermo, Italy; gioacchin.iannolo@unikore.it; 4 Department of Medicine and Surgery, Università degli Studi di Enna “Kore”, 94100 Enna, Italy

**Keywords:** NF-κB, RelA, p65 iso5, dexamethasone, RNA-seq, inflammation disease

## Abstract

**Highlights:**

**What are the main findings?**

- p65 iso5 drives transcriptional programs and signaling pathways distinct from canonical p65.
- p65 iso5 binds dexamethasone and exhibits an isoform-specific transcriptional response to glucocorticoid treatment.

**What are the implications of the main findings?**

- p65 iso5 may selectively modulate inflammatory signaling pathways through mechanisms distinct from canonical NF-κB signaling.
- NF-κB isoform diversity may contribute to context-dependent regulation of inflammation and glucocorticoid responses.

**Abstract:**

NF-κB p65 (RelA) is a key regulator of inflammation, immunity, and stress responses. Recent evidence indicates that alternative p65 isoforms may diversify NF-κB signaling, but their functions remain largely unexplored. We previously identified a novel splice variant, p65 iso5, which contains an additional upstream exon and displays distinct molecular properties, including the ability to interact with dexamethasone in a glucocorticoid receptor-dependent manner. Here, we define the transcriptional programs regulated by p65 iso5 using RNA-seq analysis of HeLa cells expressing either p65 iso5 or canonical p65, with or without dexamethasone treatment. Under basal conditions, p65 iso5 expression was associated with reduced expression of genes involved in type I interferon and antiviral pathways as compared to the effects of canonical p65, while genes involved in translation, ribosome biogenesis, and metabolic activity were not reduced as with p65. Upon dexamethasone stimulation, p65 iso5 expression was associated with extensive transcriptome remodeling characterized by suppression of biosynthetic and proliferative programs and activation of metabolic and stress-adaptive pathways. Comparative analyses reveal that glucocorticoid responses are strongly isoform-dependent, with p65 iso5 expression being associated with distinct gene expression networks supported by distinct protein–protein interaction hubs. p65 iso5 is overexpressed in cirrhotic and hepatocellular carcinoma tissues as well as in high-grade colon tumors, suggesting clinical relevance. Our findings identify p65 iso5 as a context-dependent NF-κB modulator with unique transcriptional and metabolic functions and potential relevance in inflammation-associated diseases and cancer.

## 1. Introduction

NF-κB is a key transcription factor that plays a central role in regulating immune and inflammatory responses, while also supporting cell survival, growth, and adaptation to stress [1,2]. The mammalian NF-κB family of transcription factors is composed of five members: NF-κB1 (p105/p50), NF-κB2 (p100/p52), p65 (RELA), RELB (v-Rel reticuloendotheliosis viral oncogene homolog B), and c-REL, which associate with each other to form distinct transcriptionally active homo- and heterodimeric complexes [3,4]. Two major NF-κB signaling pathways have been described: the canonical pathway, which is mainly activated by pathogens and inflammatory mediators, and the noncanonical pathway mostly activated by developmental signals [5]. The most common NF-κB complex that is activated by pathological stimuli through the canonical pathway is the p65/p50 heterodimer [6], with the p65 (RelA) subunit being the most extensively studied and recognized as a central mediator of canonical NF-κB signaling [1]. p65 controls the transcription of numerous genes involved in immune defense, cytokine release, programmed cell death, and cell cycle progression [6,7,8]. Dysregulation of p65 activity has been linked to a variety of pathological conditions, including autoimmune and chronic inflammatory diseases as well as cancer, highlighting its essential role in maintaining cellular homeostasis [9,10,11]. Recent studies suggest that NF-κB activity is modulated not only by its canonical expression variants but also through the presence of alternative isoforms that can influence cell type-specific gene expression and adapt responses to different contexts [12,13]. While p65 is well recognized as a key regulator of inflammation and immune responses, the diversity and functional significance of its isoforms remain poorly characterized. Alternative splicing of the RelA gene can produce p65 variants with distinct structural and functional properties, potentially influencing target gene specificity, transcriptional regulation, and signaling cross-talk [12]. We identified a new p65 isoform, named p65 iso5, which contains an additional exon upstream of the first canonical exon and exhibits distinctive molecular characteristics. Furthermore, the overexpression of p65 iso5 has been observed in human tissues, including peripheral blood mononuclear cells (PBMCs) from patients with inflammatory conditions and liver tissues from patients with cirrhosis and hepatocellular carcinoma (HCC) [12]. Among the principal transcriptional regulators of inflammation, NF-κB and the glucocorticoid receptor (GR) play antagonistic roles in controlling inflammatory signaling [14,15]. NF-κB promotes the expression of proinflammatory cytokines, adhesion molecules, and immune mediators, while GR activation by glucocorticoids suppresses these pathways to restore immune homeostasis [1,16,17,18]. This antagonistic relationship occurs through multiple mechanisms, including mutual inhibition of DNA binding, competition for coactivators, and reciprocal modulation of transcriptional activity [19,20]. Crosstalk between NF-κB and GR is therefore essential for maintaining a balanced inflammatory response and preventing tissue damage caused by excessive immune activation [21]. In this context, the identification of p65 iso5 as a GR-interacting isoform capable of interacting with dexamethasone and enhancing its anti-inflammatory functions on GRE elements introduces a new layer of complexity in this regulatory network. Despite these emerging insights, the regulatory networks and functional roles of p65 iso5 remain largely uncharacterized, leaving open questions regarding how this isoform contributes to the fine-tuning of NF-κB-dependent gene expression and inflammatory responses. Investigating this aspect is particularly important for understanding the molecular mechanisms underlying immune regulation and for identifying potential therapeutic targets in inflammation- and immunity-related diseases. In the present study, we report on the analysis of transcriptional effects of p65 iso5 as compared to the canonical isoform using RNA sequencing (RNA-seq). We examine the transcriptome of HeLa cells overexpressing either p65 iso5 or canonical p65, in the presence or absence of dexamethasone, and investigate its context-specific effects on NF-κB signaling. By defining the transcriptional and metabolic programs uniquely regulated by p65 iso5, our work expands the understanding of p65 isoform diversity and reveals previously unrecognized mechanisms that coordinate immune, metabolic, and stress-adaptive responses. These results emphasize the context-dependent activities of p65 iso5 and provide a basis for further studies on explorations of its possible contribution to disease mechanisms and adaptive cellular responses.

## 2. Materials and Methods

### 2.1. Human Liver and Colon Samples

The study on human liver and colon samples was conducted after obtaining IRCCS-ISMETT approval (Istituto Mediterraneo per i Trapianti e Terapie ad alta specializzazione—Department of Laboratory Medicine and Advanced Biotechnologies), and patients provided informed consent for research studies. We confirm that the human samples were used in accordance with national guidelines and regulations. This study was conducted in accordance with the Declaration of Helsinki.

### 2.2. Cell Cultures and Transfections

HeLa cells (ATCC^®^ CCL-2™) were cultured at 37 °C in a humidified atmosphere containing 5% CO_2_ in RPMI 1640 medium (Gibco, Thermo Fisher Scientific, Waltham, MA, USA) supplemented with 10% heat-inactivated fetal bovine serum (FBS; Gibco) and 1X Penicillin–Streptomycin (Gibco). For RNA-seq experiments, transfections were performed using Lipofectamine™ P3000 (Invitrogen, Thermo Fisher Scientific) according to the manufacturer’s instructions. Cells were seeded in 25 cm^2^ culture flasks and transfected 24 h later with the appropriate concentrations of the specified GFP plasmid. To maintain a constant total DNA amount in each sample, the appropriate quantity of the corresponding empty plasmid was added. All experiments were performed in triplicate and independently repeated at least twice.

### 2.3. Cell Sorting and Dex Treatment

HeLa cells were harvested and resuspended in sterile phosphate-buffered saline (PBS) supplemented with 2% fetal bovine serum (FBS; Gibco, Thermo Fisher Scientific, Waltham, MA, USA) and 1 mM ethylenediaminetetraacetic acid (EDTA). Cell sorting was performed using an S3e Cell Sorter (Bio-Rad, Hercules, CA, USA) based on GFP fluorescence intensity. Prior to sorting, cell debris and doublets were excluded by gating on Forward Scatter (FSC) and Side Scatter (SSC) parameters. Within the single-cell population, GFP-positive (GFP^+^) cells expressing either GFP-p65 or GFP-p65 iso5 fusion proteins or GFP alone as a control, were identified based on fluorescence intensity measured on the FITC channel. GFP-positive cells were sorted within a comparable moderate fluorescence intensity range for the different GFP-tagged constructs. This strategy was adopted to prevent effects of strong overexpression and to minimize variability associated with differences in transfection efficiency and, furthermore, to obtain experimentally matched cell populations for the subsequent transcriptomic analyses. Since fusion proteins of GFP with either p65 or p65 iso5 were used, comparable GFP fluorescence also means similar protein levels. However, this does not exclude potential differences in stability, subcellular localization, or functional activity of p65-GFP and p65 iso5-GFP fusion proteins. After seeding, cells were treated with 5 × 10^−7^ M dexamethasone (Dex) for 24 h or left untreated. At the end of the treatment, cells were harvested by trypsinization, and total RNA was isolated for subsequent analyses.

### 2.4. RNA Isolation and RNA Sequencing

Total RNA was isolated using the PeqGold Total RNA Kit (VWR International, Vienna, Austria) according to the manufacturer’s instructions. RNA concentration and purity were assessed using a NanoDrop spectrophotometer (Thermo Fisher Scientific, Waltham, MA, USA), and only samples with an A_260_/A_280_ ratio greater than 1.8 were used for downstream analyses. Aliquots of purified RNA were sent to the Research Center for Molecular Medicine of the Austrian Academy of Sciences (CeMM, Vienna, Austria) for RNA sequencing.

### 2.5. Quality Control and Processing

Raw sequencing reads were assessed for quality using MultiQC (v.1.31) [22]. Library adapter sequences and low-quality bases (Phred score < 20) were removed using TrimGalore (https://zenodo.org/badge/latestdoi/62039322, accessed on 6 September 2026). The overall quality of filtered reads was re-evaluated to ensure data integrity, and the clean reads were retained for downstream analysis. Reads were aligned to the reference human genome (Homo sapiens-GRCh38) using HISAT2 (v.2.2.1) [23] with default parameters. The resulting alignment files (SAM) were converted into BAM files, sorted by index using SAMtools (v.1.17). Gene-level read counts were quantified using feaureCounts (v.2.0.1) [24] based on gencode annotation file (gtf version 48). Raw counts were normalized to library size and transformed using the variance stabilizing transformation (vst) function of DESeq2 (v1.42.0) [25]. Genes with fewer than 10 total reads across all samples were excluded.

RNA-sequencing libraries were prepared with the NEBNext^®^ Poly(A) mRNA Magnetic Isolation Module E7490 and the NEBNext^®^ Ultra™ II Directional RNA sample preparation kit E7760 (New England Biolabs, Inc., Ipswich, MA, USA). NGS library concentrations were quantified with the Qubit 2.0 assay, and the size distribution was assessed using the 2100 Bioanalyzer instrument (Agilent Technologies, Santa Clara, CA, USA). Before sequencing, sample-specific NGS libraries were diluted and pooled in equimolar amounts. Expression profiling libraries were sequenced on a NovaSeq 6000 instrument (Illumina, San Diego, CA, USA) following a 100-base-pair, paired-end recipe. An average of 37 × 106 raw sequencing reads were obtained in BAM format and converted to FASTQ files using samtools fastq (v1.5). Initial quality assessment of raw FASTQ files was performed with FastQC (v0.12.1). Reads were trimmed for quality and adapter sequences using Trim Galore v0.6.10 [Available online: https://www.trimgalore.com/guide/overview/ (accessed on 7 April 2025)] with a Phred score cutoff of 30 and a minimum read length of 30 bp. Reads (~36 × 106 per sample) were aligned to the human genome GRCh38 release 95 primary assembly using STAR (v2.7.11b) [26], with genome indices and transcript annotations based on Ensembl release 95 in a two-pass mapping, reporting only reads that mapped exactly once to the reference genome (-outFilterMultimapNmax 1), with gene counts performed during alignment using the option––quantMode GeneCounts. Further post-alignment quality control was done with Qualimap (v2.2.2a, qualimap bamqc, qualimap rnaseq). Quality control results were summarized with MultiQC (v1.0.dev0).

### 2.6. RNA-Seq Experimental Design and Differential Expression Analysis

The RNA-seq experiments included three independent biological replicates for each experimental condition. Raw gene-level counts were analyzed using DESeq2 (v1.42.0) [25]. Genes with fewer than 10 total reads across all samples were excluded from the analysis. Normalized expression values were generated using the variance stabilizing transformation (VST) function of DESeq2 for visualization and exploratory analyses. Differential expression was assessed using the DESeq2 Wald test, and *p*-values were corrected for multiple testing using the Benjamini–Hochberg procedure [27]. Genes with an adjusted *p*-value < 0.05 and an absolute log2 fold change ≥1 were considered differentially expressed. The differential expression analyses were performed using the following comparisons: (i) p65 iso5 versus canonical p65 under basal conditions; (ii) p65 iso5-Dex versus p65-Dex; (iii) p65 iso5-Dex versus p65 iso5; and (iv) p65 iso5-Dex versus GFP-Dex. The DESeq2 model was specified as, with the corresponding contrasts defined as. The number of biological replicates and the experimental comparison definitions were kept consistent across the transcriptomic analyses. The complete list of differentially expressed genes, including gene identifiers, fold changes, adjusted *p*-values, and comparison definitions, is provided in the Supplementary Information.

### 2.7. DEG Identification

Differentially expressed genes were identified using DEseq2 and the Wald test [25]. *p*-values were corrected for multiple tests to obtain the false discovery rate (FDR) using the Benjamini & Hochberg method [27]. Genes with an adjusted *p*-value < 0.05 and absolute log2FC ≥ 1 were considered differentially expressed genes (DEGs). DEGs identification was performed for different comparisons:

- p65 vs. GFP control
- p65 iso5 vs. GFP control
- p65 iso5 vs. p65;
- p65 iso5-Dex vs. p65 -Dex
- p65 iso5-Dex vs. p65 iso5

Annotation was performed with packages biomaRt version (v2.58.0) [28,29] and org.Hs.eg.db (v3.18.0) (https://doi.org/doi:10.18129/B9.bioc.org.Hs.eg.db). The R packages ggplot2 (v3.4.4) (Hadley Wickham. 2016. Ggplot2: Elegant Graphics for Data Analysis (2nd ed.). Springer Publishing Company, Incorporated.) and pheatmap (v1.0.12) were used for data visualization. Differential expression analyses were performed using predefined pairwise comparisons between the experimental conditions. UpSet and Venn diagram analyses were subsequently used to summarize the overlap and specificity of differentially expressed genes across these comparisons. Up- and downregulated genes were further compared using GraphPad Prism 11 to create scattergrams showing the number of regulated genes and their expression changes. These analyses were used to descriptively characterize differences in Dex-responsive transcriptional profiles between p65 and p65 iso5 conditions.

### 2.8. GSEA Analysis

To identify functionally enriched biological pathways, Gene Set Enrichment Analysis (GSEA) was performed for the different comparisons using the ClusterProfiler (v.4.10.0) R package [30]. All the expressed genes were ranked according to the statistical value (Wald test statistics) obtained from DESeq2, which reflects the direction and magnitude of differential expression. Enrichment analysis was performed using the Gene Ontology (GO) domain “Biological Process” retrieved from the org.Hs.eg.db annotation package. The analysis includes terms with more than 10 and fewer than 500 genes per term and 10,000 permutations as the GSEA default. Pathways with an adjusted *p*-value (Benjamini & Hochberg method) were considered significantly enriched. The Normalized enrichment score (NES) was used to assess the strength and direction of enrichment of each GO term. For each comparison, GSEA enrichment plots were generated for the pathways with the highest and lowest NES values, representing the most positively and negatively enriched biological process, respectively.

### 2.9. Identification of DEGs That Are Unique in Pairwise Comparisons of Conditions

To identify condition-specific DEGs, the DEG lists from the five different comparisons were interpolated. Specifically, genes uniquely regulated in one comparison, and not in others, were defined as unique DEGs for that specific condition. Excluding commonly regulated genes in the comparisons p65 iso5-Dex vs. Dex and p65 iso5-Dex vs. GFP-Dex, we removed any transfection-related effects, as well as the effects of Dex treatment alone and GFP expression. In addition, selecting unique DEGs included in the remaining comparisons allowed us to identify genes specifically altered by overexpression of p65 iso5 (p65 iso5 vs. p65), by treatment with Dex (p65 iso5-Dex vs. p65 iso5), and both conditions (p65 iso5-Dex vs. p65-Dex). The results are reported as an UpSet plot [31] and a Venn diagram [32].

### 2.10. PPI Network Analysis

Protein–protein interaction (PPI) network analysis was performed to explore the functional relationships among the unique DEGs. The list of significant DEGs was uploaded to the NetworkAnalyst (v3.0) web platform [33,34,35,36,37] for network construction and visualization. Interactions were retrieved from the STRING database (Search Tool for the Retrieval of Interacting Genes/Proteins) with a confidence score cutoff of 700, considering only experimentally validated interactions; minimum- or first-order networks were computed. The networks were downloaded as graphml files and imported into the Cytoscape software (v.3.10.3). The STRING app was then used for functional enrichment analysis.

### 2.11. Real-Time Quantitative PCR (qPCR)

Oligonucleotide primers for qPCR were designed based on gene sequences obtained from the NCBI GenBank database and validated for the absence of secondary structures, self-dimers, and for optimal amplification efficiency and specificity. Each qPCR reaction was performed in a total volume of 20 μL containing 10 μL of ExcelTaq™ 2× Fast Q-PCR Master Mix (SYBR, ROX) (SMOBIO, Hsinchu City, Taiwan), 10 pmol/μL of each forward and reverse primer (Table 1), 50 ng of cDNA, and RNase-free H_2_O (Gibco, Thermo Fisher Scientific, Waltham, MA, USA). The reactions were carried out in 96-well PCR plates using a StepOnePlus™ Real-Time PCR System (Applied Biosystems, Thermo Fisher Scientific, Waltham, MA, USA) covered with MicroAmp™ Optical Adhesive Film (Applied Biosystems). The thermal cycling program consisted of 40 cycles of 95 °C for 20 s, 95 °C for 3 s, and 60 °C for 30 s. All cDNA samples were analyzed in triplicate, and housekeeping genes were amplified on the same plate to minimize inter-sample variation. No-template and no-reverse-transcription controls were included in each experiment. All experiments were performed in triplicate and independently replicated twice.

### 2.12. Microscopic Image Acquisition and FRET

Microscopy was performed on a Nikon A1 R+ laser scanning confocal system equipped with 12-bit detectors using a 60× plan apochromatic oil immersion objective (NA 1.4). Live cell microscopy was carried out under physiological conditions (37 °C and 5% CO_2_) on a computer-controlled Olympus IX71 system with LED excitation, an emission filter wheel, and an Andor EMCCD iXon Life camera (Andor Technology Ltd., Belfast, Northern Ireland, UK). Time-lapse images were collected at 1-min intervals. The donor channel was acquired with excitation at 488 nm and a 525/50 emission filter. The FRET channel was acquired with excitation at 488 nm and a 595/50 emission filter. The acceptor channel was acquired with excitation at 561 nm and a 595/50 emission filter. The FRET value calculation was conducted using the Fiji software package of ImageJ v1.54p (National Institutes of Health, Bethesda, MD, USA) as described in [38].

## 3. Results

### 3.1. Overexpression of p65 iso5 in Liver and Colon Cancer

**Figure 1:**
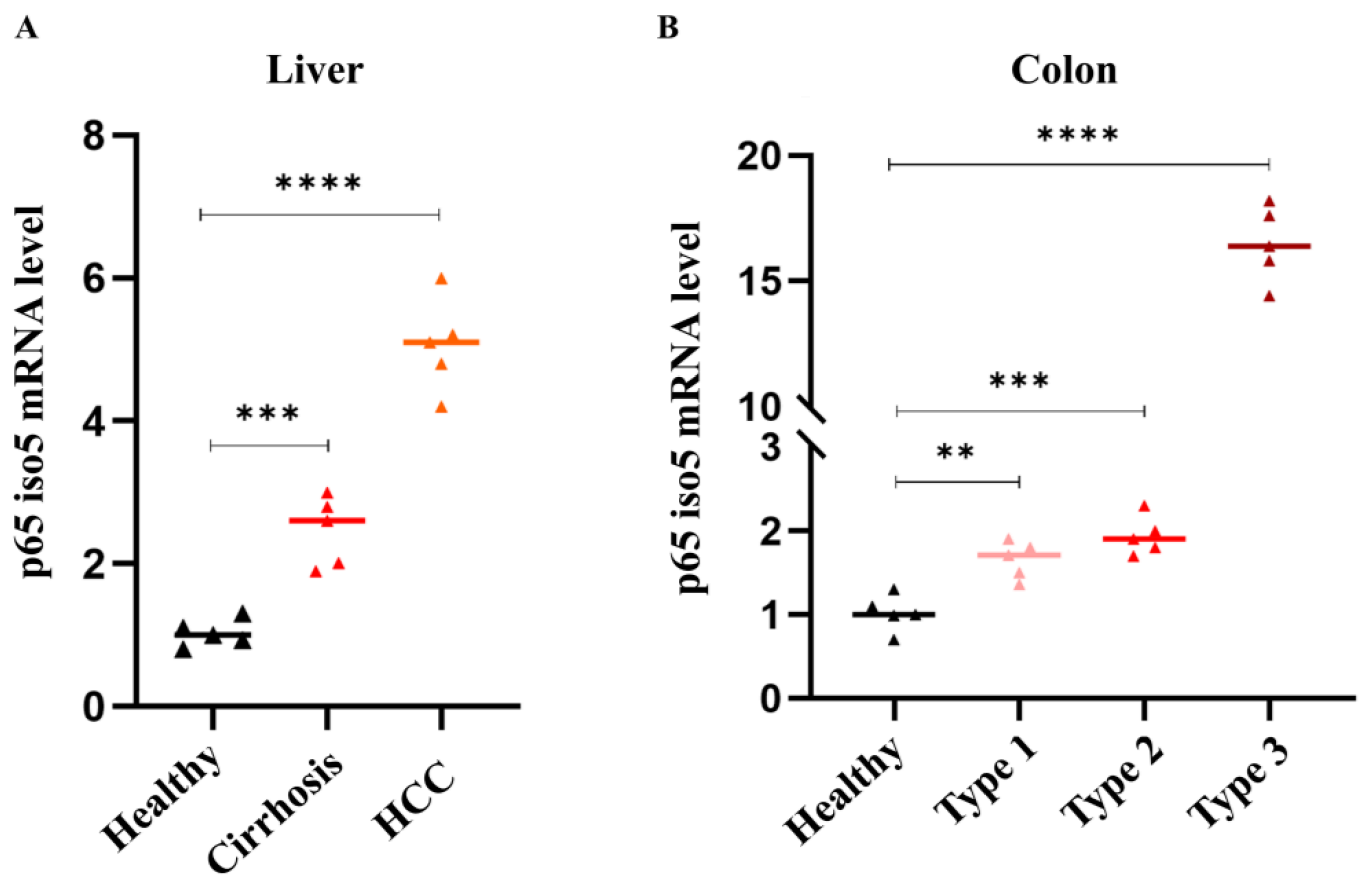
Upregulation of p65 iso5 mRNA in liver and colon samples with increasing disease state. (**A**) Relative mRNA expression levels of the p65 iso5 isoform in human liver tissues from healthy (*n* = 5), cirrhosis (*n* = 5), and HCC (*n* = 5) patients. (**B**) Relative mRNA expression levels of p65 iso5 in healthy colon tissue (*n* = 5) and different clinical stages of colon cancer (Type 1, *n* = 5; Type 2, *n* = 5; Type 3, *n* = 5). Data are presented as violin plots with lines indicating mean ± SEM. Statistical significance was assessed using one-way ANOVA with a post hoc test. ** *p* < 0.01, *** *p* < 0.001, **** *p* < 0.0001.

In solid tumors, NF-κB signaling, particularly through p65, is often constitutively activated at least at a low level, contributing to tumorigenesis and cancer progression. Overexpression of NF-κB components was associated with poor prognosis in various cancers, including liver and colorectal cancers [39]. NF-κB activation promotes tumor cell proliferation, survival, angiogenesis, and metastasis, highlighting its role in cancer development [40]. Given the importance of NF-κB in cancer biology, understanding the expression patterns and functional implications of its subunits, such as p65, is crucial for elucidating tumor mechanisms and identifying potential therapeutic targets. Recently, we identified a novel glucocorticoid-binding splice variant of p65, designated as p65 iso5, and found it to be overexpressed in human liver tissues affected by cirrhosis and HCC compared to healthy controls [12]. To further explore the potential clinical relevance and functional significance of p65 iso5 in cancer progression and differentiation, mRNA levels were evaluated in human liver and colon tumor tissues. Quantitative PCR (qPCR) analysis demonstrated a progressive increase in p65 iso5 mRNA in livers of patients affected by cirrhosis and hepatocellular carcinoma (HCC), compared to healthy subjects (Figure 1A). A significant upregulation in HCC tissues compared to both healthy and cirrhotic groups may indicate an association with disease severity. Colon samples, classified by differentiation grade as Type 1 (G1: well-differentiated, low grade), Type 2 (G2: moderately differentiated, intermediate grade), and Type 3 (G3: poorly differentiated, high grade) [41,42], showed a significantly increased p65 iso5 expression compared to healthy tissues, with a progressive elevation corresponding to tumor grade (Figure 1B). While this pattern suggests a potential involvement in inflammation-associated malignancies, the observed increase could also reflect a compensatory feedback mechanism aimed at counteracting the pro-inflammatory and potentially pro-tumorigenic activity of canonical p65. Therefore, whether p65 iso5 contributes to tumor progression or represents an adaptive regulatory response remains to be clarified. Furthermore, it has to be stated that the number of patient samples was limited, so further verification of our observation with larger cohorts would be necessary. To elucidate transcriptomic consequences of p65 iso5 expression, we performed RNA-seq analysis in HeLa cells overexpressing either p65 iso5 or canonical p65, in the presence or absence of dexamethasone, to compare their transcriptional regulatory programs.

### 3.2. Transcriptomics After Expression of GFP-Tagged p65 and p65 iso5 in HeLa Cells

**Figure 2:**
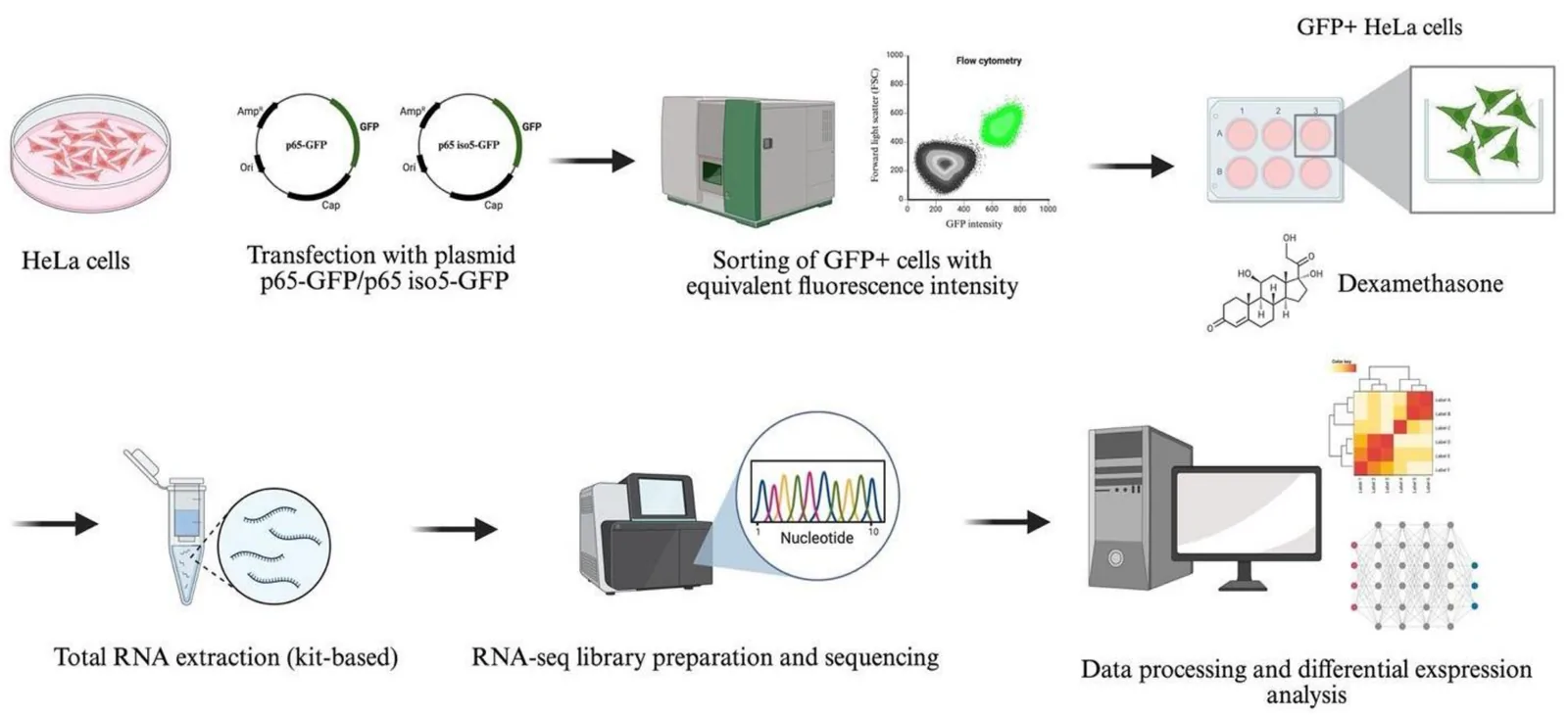
Experimental workflow for RNA-seq analysis of GFP-tagged p65 and p65 iso5 in HeLa cells. 1 day after transfection, GFP-positive cells with comparable moderate fluorescence intensities were sorted by flow cytometry and treated with Dexamethasone. Total RNA was extracted using a kit-based protocol and subjected to RNA sequencing (RNA-seq). Differential expression and pathway analyses were performed to compare the transcriptional profiles associated with p65 and p65 iso5 expression under identical cellular conditions. Created with BioRender.

**Figure 3:**
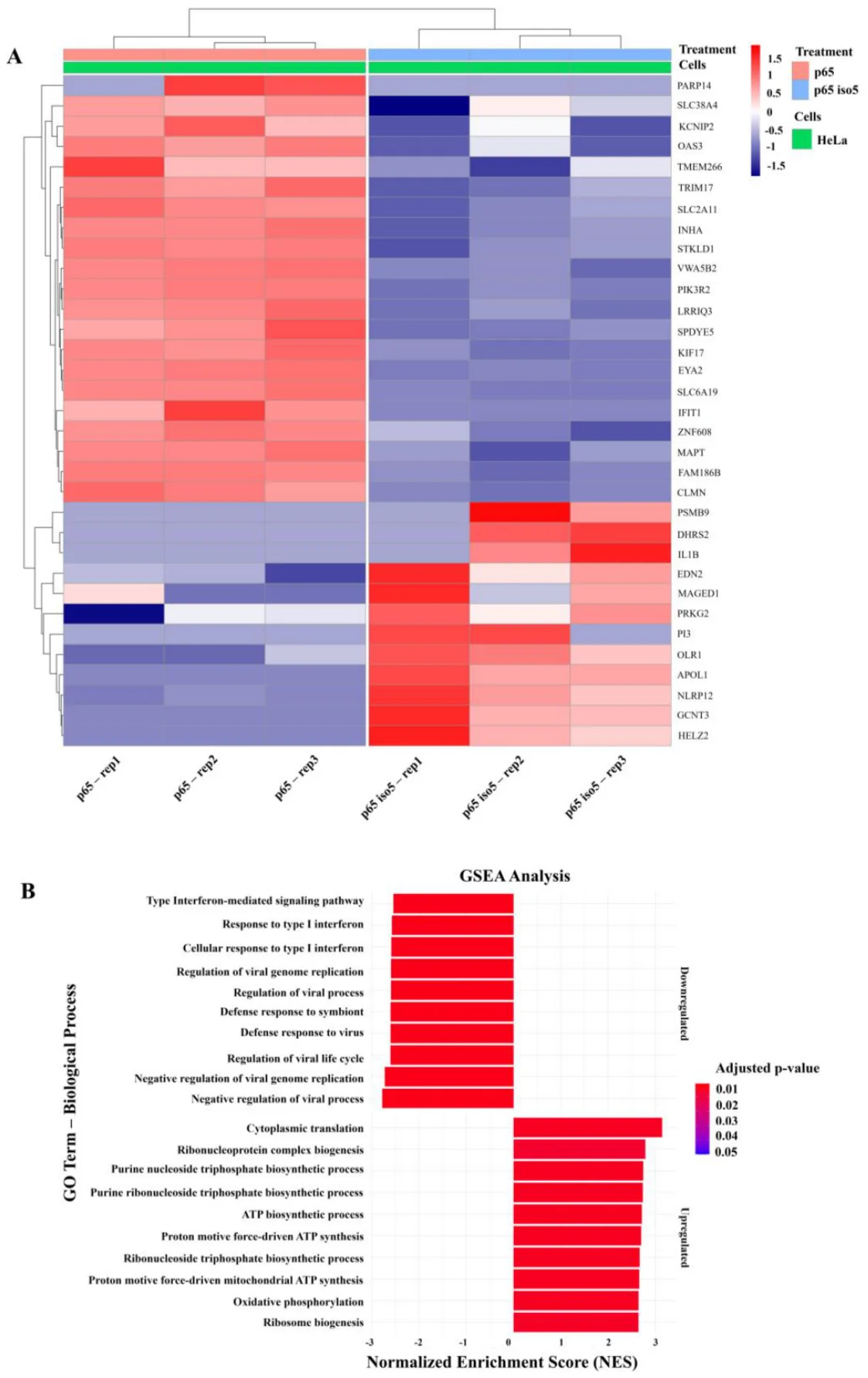
Differential gene expression and pathway enrichment analysis comparing the transcriptome after p65 iso5 expression with that after p65 expression in HeLa cells. (**A**). Heatmap of the top differentially expressed genes, illustrating sample clustering according to conditions. (**B**) Gene Set Enrichment Analysis (GSEA) bar plot showing significantly enriched GO terms.

To investigate the gene expression changes induced by p65 iso5 relative to canonical p65, HeLa cells were transiently transfected with expression constructs encoding either protein as a GFP-fusion protein. GFP-positive cells with comparable moderate fluorescence were sorted by flow cytometry (Figure S1), and part of each sample was subsequently treated with dexamethasone. Total RNA was then extracted, and libraries were prepared for RNA sequencing, followed by comprehensive bioinformatic analysis (Figure 2). After quality control of the RNA-sequencing, gene-level expression was quantified, and differential expression analysis was conducted using stringent cutoffs (adjusted *p*-value < 0.05 and log_2_ fold change > 1). Hierarchical clustering of differentially expressed genes confirmed clear segregation of samples, confirming the reproducibility of the transcriptional differences induced by p65 iso5 (Figure 3A). The above-mentioned thresholds for *p*-value and expression change revealed a significant upregulation of 28 genes, while 131 were significantly downregulated (Figure S2A and Table S1). Clustering of the top up- and downregulated genes further indicated that several canonical NF-κB target genes were only modestly affected or remained unchanged, suggesting that p65 iso5 selectively modulates a specific subset of NF-κB-dependent transcriptional programs rather than globally recapitulating canonical p65 activity. To obtain functional understanding of the observed transcriptional differences, Gene Set Enrichment Analysis (GSEA) was performed using the Gene Ontology (GO) Biological Process database. Downregulated genes in p65 iso5 cells as compared to canonical p65 were significantly enriched for pathways related to type I interferon signaling, antiviral defense, and negative regulation of viral genome replication and processing (Figure 3B), suggesting that p65 iso5 expression might exhibit a significantly reduced inflammatory potential [41]. In contrast, genes with higher expression in the presence of p65 iso5 (or downregulated by p65) were primarily associated with cytoplasmic translation, ribosome biogenesis, and ATP biosynthesis, suggesting a higher cellular metabolic and translational activity when compared to the effects of canonical p65 [42]. Representative GSEA enrichment plots further illustrate these findings. The type I interferon-mediated signaling pathway was strongly downregulated in p65 iso5-expressing cells when compared to canonical p65 (NES = −2.54, q = 2.214 × 10^−3^; Figure S2B), whereas cytoplasmic translation genes showed strong positive enrichment (NES = 3.15, q = 3.243 × 10^−3^; Figure S2C). Importantly, GSEA analysis of p65 iso5 versus GFP did not show significant enrichment of translation pathways, suggesting that the apparent increase in translation mainly derives from the translational repression effect of canonical p65. Overall, these results indicate that p65 iso5 elicits a weaker antiviral and inflammatory transcriptional program compared to canonical p65, while exerting a more limited impact on translational pathways.

### 3.3. Transcriptome Remodeling Induced by p65 iso5 upon Dexamethasone Stimulation

**Figure 4:**
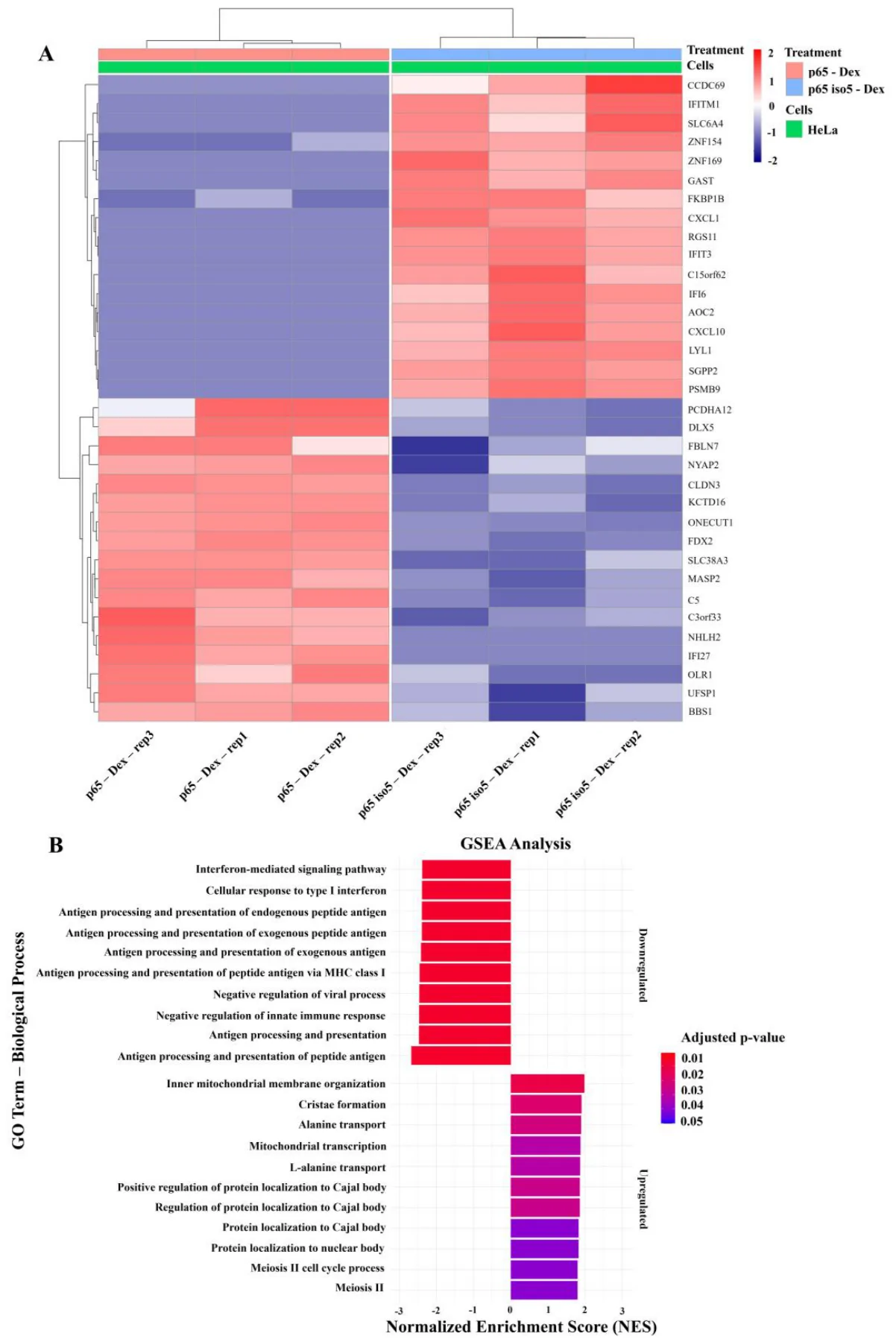
Differential gene expression and pathway enrichment analyses comparing p65 iso5 and canonical p65 in the presence of Dexamethasone (Dex). (**A**) Heatmap showing hierarchical clustering of differentially expressed genes (DEGs) between p65 iso5-Dex versus p65-Dex. Each column represents an independent biological replicate; the color scale indicates normalized counts scaled by row. (**B**) Gene Set Enrichment Analysis (GSEA) bar plot showing significantly enriched GO terms.

Differential gene expression analysis showed a deep remodeling of the cellular transcriptome in HeLa cells expressing p65 iso5 and treated with Dexamethasone (p65 iso5 Dex) compared with cells expressing the canonical p65 treated with Dexamethasone (p65 Dex) (Figure 4A). Hierarchical clustering analysis demonstrated a clear segregation between the two experimental groups, indicating that p65 iso5 expression is associated with a distinct transcriptional program in the presence of glucocorticoids. With a stringent setting for significance (|log_2_FC| > 1, adjusted *p* < 0.05), we found 75 genes upregulated and 86 downregulated (Figure S3), demonstrating a specific effect of p65 iso5 in the presence of glucocorticoids. Table S2 reports the list of DEGs. Gene Set Enrichment Analysis (GSEA) was subsequently performed to identify functionally enriched biological processes (Figure 4B). Negatively enriched pathways were predominantly related to immune and inflammatory signaling, including the interferon-mediated signaling pathway, cellular response to type I interferon, and antigen processing and presentation [43], indicating that p65 iso5 differs from canonical p65 also in the presence of Dex (Figure 4B and Figure S3A). Interestingly, negative regulation of innate immune response showed a negative enrichment score in p65 iso5 compared to p65, implying that glucocorticoids have a lower anti-inflammatory effect on p65 iso5, potentially because p65 iso5 itself is less pro-inflammatory than canonical p65. Pathways that were positively enriched in p65 iso5 in the presence of dexamethasone were associated with mitochondrial organization, metabolic regulation, and cell cycle processes. These findings indicate that canonical p65 activation not only stimulates immune gene expression but also suppresses mitochondrial and biosynthetic activities, potentially reflecting a cellular shift toward an inflammatory phenotype [44] (see also Figure S3B,C). Interestingly, p65 iso5 also upregulated genes involved in localization to Cajal bodies, which are important for the spliceosome, as well as telomerase function, and are related to high transcriptional and proliferative activity [45]. Collectively, these transcriptomic results demonstrate that p65 iso5 does not act in a pro-inflammatory manner, as canonical p65 does neither in the absence nor in the presence of Dexamethasone, but rather has roles in mitochondrial and metabolic processes involving ribosomes and spliceosomes when compared to the normal p65 isoform.

### 3.4. The Effect of Dexamethasone on p65 iso5 in HeLa Cells

**Figure 5:**
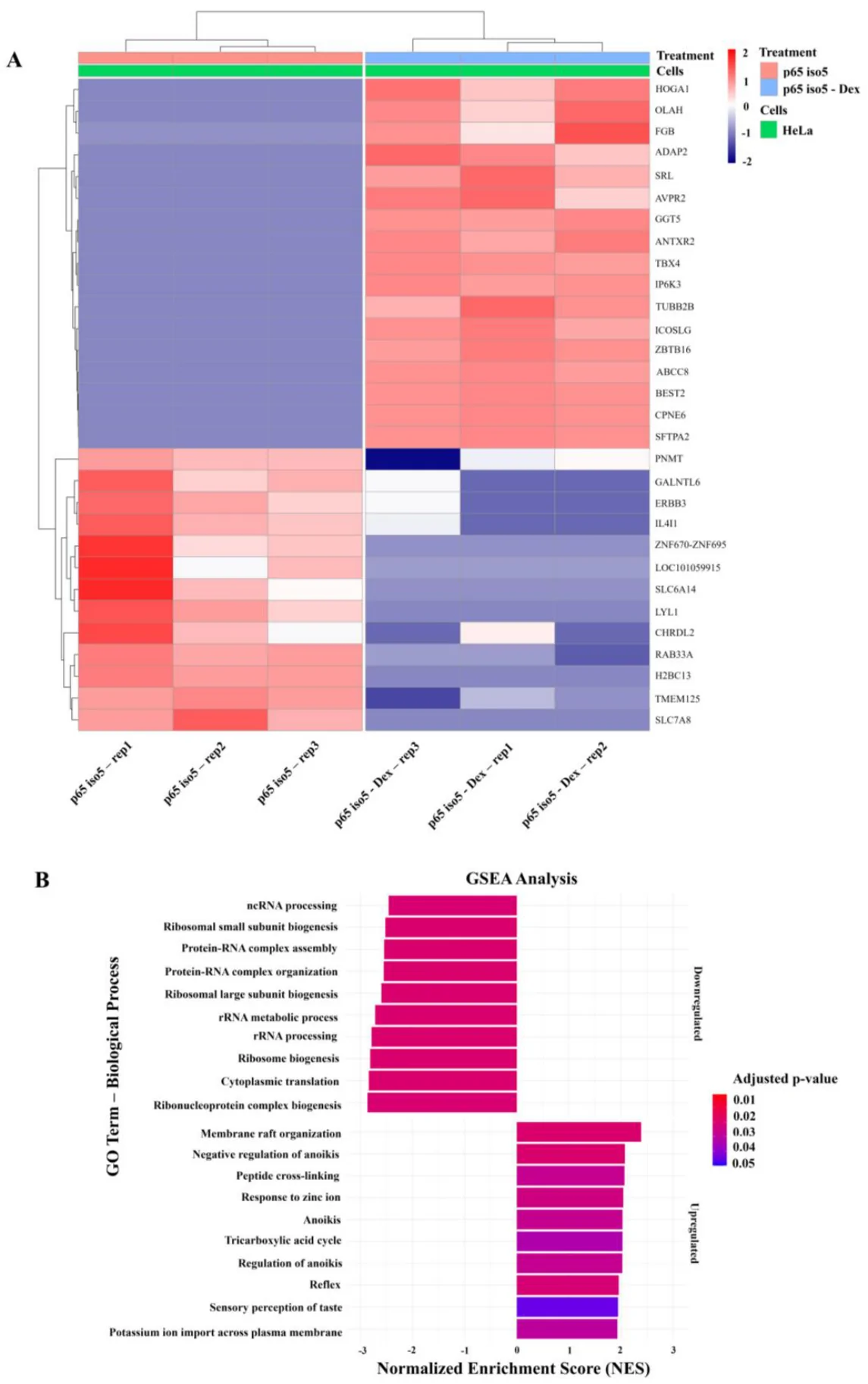
Transcriptional reprogramming shift induced by Dex in p65 iso5-expressing cells. (**A**) Heatmap illustrates the differentially expressed genes (DEGs) between p65 iso5-Dex and p65 iso5. Red indicates high expression, and blue indicates low expression. (**B**) Gene Set Enrichment Analysis (GSEA) of Gene Ontology (GO) Biological Processes. The bar chart shows the top-down regulated and upregulated pathways colored according to adjusted *p*-value.

HeLa cells with overexpression of p65 iso5 and treated with Dex showed a significantly different transcriptional profile than p65 iso5-expressing cells in the absence of the glucocorticoid. Indeed, the Heatmap (Figure 5A) and the Volcano Plot (Figure S4) highlight many genes that are significantly upregulated (*n* = 609) or downregulated (*n* = 183) (log2fold change > 1, adjusted *p* < 0.05, Table S3). Overall, Dex induced a broad reprogramming of the transcriptome, affecting multiple cellular processes critical for both proliferation and metabolism. Gene Set Enrichment Analysis (GSEA) (Figure 5B) demonstrated two major clusters of biological pathways significantly modulated by Dex. First, there was a pronounced downregulation of pathways supporting cell growth and biosynthesis, including ncRNA processing, ribosomal biogenesis, and protein-RNA complex assembly. The membrane raft organization gene set showed a highly positive normalized enrichment score (NES = 2.39, q = 1.65 × 10^−2^; Figure 5B), indicating strong upregulation. Notably, the ribonucleoprotein complex biogenesis displayed the most pronounced downregulation (lowest NES) among all analyzed pathways, indicating a strong reduction of the translational machinery in Dex-treated p65 iso5-expressing cells compared to p65 iso5-expressing controls [46,47]. This repression suggests that Dex effectively inhibits the transcriptional programs necessary for protein synthesis in p65 iso5-expressing cells. In contrast, Dex treatment led to a significant upregulation of pathways involved in the TCA cycle and of genes that inhibit anoikis (apoptosis triggered by loss of cell adhesion), reflecting a shift toward metabolic adaptation and matrix-independent growth. Notably, the ncRNA processing displayed a negative NES (NES = −2,46 q = 1.693 ×10^−2^, Figure 5B). This category encompasses the maturation and modification of non-coding RNAs, including rRNA, tRNA, snRNA, and snoRNA, which are essential for ribosome biogenesis, mRNA splicing, and RNA quality control. The repression of this pathway by Dex suggests that p65 iso5-Dex activation may attenuate RNA metabolic activity and nucleolar function, consistent with a cellular shift toward an inflammatory and stress-related phenotype [48]. Importantly, the comparison between Dex-treated and untreated cells highlights a dual effect of Dex in p65 iso5 cells: suppression of biosynthetic gene networks and simultaneous activation of metabolic and transport pathways. This shift suggests that Dex not only limits cell growth by downregulating protein synthesis but also enhances metabolic programs, potentially supporting cellular adaptation under glucocorticoid exposure. These findings highlight the ability of p65 iso5 to modulate the cellular response to Dex, linking NF-κB isoform expression to both transcriptional and metabolic reprogramming.

### 3.5. Multiple Comparisons for p65 and p65 iso5 in Presence and Absence of Dex

**Figure 6:**
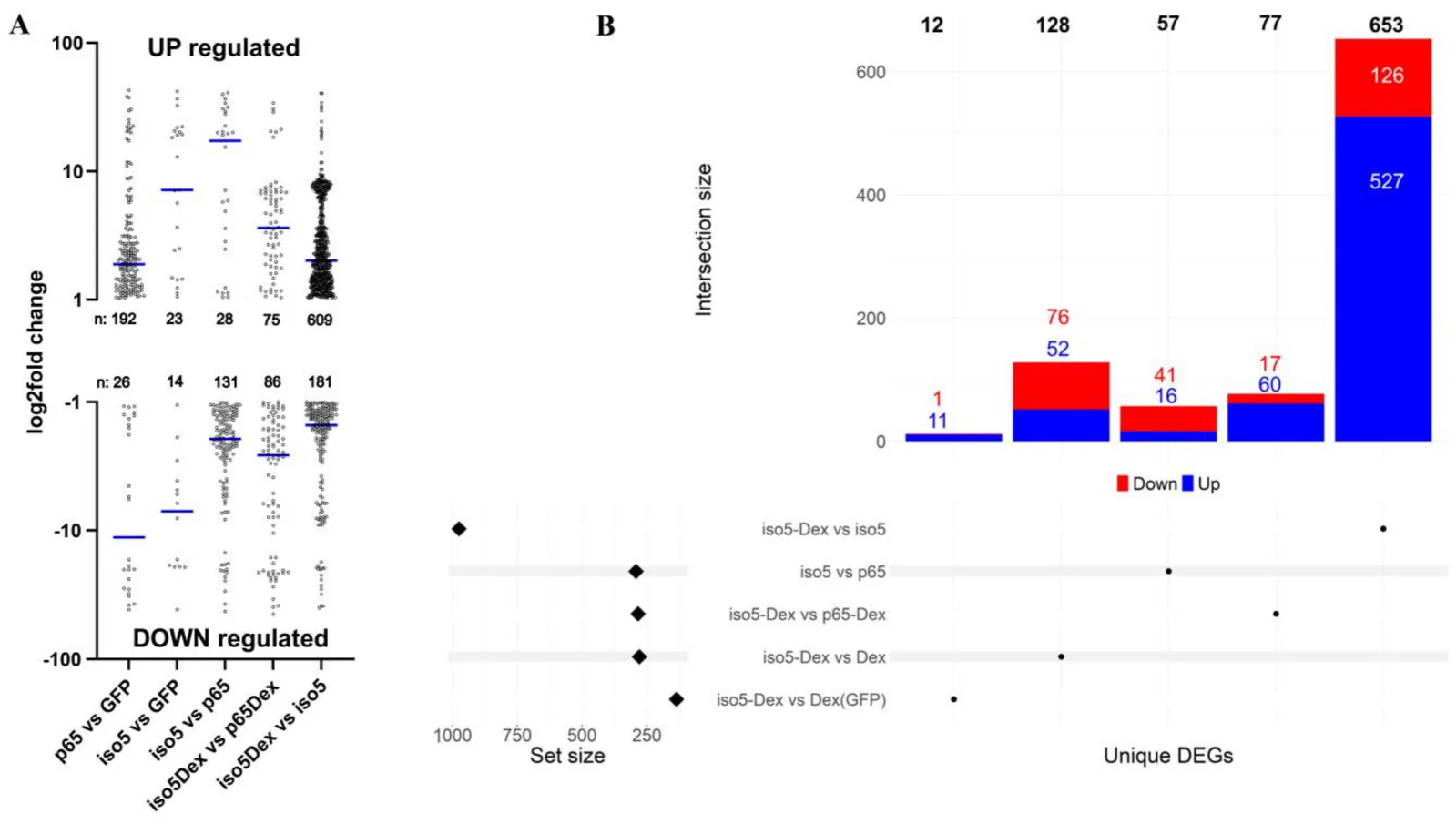
Multiple comparative analysis of differentially expressed genes in p65 iso5 and related conditions. (**A**) Scattergram of the significantly up- and downregulated genes for the different pairwise comparisons showing the levels of transcriptional changes, as well as the numbers of DEGs (*n*). (**B**) UpSet plot showing the unique Differentially Expressed Genes (DEGs only seen in one pairwise comparison) and their distribution across five keys pairwise comparisons: p65 iso5-Dex vs. p65 iso5 (iso5-Dex vs. iso5), p65 iso5 vs. p65 (iso5 vs. p65), p65 iso5-Dex vs. p65-Dex (p65 iso5-Dex vs. p65-Dex), p65 iso5-Dex vs. Hela-Dex (iso-Dex vs. Dex), p65 iso5-Dex vs. Hela-GFP-Dex (iso5-Dex vs. Dex (GFP)). The bar plot reports Unique DEGs, with red representing downregulated genes and blue representing upregulated genes. On the left is reported the total number of DEGs (set size) for each comparison.

For a better overview of the p65 iso5-specific transcriptional responses and effects of Dex, we performed a comparative analysis of Differentially Expressed Genes (DEGs) across multiple experimental conditions (Figure 6A,B). This approach allowed us to delineate both shared and unique gene expression changes associated with Dex treatment in cells expressing either the canonical p65 or the p65 iso5 protein. A comparison of up- and downregulated genes under the different conditions revealed that Dex treatment had a very significant effect on p65 iso5-expressing cells, resulting in the highest number of differentially expressed genes among the pairwise comparisons that were performed, while p65 iso5 expression in the absence of Dex affected only a rather small number of genes (Figure 6A).

**Figure 7:**
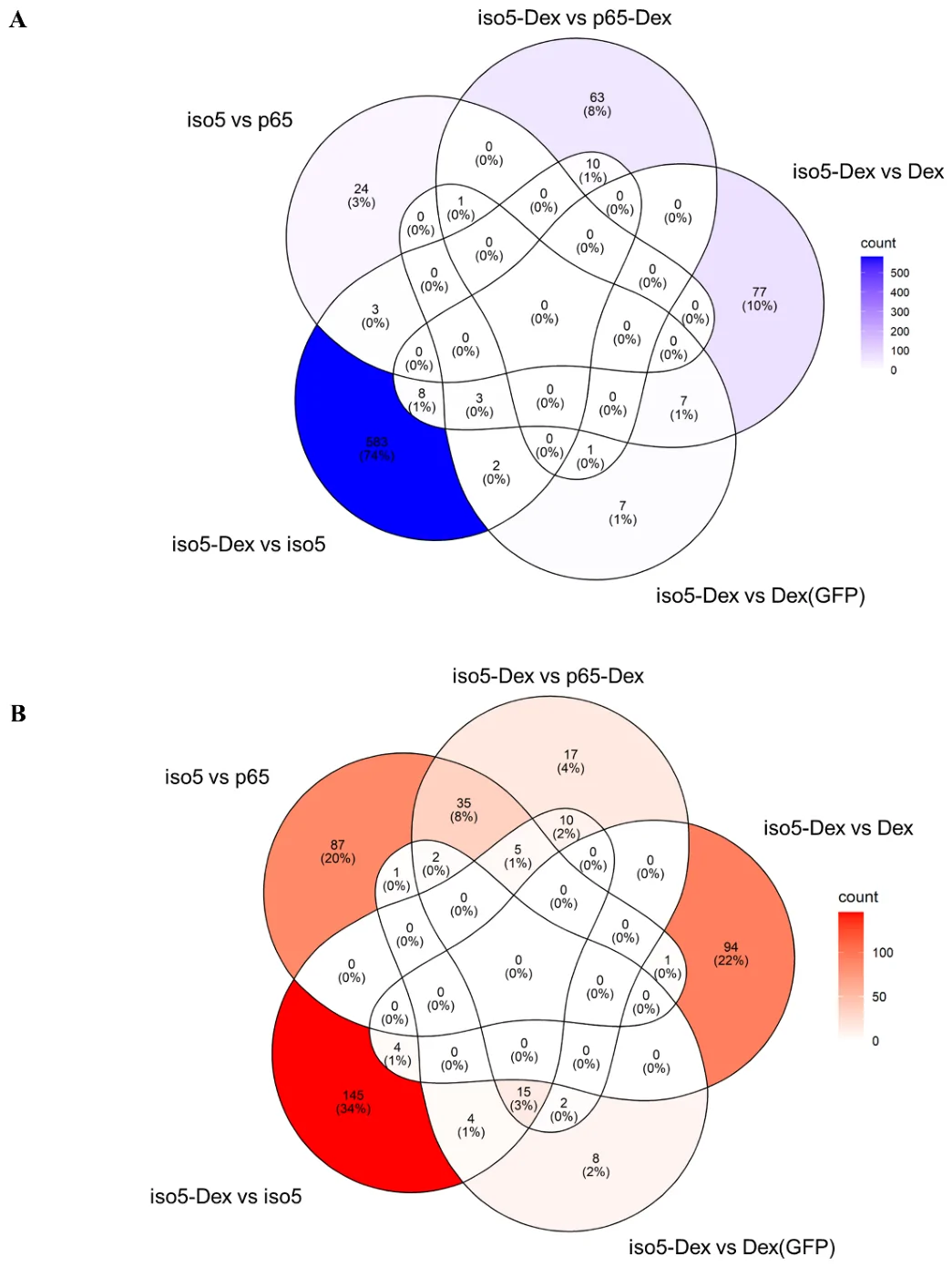
Differential gene expression profile between the analyzed experimental conditions. Each point represents an individual gene plotted according to its expression change and statistical significance. Genes significantly upregulated are highlighted in blue (**A**), whereas significantly downregulated genes are shown in red (**B**). The distribution reveals a clear separation between up- and down-regulated transcripts, indicating distinct transcriptional responses. Thresholds for significance and fold change were applied to identify differentially expressed genes, highlighting those most likely to contribute to the biological processes and pathways modulated under the studied conditions.

An UpSet plot (Figure 6B) was used to quantify the number of unique DEGs (genes that are regulated only in one pairwise comparison), in order to focus on genes selectively modulated in each experimental condition. The p65 iso5-Dex vs. Hela-Dex and p65 iso5-Dex vs. Dex (GFP) comparisons were specifically included to control for treatment and/or vector-related effects. For these two controls pairwise comparisons, we identified 12 (1 downregulated and 11 upregulated) and 128 (76 downregulated and 52 upregulated) uniquely differentially regulated genes, respectively. A total of 58 unique DEGs were identified for p65 iso5 vs. p65, including 41 downregulated and 16 upregulated unique genes. This specific transcriptional profile likely reflects the effect of p65 iso5 overexpression. The condition-specific transcriptomic comparison between p65 iso5- and p65-overexpressing cells under Dex stimulation (p65 iso5-Dex vs. p65-Dex) identified 78 uniquely differentially expressed genes, of which 17 were downregulated and 60 upregulated. The comparison between p65 iso5-Dex vs. p65 iso5 exhibited the largest number of uniquely regulated DEGs (*n* = 653), with 527 upregulated and 126 downregulated genes, highlighting the strong and specific impact of Dex on the transcriptome of the p65 iso5 isoform. Venn diagram analysis (Figure 7) shows additional information about the intersection between the five different comparisons. Interestingly, no DEGs were found in common across all the different comparisons. In addition, only a very limited number of DEGs were common across the different intersections between the comparisons. This finding indicates that the transcriptional response to Dex is largely isoform-dependent, with minimal overlap between the canonical p65 and the p65 iso5 protein. Furthermore, the direct comparison between p65 iso5-Dex and Dex identified a significant subset of 128 unique DEGs (~11% of total), demonstrating that p65 iso5 mediates a distinct transcriptional program in response to Dex that is not triggered by the canonical p65. The analysis of differentially expressed genes in the context of Dex treatment indicates that the transcriptional response to Dex is most pronounced in cells expressing p65 iso5. While individual differentially regulated genes in these comparisons provide limited additional mechanistic insight, the overall pattern suggests that the transcriptional program associated with p65 iso5 may be particularly responsive to glucocorticoid stimulation, pointing to a potential role of this isoform in shaping Dex-dependent transcriptional response. Additional information about unique DEGs for each condition is reported in Table S4.

### 3.6. Protein–Protein Interaction (PPI) Network Analysis of Differentially Expressed Genes

**Figure 8:**
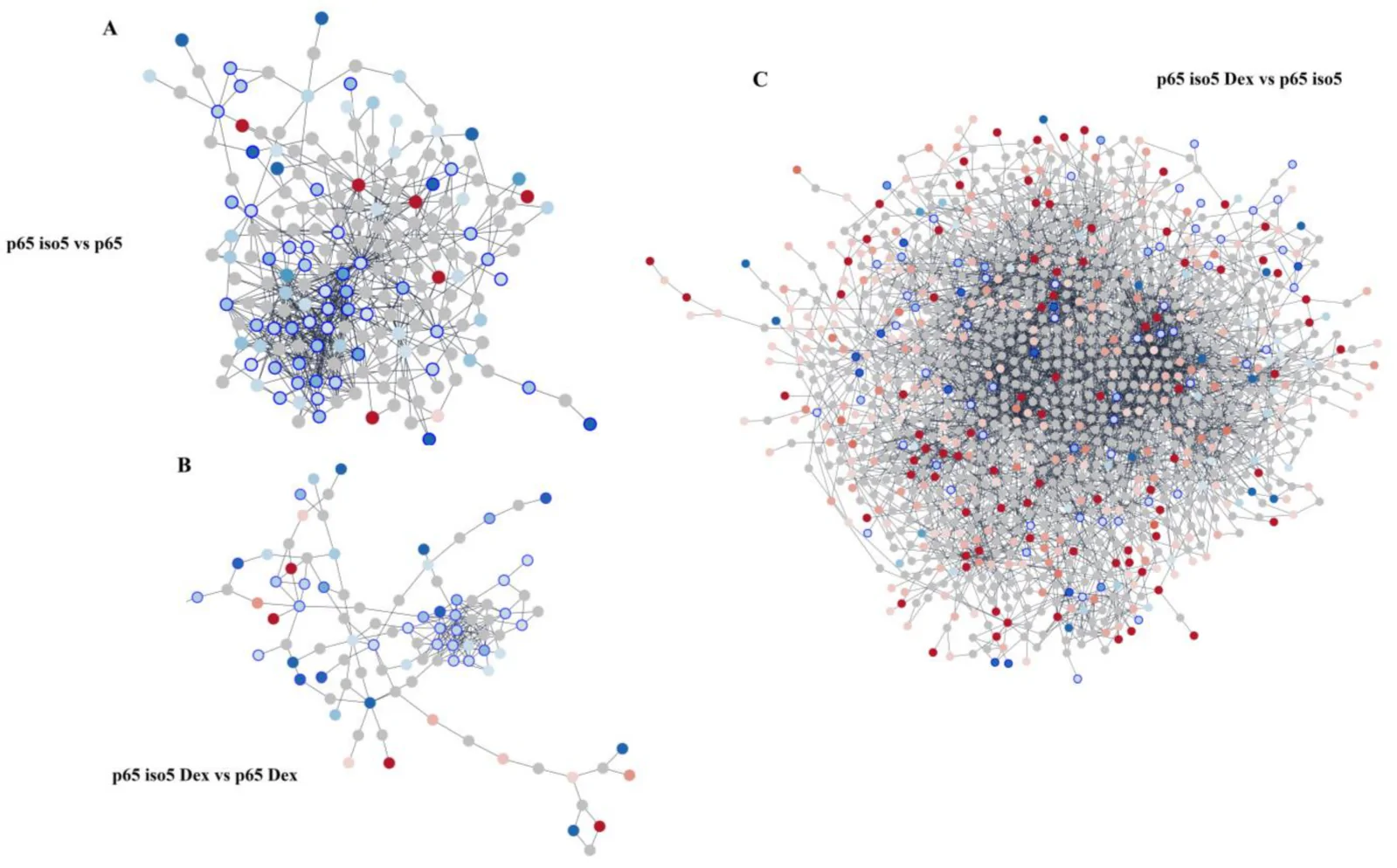
Protein–protein interaction (PPI) networks were constructed for genes that are differentially expressed only in specific pairwise comparisons. Nodes represent genes (proteins), and edges represent experimentally validated protein–protein interactions. Upregulated genes are shown in red, downregulated genes in blue (with fill color intensity proportional to log_2_ fold change), and non-differentially expressed genes (non-DEGs) are shown in grey. (**A**) PPI network of DEGs comparing p65 iso5 with p65 in the absence of dexamethasone, highlighting isoform-specific differences in gene regulation at baseline. Nodes with blue borders are genes of the GO-BP Immune response pathway (which had the lowest FDR value after functional enrichment of downregulated genes). (**B**) PPI network of DEGs for the comparison between p65 iso5 and p65 in the presence of Dex. Again, blue borders represent genes of the immune response. (**C**) PPI network of DEGs comparing p65 iso5 and p65 iso5-Dex, showing the dense interactome associated with Dex treatment of p65 iso5. Blue borders represent genes of the GO-BP term “Developmental process”.

To investigate the functional relationships among DEGs whose expression is specifically modulated in only one specific pairwise comparison, we performed protein–protein interaction (PPI) network analysis. The genes were uploaded to the NetworkAnalyst platform [37] and overlaid onto the protein interactome (STRING database), followed by calculation of the minimum network that links these genes. The network was downloaded as a graphml file and imported into the Cytoscape software, followed by functional enrichment analysis using the STRING App [49] (Figure 8). This analysis allowed us to explore potential molecular hubs and functional connectivity underlying isoform-specific transcriptional regulation. The protein interactome of differentially regulated genes that were unique to the comparison between p65 iso5 and p65 is shown in Figure 8A. This network displayed a moderate level of connectivity, forming a central gene cluster with both upregulated (red) and downregulated (blue) nodes. Despite being less dense, it highlighted key genes potentially serving as functional hubs, suggesting that even in the absence of Dex, p65 iso5 regulates a distinct set of protein interactions associated with specific biological processes. Importantly, genes related to the immune response pathway appeared downregulated, indicating that p65 iso5 has a lower inflammatory effect than p65. The PPI network obtained from differentially regulated genes for the comparison p65 iso5-Dex and p65-Dex is shown in Figure 8B, in order to capture the transcriptional differences in the presence of Dex. Several central hubs were selectively regulated in p65 iso5-Dex versus p65-Dex, implying that p65 iso5 mediates unique protein interaction patterns in response to glucocorticoid treatment. These hubs are likely critical nodes for coordinating downstream signaling and functional responses specific to the p65 iso5 protein. Finally, in Figure 8C, we show the interactome of all genes that are differentially regulated when comparing p65-iso5 with p65 iso5 in the presence of dexamethasone, reflecting the transcriptional impact of Dex specifically on the p65 iso5 isoform. This network exhibited the highest density and complexity, with numerous upregulated (red) and downregulated (blue) nodes forming a highly interconnected network. The extensive connectivity indicates that Dex triggers a coordinated reorganization of multiple signaling and metabolic pathways in p65 iso5-expressing cells, highlighting the isoform-specific nature of glucocorticoid-induced transcriptional remodeling. Collectively, these PPI analyses demonstrated that p65 iso5 not only regulates distinct sets of genes under basal conditions but also orchestrates a highly interconnected and functionally coordinated network in response to Dex, suggesting a central role for this isoform in modulating protein interactions that support both transcriptional and metabolic reprogramming.

### 3.7. p65 iso5 Interacts with Glucocorticoid Receptor in Presence of Dexamethasone

**Figure 9:**
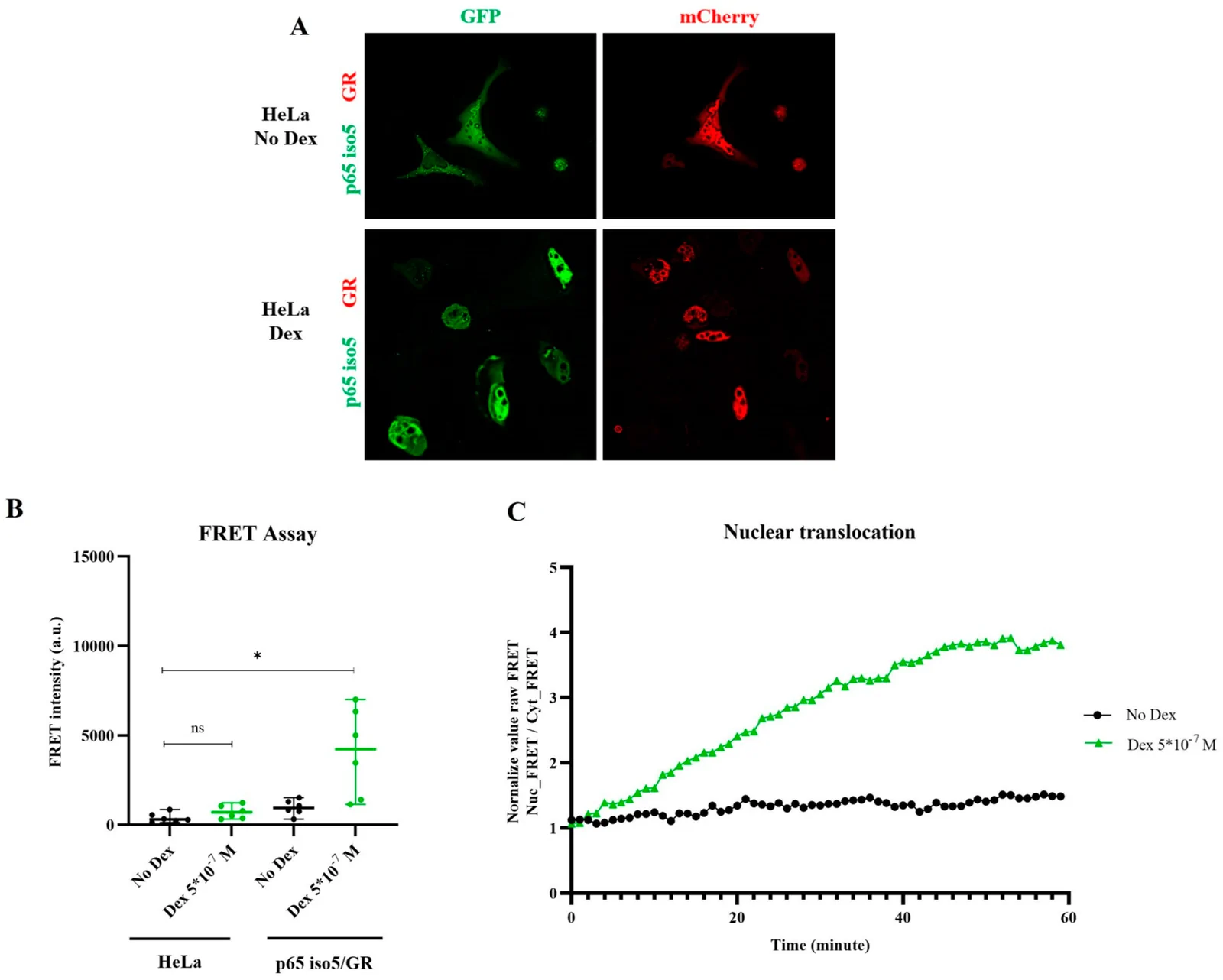
Protein interaction between p65 iso5 and GR as assessed by FRET microscopy. (**A**) HeLa cells were transfected with GFP-tagged p65 iso5 and mCherry-tagged GR, followed by FRET microscopy in the presence or absence of Dex (5 × 10^−7^ mol/L). (**B)** Quantification of FRET intensity under untreated (No Dex) and treated (Dex 5 × 10^−7^ M) conditions for control HeLa cells and p65 iso5/GR co-transfected cells (* *p* < 0.05, ns = not significant). (**C**) Nuclear translocation kinetics represented by the normalized ratio of nuclear to cytosolic FRET signal (Nuc_FRET/Cyt_FRET) recorded over time (0–60 min) upon addition of Dex (5 × 10^−7^ M) at time point 0 compared to control (No Dex).

We had previously shown that p65 iso5 can bind to dexamethasone as assessed by bioluminescence resonance energy transfer (BRET) between luciferase-conjugated p65 iso5 and FITC-labeled dexamethasone [12]. Since we found a strong effect of Dex on the transcriptional program of p65 iso5, we wanted to test whether p65 iso5 also binds to the glucocorticoid receptor GR and whether this putative binding depends on the presence of Dex. Recently, we provided biochemical and functional evidence that p65 iso5 physically associates with the GR, leading to the identification of a previously unrecognized p65 iso5/GR complex [50,51]. Here, we demonstrated not only the physical interaction between p65 iso5 and GR but also highlighted the functional relevance of this complex in shaping transcriptional responses. Given that in the present work we focus on the ability of p65 iso5 to regulate a set of genes that differ from those controlled by canonical p65 in the presence of Dex, it appeared particularly important to further investigate the behavior of this newly identified complex. Specifically, we aimed to characterize the dynamics of the p65 iso5/GR interaction upon Dex treatment in living cells, to better understand whether ligand binding influences complex formation, intracellular localization, and the selective transcriptional program driven by p65 iso5 under glucocorticoid stimulation. For that purpose, we expressed GFP-tagged p65 iso5 in combination with mCherry-conjugated GR and performed FRET microscopy in the absence and presence of Dex. Fluorescence microscopy revealed that in untreated cells both GFP-p65 iso5 and mCherry-GR were primarily cytosolic, whereas addition of Dex induced a prominent nuclear accumulation of both fusion proteins. The addition of Dex resulted in increased nuclear localization of p65 iso5 (Figure 9A). Analyzing the interaction between GFP-p65 iso5 and mCherry-tagged GR revealed a significant FRET signal between these proteins only in the presence of Dex (Figure 9B). Dexamethasone treatment resulted in a gradual increase of the nuclear FRET signal over time, reaching a plateau after about 45 min, indicating nuclear translocation of the p65 iso5/GR complex in the presence of Dex (Figure 9C).

## 4. Discussion

The present study identifies p65 iso5 as a transcriptionally and functionally distinct variant of the NF-κB subunit p65, capable of driving unique transcriptional and metabolic programs both under physiological conditions and following glucocorticoid treatment. Through RNA-seq, gene set enrichment, and protein interaction network analyses, we demonstrate that p65 iso5 does not simply reproduce the canonical NF-κB transcriptional program. Instead, it establishes distinct regulatory mechanisms that selectively modulate immune, metabolic, and stress-response pathways, in a manner that depends on the cellular context [52,53] and the presence of glucocorticoids. Under basal conditions, the transcriptomic profiling of HeLa cells transiently expressing GFP-tagged p65 iso5 revealed a robust difference from the effect of canonical p65. Differential expression and GSEA analyses indicated a lack of induction of type I interferon-mediated signaling, antiviral defense responses, and pathways regulating viral genome replication when compared to canonical p65, suggesting that p65 iso5 does not induce innate immune signaling and antiviral gene expression as canonical p65 does. RNAs that were expressed at higher levels in p65 iso5 as compared to p65 (which are normally downregulated by p65 were significantly enriched in biological processes associated with ribosome biogenesis, translation, and ATP biosynthesis, consistent with metabolic reorientation toward biosynthetic activity and energy production. This shift suggests that p65 iso5 expression is associated with a transcriptional state characterized by enhanced biosynthetic and anabolic metabolism as compared to p65-expressing cells. The reciprocal regulation of antiviral and metabolic pathways suggests that p65 iso5 may act as a molecular switch that tunes NF-κB signaling toward metabolic rather than inflammatory outputs. The use of HeLa cells in the present study was intended to provide a controlled and reproducible experimental system for comparing the transcriptional activity of p65 iso5 and canonical p65, particularly by allowing the efficient transfection and flow-cytometric sorting of GFP-positive cells with comparable expression levels in this well-established cancer line, based on the observation that p65 iso5 expression was shown to be elevated in cancer. However, given the cell-type-dependent nature of NF-κB signaling, the effects of p65 iso5 observed in HeLa cells cannot be directly extrapolated to immune cells. Future studies in immune-cell models, including THP-1 cells or primary immune cells, will be important to determine whether the transcriptional and functional properties of p65 iso5 described here are similar in immune cells and to further define its role in inflammatory and immune responses. p65 iso5 may also serve as a feedback mechanism to reduce inflammatory signaling that is normally exerted by canonical p65—and could therefore also serve as a protective counterbalance to inflammation. The increase of this isoform with an increasing pathological state both in liver and in colon cancer—two malignancies that are strongly associated with inflammation—might also mean that the organism tries to escape the tumor-driving inflammatory state by upregulating a p65 isoform, which is less pro-inflammatory. Upon Dex stimulation, the transcriptional program driven by p65 iso5 showed extensive remodeling, diverging drastically from that of canonical p65 characterized by suppression of ribosome biogenesis and translation and thus proliferative and biosynthetic pathways, while TCA-cycle and mitochondria-related genes were upregulated, suggesting a transcriptional shift toward pathways associated with mitochondrial metabolism and metabolic flexibility. Dex treatment in p65 iso5-expressing cells resulted in a profound reduction of interferon-regulated and antiviral genes when compared to canonical p65. These findings indicate that p65 iso5 expression is associated with a specific glucocorticoid-responsive transcriptional program. These transcriptional changes are consistent with the activation of pathways associated with cellular stress responses and adaptation. At the same time, Dex treatment caused the downregulation of genes associated with proliferation, ribosomal biogenesis, and ncRNA processing, suggesting a global suppression of anabolic metabolism and cellular growth [54,55]. From a mechanistic perspective, the distinct response of p65 iso5 to Dex may be related to the structural and interaction properties conferred by its additional upstream exon. We demonstrated that p65 iso5 is able to physically bind Dex and form a complex with the glucocorticoid receptor (GR). Importantly, following Dex treatment, the p65 iso5-GR complex showed a predominantly nuclear localization, supporting a direct role of this interaction in the transcriptional response to glucocorticoids. These findings provide a possible mechanistic basis for the marked transcriptional remodeling observed in the present study. We hypothesize that Dex binding and the subsequent association of p65 iso5 with GR may alter the regulatory protein–protein interaction landscape of p65 iso5 and promote the recruitment of distinct transcriptional cofactors, thereby shifting its transcriptional output from the basal metabolic and less inflammatory state toward a distinct glucocorticoid-responsive program. This mechanism may account for the apparent switch-like behavior of p65 iso5 observed following Dex stimulation. However, although our functional and interaction data support this model, the present study does not provide direct structural evidence of a Dex-induced conformational change in p65 iso5. Therefore, further structural and biochemical studies will be required to determine whether Dex binding induces specific conformational changes that modify the interaction interfaces of p65 iso5 with GR and other transcriptional regulators. Comparative analyses of Dex-induced differential expression across multiple experimental conditions revealed substantial differences between the transcriptional responses associated with p65 and p65 iso5. The UpSet and Venn diagram analyses showed that only a limited number of DEGs were shared between conditions, while a substantial proportion of Dex-responsive genes were unique to the p65 iso5 background. These findings highlight differences in the transcriptional responses associated with the two p65 isoforms following Dex treatment, although they do not constitute a formal statistical test of an isoform-by-treatment interaction. Notably, the comparison between p65 iso5-Dex and p65-Dex identified 128 uniquely regulated genes (~11% of total DEGs), highlighting differences in the transcriptional regulation of processes related to metabolism, cellular stress adaptation, and proliferation. These findings support the concept that p65 iso5 is associated with distinct transcriptional plasticity following glucocorticoid treatment, potentially contributing to differences in the balance between glucocorticoid-mediated suppression and NF-κB-associated activation of immune genes. At the systems level, protein–protein interaction (PPI) network analyses provided further mechanistic insight into the functional reorganization mediated by p65 iso5. Under physiological conditions, the p65 iso5 network exhibited moderate connectivity, highlighting discrete gene clusters with both up- and downregulated nodes associated with translation and metabolic control. In contrast, Dex-treated p65 iso5 cells displayed highly interconnected and complex PPI networks, characterized by significantly connected hubs. This suggests that Dex amplifies isoform-specific signaling complexity, promoting a coordinated and multi-layered regulation of gene networks that support both immune regulation and metabolic adaptation. Such dynamic remodeling of PPI connectivity by p65 iso5 underscores its potential to act as a central integrator of NF-κB and glucocorticoid signaling, reshaping transcriptional and functional outcomes depending on the cellular environment. From a clinical and disease-associated perspective, the observed overexpression of p65 iso5 in human liver tissues affected by cirrhosis and HCC, together with its progressive increase across colon cancer states and its correlation with tumor grade, provides preliminary evidence of an association between p65 iso5 expression and disease severity. However, given the limited size of the human tissue cohorts analyzed, these findings should be considered exploratory and disease-associated observations rather than evidence of a causal role for p65 iso5 in tumor progression or of established clinical relevance. The increased expression of p65 iso5 may reflect a disease-associated response to the inflammatory and metabolic environment of the tumor, or alternatively a compensatory mechanism aimed at modulating the activity of canonical NF-κB signaling. Further studies using larger, independently characterized patient cohorts, together with disease-relevant experimental models and endogenous p65 iso5 expression, will be required to determine the robustness and clinical significance of these associations and to establish whether p65 iso5 contributes directly to tumor progression or represents an adaptive response to the pathological environment. It is important to note that canonical NF-κB/p65 signaling has also been extensively implicated in liver and colorectal malignancies, supporting the relevance of the NF-κB pathway in tumor-associated inflammation and cancer progression. However, since the present study specifically focused on the expression pattern of p65 iso5, canonical p65 expression was not quantitatively assessed in the same tissue specimens, and therefore the relative contribution of the two isoforms to the observed malignant phenotype cannot be established from the current data. Moreover, the progressive increase of p65 iso5 mRNA from healthy colon samples towards cancer states, coupled with its correlation with tumor grade, suggests a potential role in disease progression, which could either be a disease-driving role or alternatively a negative feedback mechanism by which the organism tries to escape from the malignant transformation. Given the dual role of NF-κB in both promoting inflammation and supporting tumor metabolism, the increasing isoform-specific activities of p65 iso5 could contribute to the establishment of a microenvironment that influences tumor survival under metabolic or inflammatory stress, given that p65 iso5 does not lead to a significant inflammatory response and thus presumably a reduced tumor immune surveillance. The association of p65 iso5 expression with reduced antiviral and inflammatory transcriptional responses and enhanced metabolic activity may be relevant to the tumor microenvironment, although whether these changes directly contribute to tumor growth remains to be established. Generally, its Dex-induced reprogramming may reflect a mechanism of cellular adaptation to pharmacological or hormonal stress in the tumor environment. Overall, our findings demonstrated that p65 iso5 acts as a functionally distinct NF-κB isoform that operates at the intersection of immune regulation, metabolism, and stress adaptation. Future studies should explore its role in tissue-specific responses, its interaction with other transcriptional regulators, and its potential utility in precision medicine strategies targeting the NF-κB-glucocorticoid crosstalk. Importantly, the context-dependent functions of p65 iso5, together with its increased expression in disease-associated liver and colon tissues, support further investigation of its potential relevance in pathological conditions involving dysregulated NF-κB signaling. However, given the preliminary and exploratory nature of the present findings, whether p65 iso5 may ultimately represent a useful biomarker or therapeutic target remains to be determined through validation in larger, independently characterized clinical cohorts and through complementary functional studies. An important limitation of the present study is that the transcriptomic analyses were performed in HeLa cells transiently overexpressing GFP-tagged p65 or p65 iso5. Although GFP-based sorting allowed the selection of cell populations with comparable and moderate reporter fluorescence and thus isoform expression, GFP intensity cannot be considered a direct quantitative measure of the functional activity of the corresponding fusion proteins. Although this experimental system allowed a controlled comparison of the transcriptional properties of the two isoforms, the observed changes represent isoform-associated transcriptional programs under the experimental conditions used and cannot by themselves establish the endogenous physiological or pathological functions of p65 iso5. Therefore, the potential contribution of p65 iso5 to disease progression and its relevance in specific cell types should be further investigated using endogenous expression systems and disease-relevant cellular models. Overall, our findings support p65 iso5 as a functionally distinct NF-κB isoform associated with distinct transcriptional programs at the intersection of immune regulation, metabolism, and stress adaptation. By being associated with distinct transcriptional programs, p65 iso5 may contribute an additional layer of complexity to NF-κB signaling and cellular homeostasis.

## 5. Conclusions

In summary, our findings identify p65 iso5 as a transcriptionally and functionally distinct isoform of the NF-κB subunit p65 that significantly reorganizes cellular gene expression and signaling networks. Through integrative RNA-seq analysis, pathway enrichment, and PPI analyses, we demonstrate that p65 iso5 establishes a unique transcriptional program that differs from canonical p65, selectively modulating immune, metabolic, and stress-response pathways in a stimulus-dependent manner. Upon glucocorticoid exposure, p65 iso5 led to a transcriptional reprogramming characterized by suppression of interferon-regulated genes and activation of proliferative and biosynthetic pathways, indicating an adaptive transition towards an energy-conserving phenotype. The isoform-specific responses observed across all experimental conditions highlight the capacity of p65 iso5 to fine-tune NF-κB and glucocorticoid signaling crosstalk, thereby expanding the regulatory versatility of the NF-κB system. Furthermore, the progressive upregulation of p65 iso5 in liver cirrhosis, HCC, and colon tumors, and the correlation with the grade of de-differentiation suggests that this protein could play a role in tumor progression and metabolic adaptation to chronic stress conditions either as an activator or a feedback inhibitor. Taken together, our results identify p65 iso5 as a context-dependent NF-κB isoform associated with distinct transcriptional programs linking inflammation, metabolism, and cellular stress adaptation. Its increased expression in disease-associated tissues warrants further investigation of its potential relevance as a biomarker and therapeutic target in NF-κB-related pathologies.

## Figures and Tables

**Table 1:** Oligonucleotides used in qPCR analysis for p65 iso5.

Target	Forward Primer	Reverse Primer	Amplified (bp)
p65 iso5	AGCCCTGGCTGCTCCAGACC	CCGGGAAGATGAGGGGGAAC	115

## Data Availability

The original contributions presented in this study are included in the article and Supplementary Materials. The raw RNA-seq data generated and analyzed during the current study are available in the Gene Expression Omnibus (GEO) database under accession number GSE346671 (https://www.ncbi.nlm.nih.gov/geo/query/acc.cgi?acc=GSE346671, accessed on 28 June 2026).

## Supplementary Materials

The following supporting information can be downloaded at: https://www.mdpi.com/article/10.3390/cells15181643/s1, Supplementary Figure S1. Flow cytometry gating strategy for identification and sorting of GFP-positive HeLa cell populations. (A) Representative density plots of HeLa cells transfected with GFP alone (left), p65_GFP (middle), or p65 iso5_GFP (right). The main cell population (red) was defined based on physical parameters (FSC-H vs. SSC-H) to exclude debris and aggregates. (B) Sorting strategy applied to the pre-gated population. Cells (green) were fur-ther selected based on GFP fluorescence intensity to isolate GFP-positive populations, including those expressing p65 and p65 iso5 constructs. Supplementary Figure S2. Transcriptomic analysis of p65 iso5 versus p65. (A) Volcano plot of DEGs highlighting upregulated (red) and downregulated (blue) transcripts following treatment. (B) Cytoplasmic translation enrich-ment plot (NES = 3.15, q-value < 0.01). (C) Type I interferon-mediated signaling pathway enrichment plot (NES = −2.54, q-value < 0.001). Abbreviations: NES, normalized enrichment score. Supplementary Figure S3. Transcriptomic analysis of p65 iso5 versus p65 following dexamethasone treatment. (A) Volcano plot of DEGs highlighting upregulated (red) and downregulated (blue) transcripts following treatment. (B) Inner mitochondrial membrane organization enrichment plot (NES = 1.98, q-value < 0.01). (C) Interferon-mediated signaling pathway enrichment plot (NES = −2.39, q-value < 0.001). Abbreviations: NES, normalized enrichment score. Supplementary Figure S4. Transcriptomic analysis of p65 iso5 cells following dexamethasone treatment compared with untreated p65 iso5 cells. (A) Volcano plot of DEGs highlighting upregulated (red) and downregulated (blue) transcripts following Treatment. (B) Membrane raft organization enrichment plot (NES = 2.39 q-value < 0.01). (C) ncRNA processing enrichment plot (NES= −2.46, q-value < 0.001). Abbreviations: NES, normalized enrichment score. Table S1. Differentially expressed genes identified by RNA-sequencing analysis. List of genes differentially expressed in HeLa cells expressing GFP-tagged p65 iso5 compared with cells expressing GFP-tagged canonical p65. Differentially expressed genes were identified using an adjusted *p*-value < 0.05 and a log2 fold change > 1. A total of 159 genes met these criteria, including 28 significantly upregulated and 131 significantly downregulated genes in p65 iso5 expressing cells compared with canonical p65 expressing cells. Table S2. Differentially expressed genes identified by RNA-sequencing analysis following dexamethasone stimulation. List of genes differentially expressed in HeLa cells expressing GFP-tagged p65 iso5 compared with cells expressing GFP-tagged canonical p65 following dexamethasone treatment. Differentially expressed genes were identified using a stringent threshold of log2 fold change > 1 and adjusted *p*-value < 0.05. A total of 161 differentially expressed genes were identified, including 75 significantly upregulated and 86 significantly downregulated genes in p65 iso5 Dex-expressing cells compared with p65 Dex expressing cells. Table S3. Differentially expressed genes induced by dexamethasone treatment in p65 iso5-expressing HeLa cells. List of genes differentially expressed in HeLa cells expressing GFP-tagged p65 iso5 following dexamethasone treatment compared with untreated p65 iso5 expressing cells. Differentially expressed genes were identified using a log2 fold change > 1 and adjusted *p*-value < 0.05. A total of 792 differentially expressed genes were identified, including 609 significantly upregulated and 183 significantly downregulated genes following dexamethasone treatment. Table S4. Unique differentially expressed genes identified across multiple experimental comparisons. List of unique differentially expressed genes (DEGs) identified across the pairwise comparisons of p65- and p65 iso5-expressing HeLa cells in the presence or absence of dexamethasone. Unique DEGs were defined as genes differentially regulated in only one of the analyzed pairwise comparisons. The table includes the unique DEGs identified for p65 iso5 vs. p65, p65 iso5-Dex vs. p65-Dex, p65 iso5-Dex vs. p65 iso5, p65 iso5-Dex vs. HeLa-Dex, and p65 iso5-Dex vs. Dex (GFP).
