# Supplementary material for: The NF-κB Isoform p65 iso5 Is Associated with Distinct Transcriptional Programs and Signaling Pathways
Source: Cells. 2026 Sep 10;15(18):1643. doi: 10.3390/cells15181643 (PMC13605157; doi:10.3390/cells15181643)
Supplement: Supplementary file 1 [file cells-15-01643-s001.zip › cells-4499562-Supplementary tables captions.pdf]

Table S1. Differentially expressed genes identified by RNA-sequencing analysis. List of genes differentially expressed in HeLa cells expressing GFP-tagged p65 iso5 compared with cells expressing GFP-tagged canonical p65. Differentially expressed genes were identified using an adjusted p-value < 0.05 and a log2 fold change > 1. A total of 159 genes met these criteria, including 28 significantly upregulated and 131 significantly downregulated genes in p65 iso5 expressing cells compared with canonical p65 expressing cells.

Table S2. Differentially expressed genes identified by RNA-sequencing analysis following dexamethasone stimulation. List of genes differentially expressed in HeLa cells expressing GFP-tagged p65 iso5 compared with cells expressing GFP-tagged canonical p65 following dexamethasone treatment. Differentially expressed genes were identified using a stringent threshold of log2 fold change > 1 and adjusted *p*-value < 0.05. A total of 161 differentially expressed genes were identified, including 75 significantly upregulated and 86 significantly downregulated genes in p65 iso5 Dex-expressing cells compared with p65 Dex expressing cells.

Table S3. Differentially expressed genes induced by dexamethasone treatment in p65 iso5-expressing HeLa cells. List of genes differentially expressed in HeLa cells expressing GFP-tagged p65 iso5 following dexamethasone treatment compared with untreated p65 iso5 expressing cells. Differentially expressed genes were identified using a log2 fold change > 1 and adjusted p-value < 0.05. A total of 792 differentially expressed genes were identified, including 609 significantly upregulated and 183 significantly downregulated genes following dexamethasone treatment.

Table S4. Unique differentially expressed genes identified across multiple experimental comparisons. List of unique differentially expressed genes (DEGs) identified across the pairwise comparisons of p65- and p65 iso5-expressing HeLa cells in the presence or absence of dexamethasone. Unique DEGs were defined as genes differentially regulated in only one of the analyzed pairwise comparisons. The table includes the unique DEGs identified for p65 iso5 vs. p65, p65 iso5-Dex vs. p65-Dex, p65 iso5-Dex vs. p65 iso5, p65 iso5-Dex vs. HeLa-Dex, and p65 iso5-Dex vs. Dex (GFP).
