# Supplementary material for: The NF-κB Isoform p65 iso5 Is Associated with Distinct Transcriptional Programs and Signaling Pathways
Source: Cells. 2026 Sep 10;15(18):1643. doi: 10.3390/cells15181643 (PMC13605157; doi:10.3390/cells15181643)
Supplement: Supplementary file 1 [file cells-15-01643-s001.zip › Supplementary materials Spinelli et al.pdf]

Supplementary materials

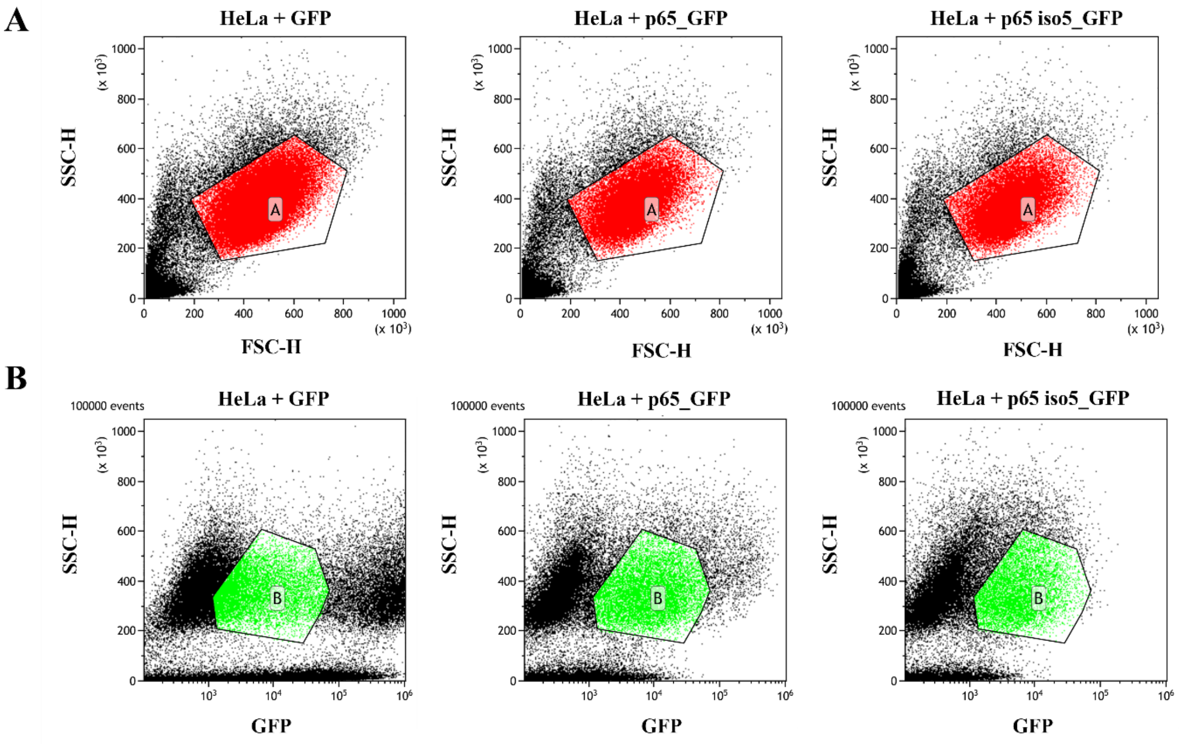

Supplementary figure S1. Flow cytometry gating strategy for identification and sorting of GFP-positive HeLa cell populations. **(A)** Representative density plots of HeLa cells transfected with GFP alone (left), p65_GFP (middle), or p65 iso5_GFP (right). The main cell population (red) was defined based on physical parameters (FSC-H vs SSC-H) to exclude debris and aggregates. **(B)** Sorting strategy applied to the pre-gated population. Cells (green) were further selected based on GFP fluorescence intensity to isolate GFP-positive populations, including those expressing p65 and p65 iso5 constructs.

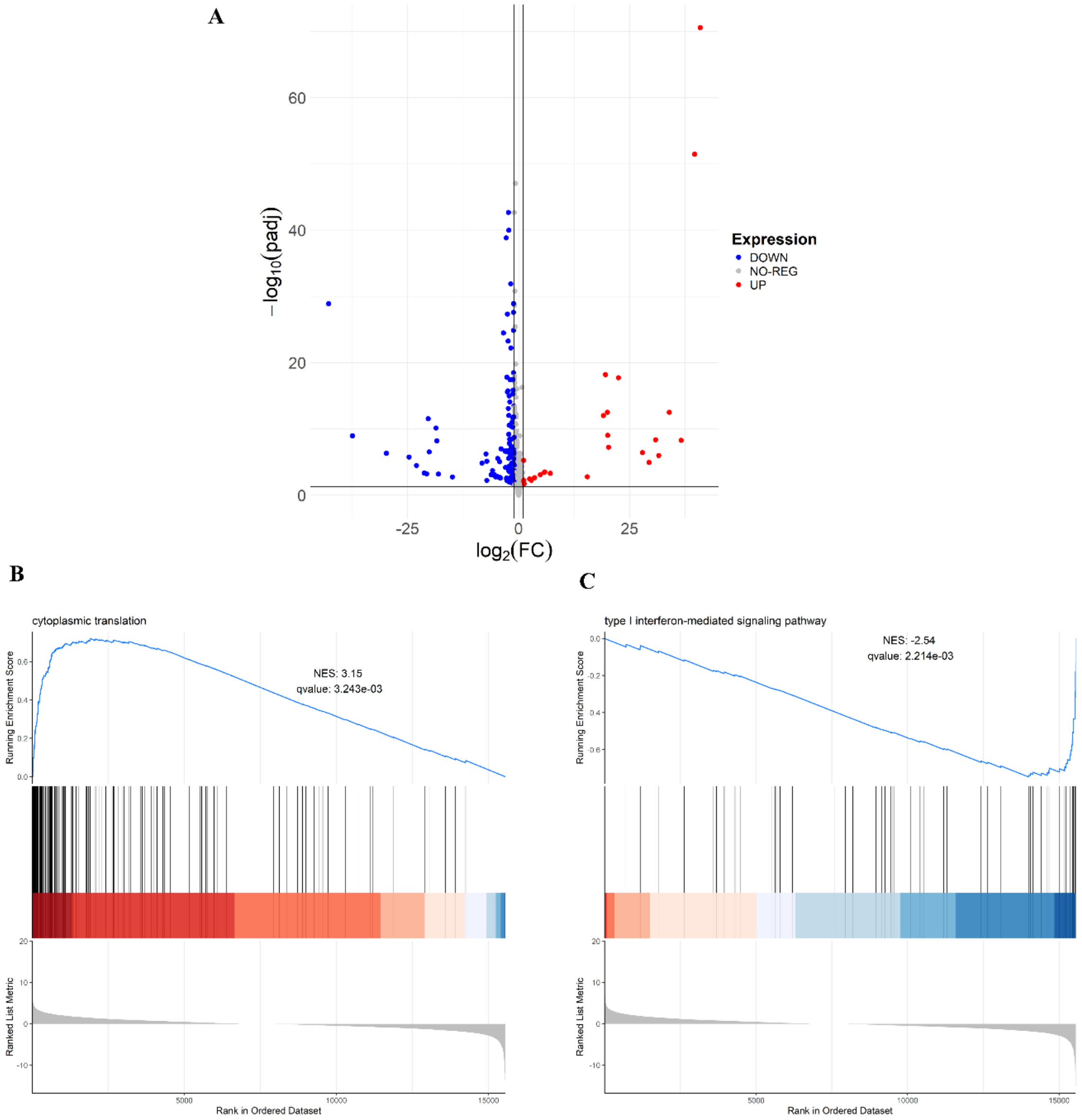

Supplementary figure S2. Transcriptomic analysis of p65 iso5 versus p65. (**A)** Volcano plot of DEGs highlighting upregulated (red) and downregulated (blue) transcripts following Treatment. **(B)** Cytoplasmic translation enrichment plot (NES = 3.15, q-value < 0.01)**. (C)** Type I interferon-mediated signaling pathway enrichment plot (NES=-2.54, q-value < 0.001). Abbreviations: NES, normalized enrichment score**.**

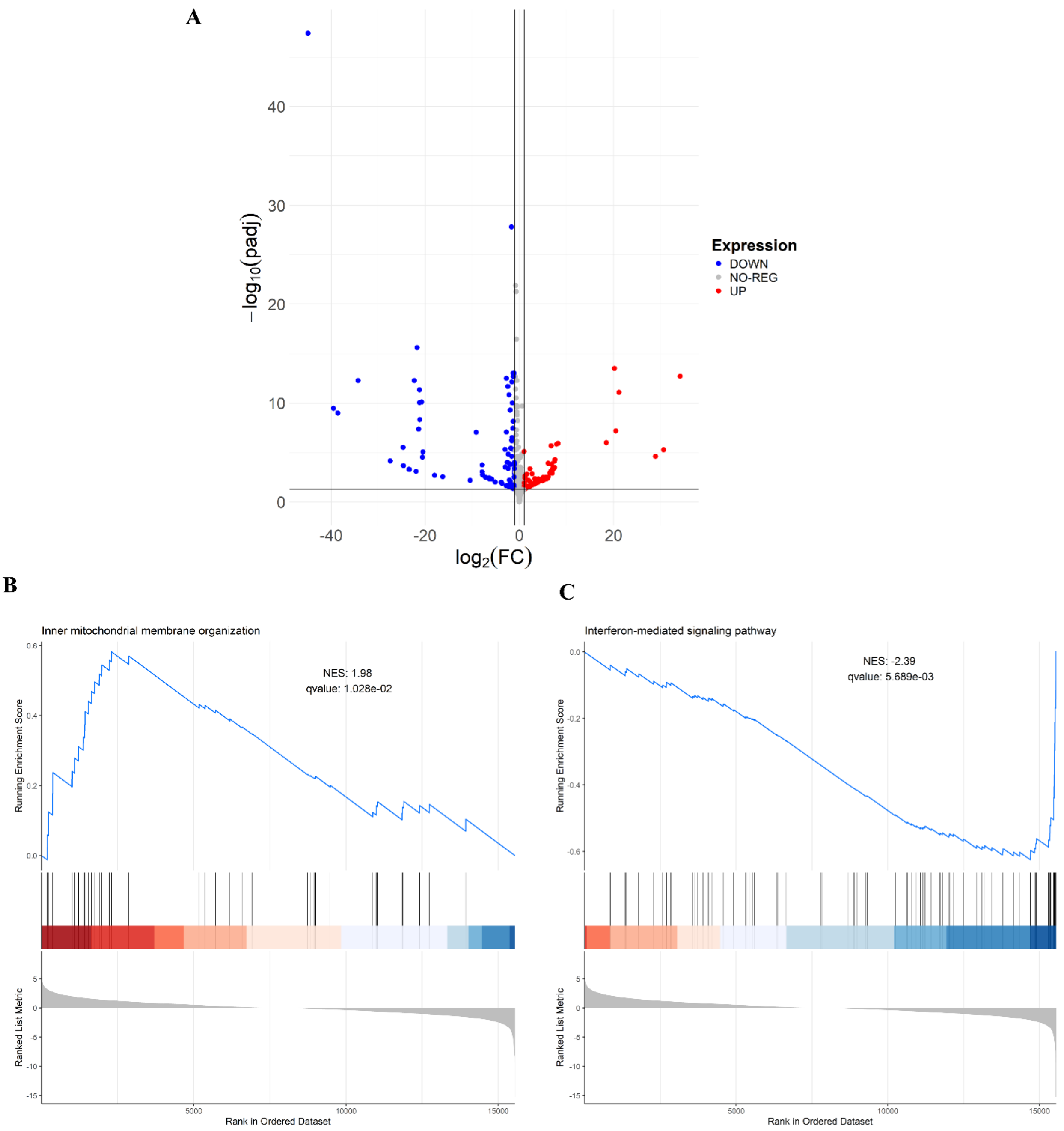

Supplementary figure S3. Transcriptomic analysis of p65 iso5 versus p65 following dexamethasone treatment. **(A)** Volcano plot of DEGs highlighting upregulated (red) and downregulated (blue) transcripts following Treatment. **(B)** Inner mitochondrial membrane organization enrichment plot (NES = 1.98, q-value < 0.01)**. (C)** Interferon-mediated signaling pathway enrichment plot (NES= -2.39, q-value < 0.001). Abbreviations: NES, normalized enrichment score.

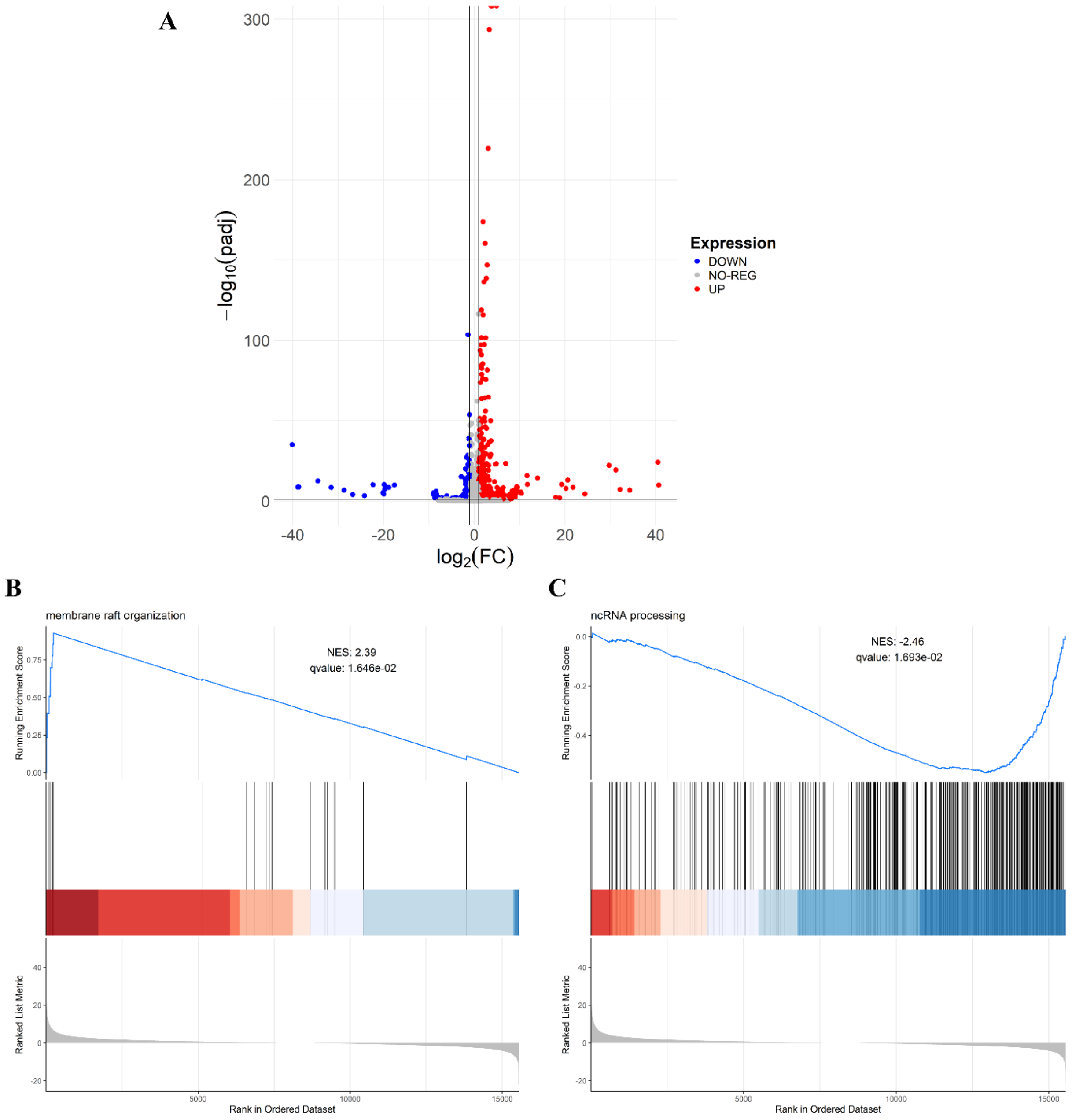

Supplementary figure S4. Transcriptomic analysis of p65 iso5 cells following dexamethasone treatment compared with untreated p65 iso5 cells. **(A)** Volcano plot of DEGs highlighting upregulated (red) and downregulated (blue) transcripts following Treatment. **(B)** Membrane raft organization enrichment plot (NES = 2.39 q-value < 0.01)**.** **(C)** ncRNA processing enrichment plot (NES= -2.46, q-value < 0.001). Abbreviations: NES, normalized enrichment score.
