# Supplementary material for: The NF-κB Isoform p65 iso5 Is Associated with Distinct Transcriptional Programs and Signaling Pathways
Source: Cells. 2026 Sep 10;15(18):1643. doi: 10.3390/cells15181643 (PMC13605157; doi:10.3390/cells15181643)
Supplement: Supplementary file 1 [file cells-15-01643-s001.zip › TABLE S1.pdf]

baseMean	log2FoldChange	lfcSE	pvalue	padj	Symbol	ENTREZ	delabel	diffexp	UNIQUE
17046,92164	-1,16462393	0,108221229	1,52811E-28	1,3413E-25	ISG15	9636	ISG15	DOWN	0
8175,946722	-3,438989817	0,32647277	4,03198E-28	3,33089E-25	IFI6	2537	IFI6	DOWN	0
6647,538192	-1,166577168	0,102859199	2,34711E-31	2,5356E-28	STAT1	6772	STAT1	DOWN	1
5550,196915	-1,058006676	0,133145073	9,66441E-17	3,31042E-14	ZC3HAV1	56829	ZC3HAV1	DOWN	1
5593,640197	-1,737900236	0,170462035	8,12304E-26	6,00421E-23	SDC4	6385	SDC4	DOWN	0
5072,687511	-2,298603758	0,161678	7,16645E-47	2,14336E-43	OAS3	4940	OAS3	DOWN	0
4201,654603	-1,145256472	0,123270296	5,02304E-22	3,35922E-19	GBP1	2633	GBP1	DOWN	1
4105,675013	-1,163926871	0,100092103	8,54654E-33	1,20028E-29	TAP1	6890	TAP1	DOWN	1
4069,80911	-1,799671678	0,146760378	7,38075E-36	1,29569E-32	C3	718	C3	DOWN	0
3986,710852	-1,30042372	0,155863998	1,44099E-18	5,46953E-16	MUC1	4582	MUC1	DOWN	0
3651,844111	-1,25803058	0,147543417	3,29321E-19	1,40151E-16	ZNFX1	57169	ZNFX1	DOWN	1
3564,568083	-1,018883156	0,16018995	9,82458E-12	1,94333E-09	PLXNA2	5362	PLXNA2	DOWN	0
3094,812895	-1,279699566	0,175440574	6,52088E-15	1,72791E-12	SAMD9	54809	SAMD9	DOWN	1
2534,459448	-2,01558043	0,322417285	1,9313E-11	3,71551E-09	RIGI	23586	RIGI	DOWN	0
2663,193557	-1,285795883	0,14343476	6,27637E-21	3,39021E-18	TNFAIP3	7128	TNFAIP3	DOWN	0
2459,284477	-1,075157222	0,145124269	5,8126E-15	1,57476E-12	PML	5371	PML	DOWN	1
2115,431028	-1,774500702	0,25630534	1,5081E-13	3,47209E-11	IFI44L	10964	IFI44L	DOWN	0
2073,865729	-1,076426212	0,193871238	9,07466E-10	1,43196E-07	IGFBP6	3489	IGFBP6	DOWN	1
1729,158131	-1,513951658	0,215175438	4,03967E-14	1,01309E-11	CSF1	1435	CSF1	DOWN	1
1744,152499	-2,80883059	0,208227334	7,25786E-43	1,45613E-39	HELZ2	85441	HELZ2	DOWN	0
1705,279392	-2,234383976	0,162384023	4,50528E-44	1,05453E-40	OAS1	4938	OAS1	DOWN	0
1614,277128	-1,320800374	0,182516585	9,28587E-15	2,41501E-12	PLSCR1	5359	PLSCR1	DOWN	1
1578,530485	-2,634444797	0,287407568	2,72232E-21	1,59301E-18	IFIT3	3437	IFIT3	DOWN	0
1484,392368	-1,131927542	0,222931469	8,76299E-09	1,07015E-06	DDX60	55601	DDX60	DOWN	0
1453,475319	-4,278376444	0,992274218	9,37812E-08	9,08319E-06	MX1	4599	MX1	DOWN	1
1425,327817	-1,053886343	0,199201686	3,51921E-09	4,75228E-07	CD83	9308	CD83	DOWN	1
1424,619332	-3,968175272	0,744350457	6,30949E-10	1,00694E-07	IFI27	3429	IFI27	DOWN	0
1447,853821	-1,091326271	0,205665439	3,10067E-09	4,31147E-07	MYO1G	64005	MYO1G	DOWN	0
1434,053897	-1,383077846	0,283979827	1,84837E-08	2,09343E-06	PARP12	64761	PARP12	DOWN	0
1336,769335	-1,592030379	0,365546698	1,77001E-07	1,59346E-05	OASL	8638	OASL	DOWN	0
1288,167573	-2,518204594	0,221572331	4,61065E-31	4,62515E-28	IFIT1	3434	IFIT1	DOWN	1
1312,164333	-1,344606296	0,198470504	2,48721E-13	5,63393E-11	SLFN5	162394	SLFN5	DOWN	1

baseMean	log2FoldChange	lfcSE	pvalue	padj	Symbol	ENTREZ	delabel	diffexp	UNIQUE
1256,843258	-2,540651653	0,2967462	7,04474E-19	2,82675E-16	IFIT2	3433	IFIT2	DOWN	0
1210,392007	-1,84652334	0,333245718	9,47484E-10	1,4785E-07	PDZD2	23037	PDZD2	DOWN	1
1189,271168	1,124504365	0,241663447	5,48896E-08	5,50621E-06	EDN2	1907	EDN2	UP	0
1138,058844	-1,933276139	0,213341245	7,80477E-21	3,91465E-18	C1S	716	C1S	DOWN	0
1084,741793	-1,323242568	0,252414039	3,0488E-09	4,28174E-07	HERC5	51191	HERC5	DOWN	1
1059,702371	-1,682955246	0,272559294	1,74319E-11	3,40019E-09	C1QTNF1	114897	C1QTNF1	DOWN	0
1063,3808	-1,974598179	0,242734516	2,48073E-17	8,70984E-15	DTX3L	151636	DTX3L	DOWN	0
1019,000494	-2,119020213	0,251564991	2,74004E-18	1,01266E-15	PARP10	84875	PARP10	DOWN	0
1003,691448	-1,016843917	0,247179625	3,61436E-07	3,02143E-05	IFI16	3428	IFI16	DOWN	1
984,231272	-2,342515361	0,297736442	2,63198E-16	8,59618E-14	OLR1	4973	OLR1	DOWN	0
1039,617051	-2,141939442	0,304234038	1,20951E-13	2,83105E-11	ICAM1	3383	ICAM1	DOWN	0
983,1154549	-1,182879124	0,218412496	1,50185E-09	2,26795E-07	TDRD7	23424	TDRD7	DOWN	1
910,1530341	-1,227738613	0,278987103	1,37766E-07	1,28132E-05	MAFF	23764	MAFF	DOWN	0
959,4912201	-2,26919032	0,65511049	2,71333E-06	0,00019115	PI3	5266	PI3	DOWN	0
885,974118	-1,012452857	0,298435583	2,76674E-06	0,00019115	LAMA3	3909	LAMA3	DOWN	1
861,2932834	-1,072987618	0,223168043	3,0099E-08	3,17827E-06	TRIM21	6737	TRIM21	DOWN	1
848,2194362	-2,391063354	0,227634959	7,01078E-27	5,46997E-24	PARP14	54625	PARP14	DOWN	0
820,4796917	-2,211554051	0,293277242	3,30464E-15	9,47151E-13	PARP9	83666	PARP9	DOWN	0
846,0935728	-1,965418951	0,370792987	3,94917E-09	5,18337E-07	HERC6	55008	HERC6	DOWN	0
810,5199913	-1,041621141	0,387738291	1,48253E-05	0,000788663	ENPP1	5167	ENPP1	DOWN	0
756,242468	-1,643485243	0,287802057	2,65086E-10	4,43198E-08	HLA-F	3134	HLA-F	DOWN	0
750,3555929	-2,401706706	0,278140503	4,43063E-19	1,83011E-16	GBP3	2635	GBP3	DOWN	0
706,8798355	-1,532398317	0,457765863	3,41911E-06	0,000229751	GBP2	2634	GBP2	DOWN	0
674,5198232	-1,618239418	0,313142153	4,91805E-09	6,22245E-07	IFI35	3430	IFI35	DOWN	1
653,9771339	-1,394305602	0,429833437	4,24849E-06	0,000280121	SP110	3431	SP110	DOWN	1
546,0829927	-2,546466663	0,460232486	1,23315E-09	1,90311E-07	APOL1	8542	APOL1	DOWN	0
560,1374217	-1,941146771	0,364179607	3,29909E-09	4,49829E-07	C1R	715	C1R	DOWN	0
465,9722514	-2,097976237	0,348667335	8,70104E-11	1,50861E-08	TRANK1	9881	TRANK1	DOWN	1
432,8135383	-2,80293577	0,519937406	1,83406E-09	2,74017E-07	IFIH1	64135	IFIH1	DOWN	0
440,2091206	-2,151880612	0,432043756	2,12889E-08	2,37287E-06	SAMD9L	219285	SAMD9L	DOWN	1
435,7547721	-1,390424178	0,390559841	1,9408E-06	0,000141226	CSF2RA	1438	CSF2RA	DOWN	0
433,6428966	-1,077359685	0,670364652	0,000141571	0,005164206	IRAK2	3656	IRAK2	DOWN	0

baseMean	log2FoldChange	lfcSE	pvalue	padj	Symbol	ENTREZ	delabel	diffexp	UNIQUE
381,5642464	-2,006512313	0,514356022	9,79363E-07	7,51594E-05	APOL6	80830	APOL6	DOWN	0
378,0958009	-1,755238243	0,851929077	4,13866E-05	0,001881014	CXCL8	3576	CXCL8	DOWN	0
400,9324812	-2,265510545	0,459479561	2,72668E-08	2,94565E-06	DHRS2	10202	DHRS2	DOWN	0
385,638537	-1,478775434	0,841571116	8,15588E-05	0,00325401	NECTIN4	81607	NECTIN4	DOWN	0
350,9559315	-2,22577223	0,339067365	3,13308E-12	6,58243E-10	PSMB9	5698	PSMB9	DOWN	0
329,3114458	-3,085782258	0,57813652	1,47662E-09	2,25409E-07	IFITM1	8519	IFITM1	DOWN	0
281,2248698	-1,910817895	2,296937111	0,00022755	0,007663583	RSAD2	91543	RSAD2	DOWN	0
304,637827	-1,287014726	0,647975737	5,86906E-05	0,002520644	USH1C	10083	USH1C	DOWN	0
313,4969456	-1,672070859	0,849679302	5,05897E-05	0,002234221	DHX58	79132	DHX58	DOWN	0
281,5135737	-2,944892351	0,775094529	8,25794E-07	6,47902E-05	IRF7	3665	IRF7	DOWN	1
307,8658268	-7,349416748	1,388526982	5,03159E-09	6,30926E-07	ABCC8	6833	ABCC8	DOWN	0
267,9979077	-1,367631118	0,511471289	1,46812E-05	0,000788663	ARID3B	10620	ARID3B	DOWN	1
234,0432115	-1,263846667	1,082262704	0,000268304	0,008722369	IGFL1	374918	IGFL1	DOWN	1
242,3989861	-1,70370815	1,783955293	0,000220331	0,007565606	CCL20	6364	CCL20	DOWN	1
218,9799328	-2,046166331	0,632187375	4,57767E-06	0,000297633	USP18	11274	USP18	DOWN	1
198,4878527	-2,126996234	0,516972422	5,45899E-07	4,38092E-05	PLEKHA4	57664	PLEKHA4	DOWN	0
201,9510159	40,90551798	2,237138364	1,87982E-75	2,64002E-71	RBBP8NL	140893	RBBP8NL	UP	1
173,159619	-1,054069852	0,79978773	0,00025785	0,008460838	MTSS1	9788	MTSS1	DOWN	1
162,9707336	1,155780049	0,776299006	0,000168291	0,005983486	SLC2A11	66035	SLC2A11	UP	1
150,6130166	-4,719354755	1,012148838	2,81001E-08	2,98968E-06	UBE2L6	9246	UBE2L6	DOWN	1
169,2449066	-1,247404355	1,335235683	0,000371572	0,011116972	SHROOM2	357	SHROOM2	DOWN	0
143,1440221	-1,584144375	0,749557841	4,09017E-05	0,001871086	BRSK1	84446	BRSK1	DOWN	0
145,323452	-1,250378093	1,125890359	0,000296698	0,00938365	SHFL	55337	SHFL	DOWN	1
152,2816475	-1,149100884	0,88970535	0,000246255	0,008175891	WNT11	7481	WNT11	DOWN	0
135,8900428	-1,10110026	1,07687249	0,000408268	0,011920397	RALGPS1	9649	RALGPS1	DOWN	0
130,6293577	-1,2038452	1,556892312	0,00046334	0,013040381	RRAD	6236	RRAD	DOWN	0
105,5781736	-1,410225997	1,262198111	0,000243257	0,008095517	TNFRSF1B	7133	TNFRSF1B	DOWN	1
97,94236887	-1,194773643	2,433421867	0,000671347	0,017395565	SLC15A3	51296	SLC15A3	DOWN	0
108,8198448	-2,032928105	1,435586682	9,01123E-05	0,003505645	SGPP2	130367	SGPP2	DOWN	0
109,4565327	2,465981099	2,031291755	9,19474E-05	0,003567154	TMEM266	123591	TMEM266	UP	1
98,27068281	-2,706796054	1,924122921	6,08232E-05	0,002565167	GCNT3	9245	GCNT3	DOWN	0
91,55394373	1,126598425	1,922424567	0,000627197	0,016619541	SLC38A4	55089	SLC38A4	UP	0

baseMean	log2FoldChange	lfcSE	pvalue	padj	Symbol	ENTREZ	delabel	diffexp	UNIQUE
80,58793672	5,902956715	2,23628801	5,06642E-06	0,000324898	CLMN	79789	CLMN	UP	1
73,65356881	-1,680464039	1,75982927	0,000224828	0,007647868	CASP1	834	CASP1	DOWN	0
73,32527032	-1,138773737	1,703539467	0,000560798	0,015233739	LRRC32	2615	LRRC32	DOWN	1
71,06554265	1,050131158	1,271571876	0,00055537	0,015198446	MAGED1	9500	MAGED1	UP	1
66,40302087	3,584255058	2,682712394	5,18555E-05	0,002267752	INHA	3623	INHA	UP	0
70,60056217	5,754558178	2,280004588	6,07786E-06	0,000373582	SLC6A19	340024	SLC6A19	UP	1
57,68112998	-2,724392412	2,404138457	9,39553E-05	0,003635009	IL1B	3553	IL1B	DOWN	0
46,84822135	-5,440753564	2,90891883	1,6315E-05	0,000854955	FBLN7	129804	FBLN7	DOWN	0
50,71680315	-1,088758683	2,073384837	0,000711179	0,018225916	TRIM10	10107	TRIM10	DOWN	0
46,50705614	-8,239639219	1,884165461	1,59853E-07	1,45777E-05	TTLL6	284076	TTLL6	DOWN	0
48,21009662	-5,874257667	1,935308397	2,7766E-06	0,00019115	TBX4	9496	TBX4	DOWN	0
42,1580992	-7,175785182	1,547535028	7,90182E-08	7,76037E-06	PTP4A1	7803	PTP4A1	DOWN	0
42,48758315	4,876243441	2,491882699	1,5431E-05	0,000817787	LRRIQ3	127255	LRRIQ3	UP	1
39,54184187	-6,210127267	3,425188291	1,61184E-05	0,000849564	LCN2	3934	LCN2	DOWN	0
35,91463676	-2,118334291	3,017271981	0,000253491	0,008356881	COL8A2	1296	COL8A2	DOWN	0
34,44312769	-4,210673951	3,240903345	4,81393E-05	0,00214625	ZNF711	7552	ZNF711	DOWN	0
34,69957117	-2,246481364	2,901383733	0,000210703	0,007252721	PRKG2	5593	PRKG2	DOWN	0
35,29983288	19,57906185	2,054798596	9,91363E-22	6,3285E-19	KIF17	57576	KIF17	UP	1
36,32404751	-5,20767344	3,775003524	3,56168E-05	0,001672918	ETV7	51513	ETV7	DOWN	0
32,36316487	-1,200765445	2,400589206	0,000657628	0,017198747	TPPP3	51673	TPPP3	DOWN	0
31,43135712	-4,569945691	3,375008899	4,11719E-05	0,001877331	GPR17	2840	GPR17	DOWN	0
33,86058189	-5,485355289	3,064482333	1,82639E-05	0,000939217	FIBIN	387758	FIBIN	DOWN	0
29,43797016	-4,806178939	3,270525078	3,29217E-05	0,001577179	PTGFR	5737	PTGFR	DOWN	0
30,63036633	-2,595360489	3,361889015	0,000183455	0,006489795	BIK	638	BIK	DOWN	0
28,80672944	22,49205955	2,394433422	3,35227E-21	1,88317E-18	MAPT	4137	MAPT	UP	1
30,53357264	7,128737164	3,275095662	8,70625E-06	0,000513742	ZNF608	57507	ZNF608	UP	1
23,09380312	-5,128483247	3,689926064	3,54498E-05	0,001670663	SCUBE3	222663	SCUBE3	DOWN	1
22,07963379	2,838591306	3,491506195	0,000156723	0,005643628	STKLD1	169436	STKLD1	UP	1
22,39388256	-4,142275418	3,513170025	5,94519E-05	0,002537817	ZBED6	100381270	ZBED6	DOWN	0
21,26330431	-2,122144878	3,507314211	0,000303634	0,009539664	LGALS9	3965	LGALS9	DOWN	1
20,47128759	-1,213028098	2,511266018	0,000668981	0,017366298	GALNT4	8693	GALNT4	DOWN	1
21,15042613	-20,37904626	2,662144185	1,12042E-14	2,86095E-12	TMEM125	128218	TMEM125	DOWN	0

baseMean	log2FoldChange	lfcSE	pvalue	padj	Symbol	ENTREZ	delabel	diffexp	UNIQUE
21,15500439	19,98213305	2,512280393	1,06363E-15	3,17822E-13	VWA5B2	90113	VWA5B2	UP	1
20,7680557	39,68962998	2,533141429	4,72369E-56	3,31698E-52	KDF1	126695	KDF1	UP	1
18,0241042	-1,725834202	3,314307474	0,000437518	0,012514274	ANGPTL2	23452	ANGPTL2	DOWN	1
21,406713	1,045144972	2,597835117	0,000915439	0,021976802	KCNIP2	30819	KCNIP2	UP	1
21,17119329	-1,48751964	2,885293328	0,000508435	0,014083756	TMPRSS13	84000	TMPRSS13	DOWN	0
17,63575197	-18,0728798	8,806184022	1,16375E-05	0,000640932	SFTPA2	729238	SFTPA2	DOWN	0
15,87655001	-1,91880152	2,97947104	0,00031042	0,009709441	SMIM22	440335	SMIM22	DOWN	1
15,18824535	-1,863930651	3,032081069	0,000336828	0,010328411	CEMIP	57214	CEMIP	DOWN	1
15,78960213	-18,61603722	2,588945687	3,34233E-13	7,45073E-11	OVCH1	341350	OVCH1	DOWN	0
18,95116238	19,11956648	2,452173515	3,60474E-15	1,0125E-12	FAM186B	84070	FAM186B	UP	1
18,34001351	-18,43294311	2,832832297	3,49531E-11	6,29335E-09	TACR2	6865	TACR2	DOWN	0
10,62886467	-37,46192833	5,620088342	5,34985E-12	1,08889E-09	CILP	8483	CILP	DOWN	0
12,06454193	20,2703423	3,291565644	3,58901E-10	5,86093E-08	TRIM17	51127	TRIM17	UP	1
12,71930003	1,237080436	2,924843029	0,000743366	0,018878532	SPDYE5	442590	SPDYE5	UP	0
12,20996269	-7,176899958	10,11294771	0,000168083	0,005983486	RAB33A	9363	RAB33A	DOWN	0
8,272648476	-20,71076114	9,91815646	1,09043E-05	0,000607696	CLDN19	149461	CLDN19	DOWN	0
10,92104291	20,09379774	2,942637473	4,62492E-12	9,55182E-10	EYA2	2139	EYA2	UP	0
7,770862314	-42,81610601	3,644801765	1,01593E-32	1,29706E-29	TNFSF14	8740	TNFSF14	DOWN	0
10,2076927	-29,8072772	5,212136215	3,58925E-09	4,77949E-07	ODAD1	93233	ODAD1	DOWN	0
10,005121	-20,13811894	3,434846761	2,01823E-09	2,95251E-07	C17orf107	100130311	C17orf107	DOWN	0
6,556887343	-24,69299511	4,510140014	1,60494E-08	1,8325E-06	BATF	10538	BATF	DOWN	0
9,825435976	31,57998977	5,716762916	8,95705E-09	1,08442E-06	MBOAT4	619373	MBOAT4	UP	0
5,873566474	-23,02174159	5,16216836	4,06843E-07	3,34135E-05	CACNA1B	774	CACNA1B	DOWN	1
6,177173093	33,94352303	4,316687115	1,0563E-15	3,17822E-13	NR2E3	10002	NR2E3	UP	0
9,5133045	36,62335791	5,701106888	2,87463E-11	5,31202E-09	TMEM204	79652	TMEM204	UP	0
8,13187484	29,38910126	5,994139827	1,20154E-07	1,14017E-05	SOWAHD	347454	SOWAHD	UP	0
6,201577351	27,89413658	4,814000991	2,63384E-09	3,81336E-07	PIK3R2	5296	PIK3R2	UP	0
5,477506433	30,86625965	4,73516522	2,44308E-11	4,57476E-09	ANGPTL8	55908	ANGPTL8	UP	0
6,386418515	-21,25683975	8,997275658	7,62853E-06	0,000455894	SPMIP3	200159	SPMIP3	DOWN	0
5,213978543	-14,9367881	11,59238911	3,93358E-05	0,00182321	KRT1	3848	KRT1	DOWN	0
3,91861701	15,48212065	11,51631277	3,53556E-05	0,001670663	NLRP12	91662	NLRP12	UP	0
