# Supplementary material for: The NF-κB Isoform p65 iso5 Is Associated with Distinct Transcriptional Programs and Signaling Pathways
Source: Cells. 2026 Sep 10;15(18):1643. doi: 10.3390/cells15181643 (PMC13605157; doi:10.3390/cells15181643)
Supplement: Supplementary file 1 [file cells-15-01643-s001.zip › TABLE S2.pdf]

baseMean	log2FoldChange	lfcSE	pvalue	padj	Symbol	ENTREZ	delabel	diffexp	UNIQUE
8175,946722	-2,45957074	0,335170929	2,62827E-15	2,09473E-12	IFI6	2537	IFI6	DOWN	0
5072,687511	-1,307435463	0,167942633	5,885E-17	9,38069E-14	OAS3	4940	OAS3	DOWN	0
4069,80911	-1,686476692	0,145634868	2,07713E-32	1,48993E-28	C3	718	C3	DOWN	0
2534,459448	-1,080311491	0,424939523	1,18025E-05	0,001302453	RIGI	23586	RIGI	DOWN	0
2663,193557	-1,129481943	0,147128502	5,44219E-17	9,38069E-14	TNFAIP3	7128	TNFAIP3	DOWN	0
2271,282454	-1,055333237	0,284670149	5,90413E-07	9,96478E-05	IFI44	10561	IFI44	DOWN	0
1744,152499	-1,309427255	0,220181367	1,84438E-11	6,96303E-09	HELZ2	85441	HELZ2	DOWN	0
1705,279392	-1,300707959	0,17025328	1,73381E-16	2,07277E-13	OAS1	4938	OAS1	DOWN	0
1578,530485	-1,953004267	0,299049423	1,15551E-12	5,18029E-10	IFIT3	3437	IFIT3	DOWN	0
1484,392368	-1,518658783	0,223026624	1,77241E-13	9,7796E-11	DDX60	55601	DDX60	DOWN	0
1424,619332	-2,998897549	0,882294605	2,06229E-06	0,000301894	IFI27	3429	IFI27	DOWN	0
1434,053897	-1,000752176	0,330635346	4,22581E-06	0,000531785	PARP12	64761	PARP12	DOWN	0
1336,769335	-1,607781483	0,376538312	1,2144E-07	2,41969E-05	OASL	8638	OASL	DOWN	0
1256,843258	-1,659672057	0,315678848	1,87788E-09	5,73192E-07	IFIT2	3433	IFIT2	DOWN	0
1138,058844	-1,633436878	0,215305914	8,82491E-16	7,44719E-13	C1S	716	C1S	DOWN	0
1059,702371	-1,097088629	0,310336908	9,85116E-07	0,000157028	C1QTNF1	114897	C1QTNF1	DOWN	0
1063,3808	-1,005540868	0,279657616	8,86446E-07	0,000145695	DTX3L	151636	DTX3L	DOWN	0
992,7601302	1,010966155	0,227145699	3,67185E-08	7,98126E-06	ELFN2	114794	ELFN2	UP	1
1019,000494	-1,271488373	0,277083046	2,11553E-08	4,89507E-06	PARP10	84875	PARP10	DOWN	0
984,231272	-2,203686	0,312159669	2,19816E-14	1,4334E-11	OLR1	4973	OLR1	DOWN	0
1039,617051	-1,078653338	0,386798556	6,89427E-06	0,000815407	ICAM1	3383	ICAM1	DOWN	0
959,4912201	-3,039635722	0,618498954	2,00992E-08	4,80573E-06	PI3	5266	PI3	DOWN	0
848,2194362	-1,384241804	0,242276791	9,47745E-11	3,48624E-08	PARP14	54625	PARP14	DOWN	0
820,4796917	-1,624082782	0,311198473	2,19477E-09	6,55962E-07	PARP9	83666	PARP9	DOWN	0
846,0935728	-1,029040904	0,526537763	4,03375E-05	0,003662542	HERC6	55008	HERC6	DOWN	0
756,242468	-1,57105586	0,293153705	1,01559E-09	3,16733E-07	HLA-F	3134	HLA-F	DOWN	0
750,3555929	-1,062792617	0,339690453	3,0887E-06	0,000418024	GBP3	2635	GBP3	DOWN	0
706,8798355	-1,12418864	0,561876567	3,3485E-05	0,003160367	GBP2	2634	GBP2	DOWN	0
546,0829927	-2,775588973	0,48985833	2,58273E-10	8,61671E-08	APOL1	8542	APOL1	DOWN	0
560,1374217	-1,826416282	0,378608341	1,48924E-08	3,62111E-06	C1R	715	C1R	DOWN	0
410,8058809	1,199279846	0,564345286	2,48263E-05	0,002422842	TMEM67	91147	TMEM67	UP	1
381,5642464	-2,024741717	0,559956547	8,93711E-07	0,000145695	APOL6	80830	APOL6	DOWN	0

baseMean	log2FoldChange	lfcSE	pvalue	padj	Symbol	ENTREZ	delabel	diffexp	UNIQUE
350,9559315	-2,741888194	0,357076365	2,80962E-16	3,10052E-13	PSMB9	5698	PSMB9	DOWN	0
329,3114458	-2,567408353	0,666557008	5,49606E-07	9,44937E-05	IFITM1	8519	IFITM1	DOWN	0
280,5623566	1,175383479	0,62794905	4,06746E-05	0,003669923	TBCE	6905	TBCE	UP	1
246,3610227	1,592692159	0,692356434	1,43225E-05	0,001543322	AP4S1	11154	AP4S1	UP	1
245,8702334	2,256693129	0,746125045	3,3231E-06	0,000441418	MEF2C	4208	MEF2C	UP	1
234,6135157	-2,392971527	3,646540016	0,00057828	0,026504813	CXCL10	3627	CXCL10	DOWN	1
214,0777986	-1,8402753	0,517808588	1,05354E-06	0,00016609	APOL3	80833	APOL3	DOWN	1
198,4878527	-2,40163714	0,539175502	6,9105E-08	1,45791E-05	PLEKHA4	57664	PLEKHA4	DOWN	0
174,3426529	1,042820047	0,964917571	0,000195906	0,012435712	BBS5	129880	BBS5	UP	1
164,1427901	-1,940576732	1,691356878	0,000125091	0,008796808	CXCL1	2919	CXCL1	DOWN	0
115,727894	-7,831586731	3,312681464	1,75964E-05	0,001842616	MX2	4600	MX2	DOWN	0
112,2839559	2,684010934	1,166605716	1,34768E-05	0,001464678	NPIPB5	100132247	NPIPB5	UP	1
106,3416534	6,730665746	1,197362424	7,96478E-09	2,04041E-06	DEXI	28955	DEXI	UP	1
102,4410754	7,903894992	1,4036915	5,6045E-09	1,46186E-06	CLDN3	1365	CLDN3	UP	1
95,48246953	3,284511458	1,993758069	5,04165E-05	0,004378463	EPCAM	4072	EPCAM	UP	1
104,1537119	2,181982834	1,82827189	0,000114182	0,008172522	FOXS1	2307	FOXS1	UP	1
97,94236887	-7,226880583	3,854193238	3,53888E-05	0,003296671	SLC15A3	51296	SLC15A3	DOWN	0
108,8198448	-2,127918821	1,591328909	8,02694E-05	0,006224565	SGPP2	130367	SGPP2	DOWN	0
96,08924508	-9,18283075	1,516359065	2,74697E-10	8,95637E-08	PLAAT4	5920	PLAAT4	DOWN	0
89,1845088	6,84935062	1,567216289	9,15224E-07	0,000147526	NYAP2	57624	NYAP2	UP	0
90,01096553	-2,37632052	0,776789001	3,15936E-06	0,000423591	CCDC69	26112	CCDC69	DOWN	1
78,23731022	7,524949577	1,573168971	2,80844E-07	5,23245E-05	UFSP1	402682	UFSP1	UP	1
81,36990702	1,878409492	1,426182865	8,32815E-05	0,006423418	RANGRF	29098	RANGRF	UP	1
76,01041296	3,345943097	2,974692243	0,000157845	0,010532275	PDE1C	5137	PDE1C	UP	1
65,39609452	3,65700433	3,220328807	0,000155191	0,010413951	AFAP1L2	84632	AFAP1L2	UP	1
63,30419741	5,031994538	2,601515623	3,70899E-05	0,003410848	LUZP2	338645	LUZP2	UP	1
65,28866392	-1,204227609	1,557875806	0,000375888	0,020196609	DYSF	8291	DYSF	DOWN	0
61,99605561	-7,899181788	1,88483642	1,20858E-06	0,000184679	CBLN2	147381	CBLN2	DOWN	0
57,40800475	3,016559026	3,011536393	0,000212232	0,013295568	PZP	5858	PZP	UP	1
58,54331168	6,099800483	1,365625853	7,39096E-07	0,000123292	ZNF169	169841	ZNF169	UP	1
56,60857213	1,322264708	1,883820965	0,000487334	0,023973935	FGF22	27006	FGF22	UP	1
54,17238116	8,211499694	1,45244082	4,44169E-09	1,18001E-06	C5	727	C5	UP	1

baseMean	log2FoldChange	lfcSE	pvalue	padj	Symbol	ENTREZ	delabel	diffexp	UNIQUE
57,68112998	-1,138449019	1,907149402	0,000755425	0,03110651	IL1B	3553	IL1B	DOWN	0
51,96792175	1,074932241	1,55352781	0,000508211	0,024302668	CAMK2N1	55450	CAMK2N1	UP	0
58,04567185	-5,133233623	4,668020331	0,000144166	0,009991345	SLC6A12	6539	SLC6A12	DOWN	1
48,66653793	5,587801454	2,799973437	3,39756E-05	0,003185716	KLHL1	57626	KLHL1	UP	1
49,0466498	7,467870113	1,600611451	4,06081E-07	7,19215E-05	KCTD16	57528	KCTD16	UP	1
48,31830453	7,08919978	1,942240474	3,36272E-06	0,000442584	SLC38A3	10991	SLC38A3	UP	1
46,84822135	7,253701166	2,017985147	3,61782E-06	0,000467579	FBLN7	129804	FBLN7	UP	0
44,22214014	3,997091576	2,466814048	5,87242E-05	0,004854539	CCDC149	91050	CCDC149	UP	1
45,31358239	1,021074306	1,849903148	0,000930468	0,036174782	CD163L1	283316	CD163L1	UP	0
40,02609931	7,48337532	1,926952064	2,24085E-06	0,000318289	PCDHA12	56137	PCDHA12	UP	1
43,12878816	6,857070759	2,0951702	5,8766E-06	0,000718505	RGS11	8786	RGS11	UP	1
40,81314131	3,617862237	3,353202889	0,000176582	0,011411001	LY6G5C	80741	LY6G5C	UP	1
46,16319373	-1,203170845	1,891554658	0,00063612	0,028242592	CARD16	114769	CARD16	DOWN	1
37,9225364	6,932932256	2,201909287	6,93431E-06	0,000815407	FKBP1B	2281	FKBP1B	UP	1
36,99058502	7,069426186	1,946892162	3,46519E-06	0,000451924	C3orf33	285315	C3orf33	UP	1
36,27091209	3,046151488	3,150635903	0,000232992	0,014163123	CAVIN4	347273	CAVIN4	UP	1
39,17542085	1,065083529	2,017745907	0,001055068	0,038444209	PROM2	150696	PROM2	UP	1
40,98305927	-2,227761399	3,114589312	0,000494145	0,023973935	TNFRSF9	3604	TNFRSF9	DOWN	1
41,06537528	4,590138104	3,337139094	9,43388E-05	0,006940433	KCNJ14	3770	KCNJ14	UP	1
35,45774511	3,342261926	3,175153516	0,000188052	0,012043721	SOBP	55084	SOBP	UP	1
36,06595517	-2,016863547	2,972106919	0,000564547	0,026122601	LYL1	4066	LYL1	DOWN	0
33,42970377	5,359605874	3,525015939	7,08931E-05	0,005557553	FILIP1	27145	FILIP1	UP	0
32,57683962	3,533516292	3,435612022	0,00019884	0,012547533	RIMS3	9783	RIMS3	UP	1
34,57331228	6,952926122	2,529912456	1,17246E-05	0,001302453	DLX5	1749	DLX5	UP	1
31,84205019	1,955897623	2,991828619	0,000618527	0,027557102	SLC10A4	201780	SLC10A4	UP	1
31,83336803	3,951900456	3,537872251	0,000160598	0,010666374	HOXC8	3224	HOXC8	UP	1
31,97527765	2,666814052	3,045751983	0,000298509	0,017061422	ASNS	440	ASNS	UP	1
35,16290531	-1,90240808	2,085484105	0,000245025	0,014707654	CHRDL2	25884	CHRDL2	DOWN	0
35,12890983	-3,870420684	3,350541698	0,000148427	0,010237185	GBGT1	26301	GBGT1	DOWN	0
30,50823507	5,710715788	3,518143924	5,88798E-05	0,004854539	C12orf56	115749	C12orf56	UP	1
27,22330924	1,607989611	2,387995159	0,000566299	0,026122601	TBC1D3	729873	TBC1D3	UP	1
27,16635454	6,461884047	2,271225383	1,05759E-05	0,001185329	C15orf62	643338	C15orf62	UP	0

baseMean	log2FoldChange	lfcSE	pvalue	padj	Symbol	ENTREZ	delabel	diffexp	UNIQUE
29,35370878	-3,703211627	3,852871708	0,000228699	0,01396133	TNFSF10	8743	TNFSF10	DOWN	0
28,04817592	4,702629664	3,41720314	9,38588E-05	0,006940433	PPP1R1B	84152	PPP1R1B	UP	0
26,13991214	4,929366626	3,666116673	9,80042E-05	0,007158355	ZNF528	84436	ZNF528	UP	1
23,86945222	1,134286662	2,415040699	0,001424667	0,046556446	ZNF490	57474	ZNF490	UP	1
25,40264036	-6,468795362	3,490024967	3,95856E-05	0,003617167	ADCY1	107	ADCY1	DOWN	0
31,11306129	-6,445910021	3,977793126	5,49862E-05	0,00464019	LCP1	3936	LCP1	DOWN	0
23,39869787	3,245360056	3,51963195	0,000260246	0,015491659	ERV3-1	2086	ERV3-1	UP	1
24,2245871	5,977233816	3,338789045	4,5059E-05	0,004015007	LAMA2	3908	LAMA2	UP	1
24,1457112	6,701045845	2,065500225	6,15114E-06	0,000741548	BBS1	582	BBS1	UP	1
22,15558559	1,788244812	3,023843837	0,000786695	0,031881158	ADGRV1	84059	ADGRV1	UP	1
25,05704189	6,026575591	3,333584965	4,37751E-05	0,003924983	AOC2	314	AOC2	UP	1
24,97645799	-5,88663306	3,74515556	6,26977E-05	0,005024919	SEPTIN4	5414	SEPTIN4	DOWN	1
25,35066198	-6,225086543	3,529331974	4,59001E-05	0,004064712	PPP1R1C	151242	PPP1R1C	DOWN	0
21,57476218	4,770358013	3,334733586	8,49733E-05	0,006463556	CLEC2L	154790	CLEC2L	UP	1
21,91993619	6,121910897	2,981976935	3,0775E-05	0,002923829	ZNF154	7710	ZNF154	UP	1
29,22688511	-7,902806689	2,661013573	8,09348E-06	0,000914245	H4C5	8367	H4C5	DOWN	0
21,1666522	1,168395397	2,63664498	0,001602503	0,049633155	ELL3	80237	ELL3	UP	0
19,9014331	2,704223786	3,362362175	0,000367208	0,019879101	LKAAEAR1	198437	LKAAEAR1	UP	1
19,79998785	1,250522838	2,369917134	0,001065201	0,038479746	LRRC7	57554	LRRC7	UP	1
19,93570823	2,190084576	3,343153373	0,000601706	0,027230539	ZNF493	284443	ZNF493	UP	1
20,44586183	-2,679782616	3,629222483	0,000441609	0,022419258	SLC10A5	347051	SLC10A5	DOWN	0
18,31113746	-20,48171951	4,217458121	3,96941E-08	8,49928E-06	SV2C	22987	SV2C	DOWN	0
21,17119329	-6,21129841	4,002083075	6,23481E-05	0,005024919	TMPRSS13	84000	TMPRSS13	DOWN	0
21,5272567	20,20502305	2,462067814	1,50577E-17	3,08597E-14	MASP2	10747	MASP2	UP	1
18,09977214	-1,345932111	2,783376428	0,001288688	0,043809271	RPGRIP1	57096	RPGRIP1	DOWN	1
17,68932167	1,763320847	3,02197752	0,000812257	0,032614571	CDH15	1013	CDH15	UP	0
18,05284323	2,039614487	3,396362401	0,000730567	0,030467177	CFAP54	144535	CFAP54	UP	1
16,43065122	-21,7442672	2,453251819	1,03895E-19	2,48413E-16	GRHL3	57822	GRHL3	DOWN	0
16,65219292	2,528229564	3,306599213	0,000416605	0,021798291	C1QL3	389941	C1QL3	UP	1
17,7580654	1,17041416	2,524919685	0,001459814	0,047464305	CDH3	1001	CDH3	UP	1
15,48539201	1,161067191	2,556523901	0,001527933	0,048631546	ZNF233	353355	ZNF233	UP	1
15,80876454	1,472518583	2,833305009	0,001084217	0,038982885	NDST3	9348	NDST3	UP	1

baseMean	log2FoldChange	lfcSE	pvalue	padj	Symbol	ENTREZ	delabel	diffexp	UNIQUE
16,22315686	-2,150954408	3,585772884	0,000712444	0,030076539	GAST	2520	GAST	DOWN	1
14,9384808	-20,58330564	4,587019256	1,55159E-07	3,00799E-05	SPACDR	402573	SPACDR	DOWN	0
14,83286419	21,13790223	2,858124216	1,204E-14	8,22501E-12	FNDC4	64838	FNDC4	UP	1
14,72474103	2,228024014	3,355642218	0,000582344	0,026521626	PLVAP	83483	PLVAP	UP	0
13,1884961	-20,79854476	2,952022564	1,30913E-13	7,82531E-11	RASGEF1A	221002	RASGEF1A	DOWN	0
13,69040481	1,819045944	3,13776562	0,00081616	0,032614571	TMEM191C	645426	TMEM191C	UP	1
18,34001351	-22,33010773	2,84016613	5,27444E-16	5,26088E-13	TACR2	6865	TACR2	DOWN	0
17,51296805	-2,797749654	4,010115642	0,000476817	0,023751424	NHLH2	4808	NHLH2	DOWN	1
16,01275974	-2,618821502	3,874937209	0,000523152	0,024687957	SLC6A4	6532	SLC6A4	DOWN	1
13,69409402	-1,450495675	2,949316665	0,001214284	0,042281858	CALHM6	441168	CALHM6	DOWN	1
14,30253528	-21,22975198	2,834505477	6,21368E-15	4,45707E-12	HLA-DQB1	3119	HLA-DQB1	DOWN	0
9,976984649	-21,16040559	3,312978752	1,19305E-11	4,7543E-09	SAMD7	344658	SAMD7	DOWN	0
13,42260465	-44,90567862	2,979005681	2,64775E-52	3,79846E-48	TMC8	147138	TMC8	DOWN	0
10,9914916	30,65168638	5,847970865	2,31317E-08	5,18512E-06	ARX	170302	ARX	UP	0
12,16212948	-38,60335526	5,717362344	2,42689E-12	1,024E-09	VANGL2	57216	VANGL2	DOWN	0
8,272648476	-23,46792192	8,24592222	3,88646E-06	0,000497814	CLDN19	149461	CLDN19	DOWN	0
10,92104291	-21,2390203	3,014270333	1,53045E-13	8,78233E-11	EYA2	2139	EYA2	DOWN	0
7,708854112	20,48122575	3,475079114	1,91876E-10	6,55394E-08	ONECUT1	3175	ONECUT1	UP	0
10,005121	-21,39598982	3,553970267	1,21334E-10	4,35166E-08	C17orf107	100130311	C17orf107	DOWN	0
6,556887343	-24,72067583	4,664054067	1,22287E-08	3,0247E-06	BATF	10538	BATF	DOWN	0
8,357574303	18,45846555	3,506948935	3,5954E-09	9,91915E-07	FDX2	112812	FDX2	UP	1
9,825435976	28,91898796	6,045817042	1,18473E-07	2,39383E-05	MBOAT4	619373	MBOAT4	UP	0
6,177173093	-34,27863874	4,325806434	5,64668E-16	5,26088E-13	NR2E3	10002	NR2E3	DOWN	0
9,5133045	-39,51930382	5,716730808	7,37288E-13	3,41198E-10	TMEM204	79652	TMEM204	DOWN	0
7,829274857	-27,45395418	6,389118202	3,92016E-07	7,02982E-05	WFIKKN2	124857	WFIKKN2	DOWN	0
9,496009966	-23,35819425	8,302662716	4,06368E-06	0,000515907	PGLYRP1	8993	PGLYRP1	DOWN	0
5,562799618	34,11855223	4,21104517	1,35095E-16	1,93808E-13	SLX1B	79008	SLX1B	UP	0
6,386418515	-24,63266918	6,965489754	1,46569E-06	0,000216772	SPMIP3	200159	SPMIP3	DOWN	1
5,213978543	-21,97258869	9,023792376	6,82982E-06	0,000815407	KRT1	3848	KRT1	DOWN	0
5,180308848	-10,48793668	10,4970943	8,89014E-05	0,00667738	GCKR	2646	GCKR	DOWN	1
5,059652167	-18,03492443	10,58781361	2,04839E-05	0,002099017	PHGR1	644844	PHGR1	DOWN	1
4,698248441	-16,2853801	10,99810514	3,0292E-05	0,002897127	GPR12	2835	GPR12	DOWN	1

baseMean	log2FoldChange	lfcSE	pvalue	padj	Symbol	ENTREZ	delabel	diffexp	UNIQUE
4,698248441	-16,2853801	10,99810514	3,0292E-05	0,002897127	SYT16	83851	SYT16	DOWN	1
