# Supplementary material for: The NF-κB Isoform p65 iso5 Is Associated with Distinct Transcriptional Programs and Signaling Pathways
Source: Cells. 2026 Sep 10;15(18):1643. doi: 10.3390/cells15181643 (PMC13605157; doi:10.3390/cells15181643)
Supplement: Supplementary file 1 [file cells-15-01643-s001.zip › TABLE S3.pdf]

ENTREZ	baseMean	log2FoldChange	lfcSE	stat	pvalue	padj	Symbol	delabel	diffexp	UNIQUE
10002	6,177173093	-34,46549614	4,402751475	-7,828172073	4,95014E-15	2,51883E-13	NR2E3	NR2E3	DOWN	0
1002	16,84734814	-6,486012074	2,331635004	-2,781744168	0,005406764	0,029835991	CDH4	CDH4	DOWN	1
100381270	22,39388256	6,048424737	1,915979818	3,156831131	0,001594937	0,011149474	ZBED6	ZBED6	UP	0
100419008	55,369039	8,340448742	1,484752009	5,61740189	1,9385E-08	4,44842E-07	PRR20G	PRR20G	UP	1
100533111	15,50892535	-5,420839802	2,080148427	-2,605987021	0,009160993	0,045558424	ZNF670-ZNF695	ZNF670-ZNF695	DOWN	1
10076	2026,739077	1,916321227	0,159814402	11,99091697	3,96491E-33	6,40038E-31	PTPRU	PTPRU	UP	1
10083	304,637827	1,350713939	0,444106241	3,041420755	0,002354645	0,015267147	USH1C	USH1C	UP	0
1009	125,3391382	-2,536497361	0,856447521	-2,961649488	0,003059959	0,018873108	CDH11	CDH11	DOWN	1
10100	116,2457312	-1,573488471	0,606289913	-2,595274039	0,009451553	0,046656452	TSPAN2	TSPAN2	DOWN	1
101059915	16,30976787	-8,093203722	2,854239557	-2,835502613	0,004575364	0,026035824	LOC101059915	LOC101059915	DOWN	1
10107	50,71680315	8,30160084	1,725110376	4,812214311	1,49267E-06	2,46624E-05	TRIM10	TRIM10	UP	0
10125	163,6984759	-1,612699365	0,539334825	-2,990163604	0,002788281	0,017536325	RASGRP1	RASGRP1	DOWN	1
10129	399,7652895	1,240283186	0,441674152	2,808140755	0,004982844	0,027980434	FRY	FRY	UP	1
1013	17,68932167	7,365269562	2,131569764	3,455326534	0,000549627	0,004521944	CDH15	CDH15	UP	0
1014	21,29662872	7,876159595	2,259571809	3,485686786	0,000490875	0,004105929	CDH16	CDH16	UP	1
10149	2073,350966	1,772613265	0,176363333	10,05091727	9,10162E-24	9,24114E-22	ADGRG2	ADGRG2	UP	1
10157	316,9868913	1,292643385	0,445827666	2,899423889	0,003738491	0,022196725	AASS	AASS	UP	1
10160	6130,787114	1,33268828	0,093860915	14,1985434	9,35364E-46	2,57574E-43	FARP1	FARP1	UP	1
10186	544,9557976	1,076735149	0,357591397	3,011076772	0,00260323	0,016587915	LHFPL6	LHFPL6	UP	1
10202	400,9324812	4,646331004	0,442184325	10,50767912	7,96291E-26	9,01863E-24	DHRS2	DHRS2	UP	0
10234	8,171203785	34,28258566	5,892215271	5,818284648	5,94546E-09	1,49104E-07	LRRC17	LRRC17	UP	1
10253	324,5266811	-1,268013583	0,307478457	-4,123910327	3,72494E-05	0,000431982	SPRY2	SPRY2	DOWN	0
1026	7704,077853	1,830911102	0,091558399	19,99719429	5,82591E-89	3,89615E-86	CDKN1A	CDKN1A	UP	1
1028	33705,99572	3,68127019	0,070944611	51,8893565	0	0	CDKN1C	CDKN1C	UP	1
1032	469,7235615	1,351900718	0,282868099	4,779261865	1,7594E-06	2,87314E-05	CDKN2D	CDKN2D	UP	1
10347	2619,427491	1,278678203	0,169000717	7,566111132	3,84563E-14	1,79429E-12	ABCA7	ABCA7	UP	1
10371	427,5984591	-1,030474852	0,281762217	-3,65724995	0,000254936	0,002337022	SEMA3A	SEMA3A	DOWN	1
10439	4869,306397	-1,222821407	0,125172244	-9,769109885	1,52788E-22	1,35808E-20	OLFM1	OLFM1	DOWN	1
1051	8369,86408	1,247834153	0,080056238	15,58696958	8,92689E-55	3,48248E-52	CEBPB	CEBPB	UP	1
10516	5823,20659	2,228043454	0,142251565	15,66269906	2,721E-55	1,09182E-52	FBLN5	FBLN5	UP	1
1052	1012,730778	1,007277478	0,20450738	4,925384501	8,41946E-07	1,44906E-05	CEBPD	CEBPD	UP	1
10529	1001,163293	3,662341336	0,311295115	11,76485321	5,92293E-32	9,14083E-30	NEBL	NEBL	UP	1
10561	2271,282454	-1,17333176	0,246005744	-4,769529934	1,84656E-06	2,99805E-05	IFI44	IFI44	DOWN	0

ENTREZ	baseMean	log2FoldChange	lfcSE	stat	pvalue	padj	Symbol	delabel	diffexp	UNIQUE
10578	10,9854565	31,18644366	3,223161014	9,675732464	3,82344E-22	3,35602E-20	GNLY	GNLY	UP	1
10610	28,75373509	5,290097488	1,848196605	2,862302352	0,004205755	0,024356957	ST6GALNAC2	ST6GALNAC2	UP	1
10644	166,7601035	2,694775888	0,683843267	3,940633795	8,12666E-05	0,0008627	IGF2BP2	IGF2BP2	UP	1
107	25,40264036	-6,121980806	2,108301286	-2,903750449	0,003687219	0,021951381	ADCY1	ADCY1	DOWN	0
10769	5522,375882	-1,070475559	0,11068506	-9,671364448	3,99022E-22	3,45918E-20	PLK2	PLK2	DOWN	1
107984640	59,80604571	6,019996143	1,57450575	3,8234196	0,000131614	0,001325954	LBHD2	LBHD2	UP	1
10826	66,82622939	3,49673935	1,189909495	2,938659929	0,003296345	0,020032885	FAXDC2	FAXDC2	UP	1
10882	1597,716562	1,370262397	0,231401511	5,921579309	3,18864E-09	8,35472E-08	C1QL1	C1QL1	UP	0
10891	400,1926959	-1,281069618	0,419134871	-3,056461549	0,002239662	0,014636488	PPARGC1A	PPARGC1A	DOWN	1
10957	2384,548601	1,129085579	0,152708668	7,393722943	1,42774E-13	6,28563E-12	PNRC1	PNRC1	UP	1
10964	2115,431028	1,616806492	0,25028728	6,459802883	1,04839E-10	3,30869E-09	IFI44L	IFI44L	UP	0
11005	612,5892992	1,025728054	0,279524837	3,669541725	0,000242986	0,002246538	SPINK5	SPINK5	UP	0
11009	126,4800099	2,879010883	0,964777797	2,984118095	0,00284397	0,017790963	IL24	IL24	UP	1
11057	4917,197135	1,227086494	0,134383485	9,131229858	6,77255E-20	4,97977E-18	ABHD2	ABHD2	UP	1
11067	13,29548079	7,605461915	2,633941277	2,887483477	0,00388337	0,022855203	DEPP1	DEPP1	UP	1
11095	1028,45226	1,862346825	0,289527371	6,432368791	1,2563E-10	3,92952E-09	ADAMTS8	ADAMTS8	UP	1
11138	1221,012943	1,987871467	0,216414452	9,185483919	4,09681E-20	3,11003E-18	TBC1D8	TBC1D8	UP	1
11151	207,5040807	2,569122628	0,612783381	4,19254619	2,75841E-05	0,00033167	CORO1A	CORO1A	UP	1
11170	51,01788482	6,069858286	1,939993369	3,128803625	0,001755196	0,012012655	FAM107A	FAM107A	UP	1
11173	544,7237824	2,952313987	0,342060156	8,630978889	6,08291E-18	3,99198E-16	ADAMTS7	ADAMTS7	UP	1
11213	460,1481793	2,670521253	0,561287432	4,757849719	1,95666E-06	3,1513E-05	IRAK3	IRAK3	UP	1
1122	6682,874448	1,395909186	0,124700569	11,19408833	4,35863E-29	5,82977E-27	CHML	CHML	UP	1
11238	155,2329993	-1,961966119	0,755502242	-2,596903105	0,009406846	0,046533899	CA5B	CA5B	DOWN	1
112487	654,7273223	-1,176283266	0,380194922	-3,09389526	0,001975472	0,013192359	DTD2	DTD2	DOWN	1
11254	15,4838595	-7,907139616	2,305062981	-3,430335606	0,000602835	0,004902268	SLC6A14	SLC6A14	DOWN	1
11264	831,9543097	1,445173201	0,348684802	4,144640636	3,40347E-05	0,000400993	PXMP4	PXMP4	UP	1
112817	68,30931439	8,840895145	1,648974627	5,36145008	8,25565E-08	1,72277E-06	HOGA1	HOGA1	UP	1
113622	3326,222074	2,693546756	0,184216125	14,62166659	2,04336E-48	5,85653E-46	ADPRHL1	ADPRHL1	UP	1
114804	198,1220902	2,195456711	0,671298863	3,270460939	0,001073724	0,007986957	RNF157	RNF157	UP	1
114879	4396,184517	1,405575942	0,126319988	11,12710635	9,25926E-29	1,20405E-26	OSBPL5	OSBPL5	UP	1
114881	1324,799976	1,371761048	0,153670728	8,92662554	4,39201E-19	3,06873E-17	OSBPL7	OSBPL7	UP	1
114898	97,44590954	3,326633975	0,832618251	3,995389209	6,45881E-05	0,000708099	C1QTNF2	C1QTNF2	UP	1
114907	1048,212446	-1,756960516	0,30500915	-5,760353476	8,3938E-09	2,05013E-07	FBXO32	FBXO32	DOWN	1

ENTREZ	baseMean	log2FoldChange	lfcSE	stat	pvalue	padj	Symbol	delabel	diffexp	UNIQUE
114987	143,136866	1,751870332	0,515465126	3,398620475	0,000677266	0,005380026	WDR31	WDR31	UP	1
115572	1946,30892	1,964530915	0,173374293	11,33115459	9,19841E-30	1,2665E-27	TENT5B	TENT5B	UP	1
115704	621,9344639	2,43999336	0,655616532	3,721677598	0,000197904	0,001877945	EVI5L	EVI5L	UP	1
116154	99,56953345	6,395944058	0,998165224	6,407700754	1,4773E-10	4,6105E-09	PHACTR3	PHACTR3	UP	1
116372	589,5308755	-1,377447284	0,409049712	-3,367432474	0,000758716	0,005959401	LYPD1	LYPD1	DOWN	1
1164	5432,104316	-1,038228339	0,116449069	-8,915728986	4,84617E-19	3,36929E-17	CKS2	CKS2	DOWN	1
117283	57,24241835	8,995092022	2,500441306	3,597401786	0,000321412	0,002849689	IP6K3	IP6K3	UP	1
118429	614,1988459	-2,792575474	0,734162075	-3,803758829	0,000142517	0,001416495	ANTXR2	ANTXR2	DOWN	1
1187	12,424494	6,300634962	2,401062374	2,624102993	0,008687751	0,043575277	CLCNKA	CLCNKA	UP	1
121227	509,8338477	-1,299361304	0,424290158	-3,062435644	0,002195437	0,014367528	LRIG3	LRIG3	DOWN	1
121512	1278,668414	2,852896115	0,271681407	10,50088831	8,5571E-26	9,61408E-24	FGD4	FGD4	UP	1
121601	586,907184	1,340654327	0,294658858	4,549852451	5,36835E-06	7,79661E-05	ANO4	ANO4	UP	1
122402	1966,021482	1,097834822	0,256749218	4,27590327	1,90364E-05	0,00023659	TDRD9	TDRD9	UP	1
122622	656,6578654	1,394504873	0,315025776	4,426637366	9,57134E-06	0,00012925	ADSS1	ADSS1	UP	1
123	868,2585874	1,663946354	0,332536795	5,003796204	5,62122E-07	1,00694E-05	PLIN2	PLIN2	UP	1
1233	29,20255174	7,024357833	1,766936407	3,975444619	7,02479E-05	0,000759478	CCR4	CCR4	UP	1
1238	17,79347793	6,678586053	2,319281822	2,87959229	0,003981897	0,023320169	ACKR2	ACKR2	UP	1
1244	75,80539571	2,53935418	0,929270311	2,732632422	0,006283041	0,033795108	ABCC2	ABCC2	UP	1
1268	414,2332014	3,536198285	0,552974001	6,394872599	1,60681E-10	4,9925E-09	CNR1	CNR1	UP	1
127294	387,7133655	1,241589143	0,418022314	2,970150399	0,00297654	0,018492043	MYOM3	MYOM3	UP	1
1280	26,02162097	8,206661173	2,37125109	3,460899273	0,000538374	0,00444499	COL2A1	COL2A1	UP	1
128218	21,15042613	19,24538129	2,693042682	7,146333556	8,91263E-13	3,60718E-11	TMEM125	TMEM125	UP	0
1296	35,91463676	6,960813809	1,947874823	3,573542677	0,000352184	0,003074002	COL8A2	COL8A2	UP	0
129804	46,84822135	8,186310426	1,718138871	4,764638392	1,89193E-06	3,06108E-05	FBLN7	FBLN7	UP	0
129807	32,07877317	7,174007552	2,110865103	3,398610145	0,000677292	0,005380026	NEU4	NEU4	UP	1
130612	204,8977409	1,39988893	0,501067536	2,793812872	0,005209061	0,029030181	TMEM198	TMEM198	UP	1
1307	938,153233	-1,045090905	0,218045857	-4,792986751	1,64316E-06	2,69587E-05	COL16A1	COL16A1	DOWN	1
132321	463,913526	1,604377327	0,371405426	4,319746602	1,56208E-05	0,000198533	C4orf33	C4orf33	UP	1
133522	1286,008718	1,519830222	0,193616758	7,849683241	4,17089E-15	2,15353E-13	PPARGC1B	PPARGC1B	UP	1
134265	856,0279507	1,420088311	0,267196627	5,314768863	1,06793E-07	2,18311E-06	AFAP1L1	AFAP1L1	UP	1
1363	756,0787661	1,376290917	0,305650847	4,502820554	6,70575E-06	9,46488E-05	CPE	CPE	UP	1
1366	156,683028	2,800991923	0,749703471	3,73613306	0,000186872	0,001787757	CLDN7	CLDN7	UP	1
1368	1417,736341	1,862483756	0,174198165	10,69175301	1,11248E-26	1,33536E-24	CPM	CPM	UP	1

ENTREZ	baseMean	log2FoldChange	lfcSE	stat	pvalue	padj	Symbol	delabel	diffexp	UNIQUE
137075	154,5909691	2,019456471	0,69466921	2,907076407	0,003648241	0,02177471	CLDN23	CLDN23	UP	1
1396	240,6215789	1,238903729	0,421004818	2,942730526	0,003253315	0,019839145	CRIP1	CRIP1	UP	1
139818	609,7660367	1,74100037	0,4022745	4,327891449	1,50544E-05	0,000192729	DOCK11	DOCK11	UP	0
140831	163,3444266	1,70726157	0,608442195	2,805955246	0,005016766	0,028147957	ZSWIM3	ZSWIM3	UP	1
140885	1310,294044	1,118659124	0,200401899	5,582078458	2,37661E-08	5,3921E-07	SIRPA	SIRPA	UP	1
141	130,4839101	2,184907943	0,850529096	2,568880894	0,010202752	0,049580431	ADPRH	ADPRH	UP	1
1410	1642,654215	1,527784944	0,226697775	6,739302774	1,59148E-11	5,5599E-10	CRYAB	CRYAB	UP	1
1438	435,7547721	1,009477076	0,336032275	3,004107499	0,002663612	0,01689601	CSF2RA	CSF2RA	UP	0
144100	2090,383708	1,711392951	0,172526598	9,919589029	3,42162E-23	3,18233E-21	PLEKHA7	PLEKHA7	UP	1
145748	595,6864609	1,229874511	0,37292622	3,297903029	0,000974098	0,007346225	LYSMD4	LYSMD4	UP	1
146198	246,0494208	1,597807897	0,52795702	3,026397672	0,002474866	0,01588529	ZFP90	ZFP90	UP	1
146330	1139,238304	1,095437408	0,185539774	5,904057019	3,54669E-09	9,25832E-08	FBXL16	FBXL16	UP	0
146664	422,8544826	-1,294761038	0,341117475	-3,795645586	0,00014726	0,001455395	MGAT5B	MGAT5B	DOWN	1
147	316,2197831	1,262584898	0,451317783	2,797551851	0,00514915	0,028719089	ADRA1B	ADRA1B	UP	1
147015	508,6925395	1,441201044	0,319492016	4,510914115	6,45489E-06	9,14757E-05	DHRS13	DHRS13	UP	1
147138	13,42260465	-40,10211734	3,10364444	-12,92097665	3,42763E-38	6,50508E-36	TMC8	TMC8	DOWN	0
147381	61,99605561	-8,314255657	1,759911898	-4,724245381	2,30971E-06	3,657E-05	CBLN2	CBLN2	DOWN	0
147710	29,9627918	7,502445436	2,208030876	3,397799151	0,000679303	0,005392949	IGSF23	IGSF23	UP	1
147837	23,83252367	8,059037265	2,314866464	3,481426419	0,000498751	0,004159416	ZNF563	ZNF563	UP	1
147906	55,0246853	3,971608936	1,179802636	3,366333328	0,000761746	0,005973177	DACT3	DACT3	UP	1
148229	335,0039549	1,898495575	0,631586014	3,005917695	0,002647807	0,016810938	ATP8B3	ATP8B3	UP	1
150	1019,74237	-2,03601197	0,247058678	-8,241005697	1,70769E-16	9,95137E-15	ADRA2A	ADRA2A	DOWN	1
151056	128,5987922	2,770127338	0,843550177	3,283891595	0,001023843	0,00766872	PLB1	PLB1	UP	1
151176	66,41372432	3,340842153	1,068736905	3,12597248	0,001772181	0,012099424	ERFE	ERFE	UP	1
151242	25,35066198	-6,350162774	2,08915421	-3,039585466	0,00236904	0,015339232	PPP1R1C	PPP1R1C	DOWN	0
153020	67,07608838	2,869539218	1,087565335	2,638498237	0,008327413	0,042113861	RASGEF1B	RASGEF1B	UP	1
153562	247,4171953	1,276653156	0,446578342	2,858744	0,004253219	0,024566757	MARVELD2	MARVELD2	UP	1
1543	201,1997846	1,252828804	0,487868464	2,567964312	0,010229769	0,049677342	CYP1A1	CYP1A1	UP	1
155185	87,86598063	2,480579783	0,845771086	2,932921002	0,003357893	0,020353152	AMZ1	AMZ1	UP	1
155382	19,1411384	6,806103172	2,233485307	3,04730152	0,00230906	0,015013164	VPS37D	VPS37D	UP	1
157638	14,32616844	7,390564705	2,559472466	2,887534366	0,003882742	0,022855203	LRATD2	LRATD2	UP	1
1580	45,2503563	9,035054783	1,684709954	5,362973469	8,1863E-08	1,71084E-06	CYP4B1	CYP4B1	UP	1
158219	158,3673801	1,371990563	0,513397191	2,67237645	0,00753161	0,039002189	TTC39B	TTC39B	UP	1

ENTREZ	baseMean	log2FoldChange	lfcSE	stat	pvalue	padj	Symbol	delabel	diffexp	UNIQUE
158763	4,614209061	-26,81840749	5,891968933	-4,551688543	5,32171E-06	7,74488E-05	ARHGAP36	ARHGAP36	DOWN	1
159195	866,1488563	1,515614342	0,293958026	5,155886929	2,52433E-07	4,80376E-06	USP54	USP54	UP	1
160428	219,3503372	1,76507666	0,609127347	2,8977137	0,003758936	0,022255688	ALDH1L2	ALDH1L2	UP	1
161176	184,4863137	2,482497341	0,716491435	3,464796953	0,000530632	0,004386224	SYNE3	SYNE3	UP	1
162282	178,0721967	1,503021795	0,5414914	2,775707599	0,005508172	0,030265833	ANKFN1	ANKFN1	UP	1
162494	311,5183682	1,762184207	0,667305355	2,640746389	0,008272362	0,041926037	RHBDL3	RHBDL3	UP	1
163259	148,2027855	1,57866548	0,431436237	3,659093384	0,000253109	0,00232331	DENND2C	DENND2C	UP	1
1634	2876,344864	2,902115182	0,148464615	19,54752108	4,33059E-85	2,53411E-82	DCN	DCN	UP	1
1645	604,299962	-1,106173073	0,296951845	-3,725092438	0,000195244	0,001860246	AKR1C1	AKR1C1	DOWN	1
168667	304,3906681	-2,360406197	0,510286756	-4,625646599	3,73432E-06	5,65747E-05	BMPER	BMPER	DOWN	1
1687	2712,823191	1,089815549	0,155481921	7,009275035	2,39556E-12	8,94768E-11	GSDME	GSDME	UP	1
1734	3783,704721	-1,34007405	0,155937026	-8,59368737	8,4222E-18	5,42575E-16	DIO2	DIO2	DOWN	1
1745	759,1193697	-1,007545825	0,313835485	-3,210426719	0,001325381	0,009550357	DLX1	DLX1	DOWN	1
1746	818,8418608	-1,59289283	0,294077535	-5,416574331	6,07517E-08	1,29665E-06	DLX2	DLX2	DOWN	1
1747	146,6568684	-1,51442056	0,511508988	-2,960691984	0,003069487	0,018915262	DLX3	DLX3	DOWN	1
1755	465,5251047	1,249004515	0,417896066	2,988792229	0,002800825	0,017591587	DMBT1	DMBT1	UP	1
1808	3375,02577	1,620473986	0,122539716	13,22407171	6,37191E-40	1,31599E-37	DPYSL2	DPYSL2	UP	1
1831	6270,216472	2,831407344	0,108213889	26,16491629	6,6676E-151	1,1705E-147	TSC22D3	TSC22D3	UP	1
1836	3386,962231	1,595481278	0,125760592	12,68665528	7,01167E-37	1,26246E-34	SLC26A2	SLC26A2	UP	1
1842	15,3659526	6,288375486	2,418072122	2,600573998	0,009306794	0,046152762	ECM2	ECM2	UP	1
1843	12535,23984	2,162965244	0,085731373	25,22956488	1,8985E-140	2,6663E-137	DUSP1	DUSP1	UP	1
1844	1366,305591	-1,309428689	0,170921834	-7,660979616	1,8452E-14	8,81429E-13	DUSP2	DUSP2	DOWN	1
1847	626,8724474	1,739290123	0,376392332	4,620949938	3,81987E-06	5,76841E-05	DUSP5	DUSP5	UP	1
1870	1653,72165	1,131735959	0,219586035	5,153952336	2,55053E-07	4,84704E-06	E2F2	E2F2	UP	1
1907	1189,271168	1,059945066	0,223158903	4,749732371	2,03686E-06	3,25805E-05	EDN2	EDN2	UP	0
1942	1235,595247	-1,072719294	0,240871234	-4,453496909	8,44829E-06	0,000116094	EFNA1	EFNA1	DOWN	1
1958	190,6196652	2,072031536	0,672691039	3,080212783	0,002068528	0,013677213	EGR1	EGR1	UP	1
199800	27,51339529	-9,075042591	1,919993086	-4,726601703	2,28308E-06	3,62711E-05	ADM5	ADM5	DOWN	1
200150	24,44850937	8,231120911	1,989394547	4,137500489	3,5111E-05	0,000410915	PLD5	PLD5	UP	1
2006	1245,89665	1,861623112	0,339277555	5,48702112	4,08768E-08	8,95592E-07	ELN	ELN	UP	0
201164	400,5298761	-1,341644451	0,39135527	-3,428200801	0,000607596	0,004926718	PLD6	PLD6	DOWN	1
2012	9213,040885	1,56047558	0,071566878	21,80443836	2,1057E-105	1,9715E-102	EMP1	EMP1	UP	1
201255	3898,920011	1,316240277	0,142382402	9,244402831	2,36558E-20	1,8254E-18	LRRC45	LRRC45	UP	1

ENTREZ	baseMean	log2FoldChange	lfcSE	stat	pvalue	padj	Symbol	delabel	diffexp	UNIQUE
2013	7560,147824	1,509820382	0,102677847	14,70444145	6,0367E-49	1,76624E-46	EMP2	EMP2	UP	1
2027	3122,229927	-1,335683023	0,163820876	-8,153313884	3,54085E-16	2,04641E-14	ENO3	ENO3	DOWN	1
203238	181,9984935	2,125938836	0,736415025	2,886875963	0,003890876	0,022882521	CCDC171	CCDC171	UP	1
203260	698,5336889	1,298122269	0,201432719	6,444445948	1,16023E-10	3,64527E-09	CCDC107	CCDC107	UP	1
2034	4102,740534	1,558558674	0,110276327	14,13321164	2,37081E-45	6,403E-43	EPAS1	EPAS1	UP	1
2040	25897,20199	1,27863189	0,061083391	20,93256229	2,70555E-97	1,99983E-94	STOM	STOM	UP	1
2048	575,2526411	-1,286607773	0,271422399	-4,740241694	2,13463E-06	3,40282E-05	EPHB2	EPHB2	DOWN	0
2053	107,1522408	2,606331464	0,978767715	2,662870284	0,007747727	0,03982763	EPHX2	EPHX2	UP	1
2059	2553,610959	1,433805809	0,197633257	7,254881228	4,02014E-13	1,70057E-11	EPS8	EPS8	UP	1
2065	24,31973732	8,4027542	2,202322836	3,815405291	0,00013596	0,001363789	ERBB3	ERBB3	UP	1
2072	779,6780239	-1,15717967	0,261770032	-4,420596431	9,84288E-06	0,000132534	ERCC4	ERCC4	DOWN	1
2081	609,0503013	2,40401114	0,773839498	3,106601752	0,001892512	0,01275719	ERN1	ERN1	UP	1
2139	10,92104291	-19,54545285	2,959275636	-6,604809845	3,98028E-11	1,35022E-09	EYA2	EYA2	DOWN	0
2149	292,2790901	2,096331252	0,458036581	4,57677692	4,72195E-06	6,97319E-05	F2R	F2R	UP	1
2150	404,2499854	3,228008133	0,567250136	5,69062558	1,26575E-08	2,98258E-07	F2RL1	F2RL1	UP	1
2159	18,07896349	8,360197329	2,530141356	3,304241207	0,000952339	0,007206165	F10	F10	UP	1
2185	1789,136144	1,851284689	0,166258786	11,13495852	8,47845E-29	1,12331E-26	PTK2B	PTK2B	UP	1
219699	99,16481105	2,867249986	0,739311957	3,878268108	0,000105203	0,001086373	UNC5B	UNC5B	UP	1
220213	371,1759207	-1,483331954	0,461359321	-3,215133815	0,001303837	0,009419285	OTUD1	OTUD1	DOWN	1
220963	182,661824	-1,556704961	0,539564892	-2,885111658	0,003912749	0,0229919	SLC16A9	SLC16A9	DOWN	1
221002	13,1884961	-18,83996217	2,897806107	-6,501457127	7,95457E-11	2,56225E-09	RASGEF1A	RASGEF1A	DOWN	0
221188	38,44083333	8,32038044	1,883719243	4,416996041	1,00082E-05	0,000134118	ADGRG5	ADGRG5	UP	1
221481	13,82542155	7,876676391	2,500704342	3,149783147	0,001633917	0,011359767	ARMC12	ARMC12	UP	1
221527	283,1434902	-1,584555969	0,459726289	-3,446737782	0,000567399	0,004646386	ZBTB12	ZBTB12	DOWN	1
222235	118,5952672	-1,710402638	0,551771772	-3,099837148	0,001936271	0,012986144	FBXL13	FBXL13	DOWN	1
222950	54,41201676	3,620777371	1,156513542	3,130769541	0,001743489	0,011950006	NYAP1	NYAP1	UP	1
222962	36,9609185	8,254715666	2,290168914	3,604413463	0,000312859	0,002786168	SLC29A4	SLC29A4	UP	1
2244	56,78867345	9,244539355	1,866914586	4,951774133	7,35399E-07	1,29099E-05	FGB	FGB	UP	1
2257	595,0893533	-1,660267833	0,427803492	-3,8809123	0,000104065	0,001078593	FGF12	FGF12	DOWN	1
22795	214,076165	2,03202563	0,604028565	3,364121745	0,000767877	0,006011181	NID2	NID2	UP	1
22822	552,0594409	-1,112566275	0,30824262	-3,609384955	0,000306924	0,002742009	PHLDA1	PHLDA1	DOWN	0
22885	2385,490698	3,289057612	0,249071781	13,20525994	8,18168E-40	1,66527E-37	ABLIM3	ABLIM3	UP	1
2289	13908,80316	3,30853652	0,089700018	36,88445781	8,2024E-298	2,8799E-294	FKBP5	FKBP5	UP	1

ENTREZ	baseMean	log2FoldChange	lfcSE	stat	pvalue	padj	Symbol	delabel	diffexp	UNIQUE
22943	2815,824125	2,084565059	0,220048583	9,473203753	2,7139E-21	2,21593E-19	DKK1	DKK1	UP	1
22987	18,31113746	-20,1218415	3,947012891	-5,097992345	3,43275E-07	6,38536E-06	SV2C	SV2C	DOWN	0
23034	1632,875385	1,959530342	0,197317523	9,930848046	3,05645E-23	2,86165E-21	SAMD4A	SAMD4A	UP	1
23051	1566,295198	1,239518752	0,237685314	5,21495726	1,8386E-07	3,61136E-06	ZHX3	ZHX3	UP	1
23086	106,6707795	2,534477067	0,727007543	3,486177127	0,000489976	0,004100852	EXPH5	EXPH5	UP	1
23105	1291,139371	-1,30194488	0,171561148	-7,588809581	3,22858E-14	1,51646E-12	FSTL4	FSTL4	DOWN	0
23151	940,3919284	1,910549267	0,210008511	9,097484935	9,24478E-20	6,69246E-18	GRAMD4	GRAMD4	UP	1
23175	2207,03447	2,330915151	0,240870604	9,677042839	3,77476E-22	3,33414E-20	LPIN1	LPIN1	UP	0
23208	188,953672	2,275671987	0,492197041	4,623497901	3,77323E-06	5,70411E-05	SYT11	SYT11	UP	1
23224	2007,575435	1,455892153	0,227991294	6,385735731	1,70575E-10	5,27656E-09	SYNE2	SYNE2	UP	0
23266	699,1817628	-1,075771541	0,300891556	-3,575279925	0,000349853	0,003061271	ADGRL2	ADGRL2	DOWN	1
23281	406,9925457	2,840466847	0,768395758	3,696619636	0,000218489	0,002047007	MTUS2	MTUS2	UP	1
23284	30,87438335	7,934798205	2,312263818	3,43161457	0,0006	0,0048877	ADGRL3	ADGRL3	UP	1
23303	286,4948788	1,940734907	0,615744648	3,151850225	0,001622395	0,011307648	KIF13B	KIF13B	UP	1
23308	21,92942084	-4,604057147	1,469523663	-3,133026887	0,001730136	0,011870068	ICOSLG	ICOSLG	DOWN	1
2335	18379,05632	1,756278002	0,157765024	11,13223934	8,74115E-29	1,1473E-26	FN1	FN1	UP	1
23363	3400,743387	1,876509736	0,148689967	12,62028486	1,6323E-36	2,90177E-34	OBSL1	OBSL1	UP	1
23371	2308,769968	2,431808527	0,164592397	14,77473187	2,13204E-49	6,37072E-47	TNS2	TNS2	UP	1
23428	127,9411086	10,23893366	1,907215997	5,368523374	7,93839E-08	1,6615E-06	SLC7A8	SLC7A8	UP	1
23508	270,7879426	1,624695217	0,615589158	2,639252489	0,008308907	0,042065715	TTC9	TTC9	UP	1
2353	223,3518426	2,111950489	0,637722893	3,31170562	0,000927291	0,007047008	FOS	FOS	UP	1
2354	50,24288557	3,126070829	1,210945788	2,58151179	0,009836863	0,048185876	FOSB	FOSB	UP	1
23541	308,7985094	2,196465844	0,357236922	6,148484965	7,82265E-10	2,25126E-08	SEC14L2	SEC14L2	UP	1
23551	19,52032852	7,633715653	2,270544282	3,362064203	0,000773621	0,006049409	RASD2	RASD2	UP	1
23586	2534,459448	-1,223727335	0,317399004	-3,855485744	0,0001155	0,001180555	RIGI	RIGI	DOWN	0
23596	351,8101345	1,185110193	0,353138751	3,35593358	0,000790976	0,006165318	OPN3	OPN3	UP	1
23623	4057,022198	1,090913215	0,096891516	11,25912006	2,08833E-29	2,84743E-27	RUSC1	RUSC1	UP	1
23635	947,9764232	1,509413941	0,330988689	4,560318806	5,1076E-06	7,46422E-05	SSBP2	SSBP2	UP	1
23650	6596,658335	1,29921059	0,104823252	12,39429772	2,80593E-35	4,80567E-33	TRIM29	TRIM29	UP	1
23657	6609,577923	-1,145408018	0,083843244	-13,66130362	1,72861E-42	4,0461E-40	SLC7A11	SLC7A11	DOWN	1
23705	653,3557748	-1,240263405	0,307810752	-4,02930501	5,5942E-05	0,000623037	CADM1	CADM1	DOWN	1
23767	126,8751219	3,26510127	1,053181441	3,100226745	0,001933725	0,012981473	FLRT3	FLRT3	UP	1
239	17,42635834	-5,558585342	2,164923711	-2,567566383	0,010241518	0,049700026	ALOX12	ALOX12	DOWN	1

ENTREZ	baseMean	log2FoldChange	lfcSE	stat	pvalue	padj	Symbol	delabel	diffexp	UNIQUE
24	171,7637818	2,042739494	0,542018077	3,768766358	0,000164056	0,001592265	ABCA4	ABCA4	UP	1
241	54,12189861	3,959952958	1,52083161	2,603807635	0,009219449	0,045784277	ALOX5AP	ALOX5AP	UP	1
250	1558,990084	4,856338635	0,460696264	10,54130241	5,57218E-26	6,4144E-24	ALPP	ALPP	UP	1
2534	1729,694481	1,248682802	0,188297947	6,631420143	3,32472E-11	1,13607E-09	FYN	FYN	UP	1
254268	14,03900648	7,595567978	2,608729913	2,911596152	0,003595872	0,021507849	AKNAD1	AKNAD1	UP	1
254427	2027,07487	1,409144138	0,165314765	8,524006537	1,54127E-17	9,57769E-16	PROSER2	PROSER2	UP	1
254552	326,7762687	1,21738319	0,409961277	2,969507749	0,002982773	0,018510854	NUDT8	NUDT8	UP	1
254827	17,86908843	-7,885062452	2,705757842	-2,914178915	0,003566255	0,0213732	NAALADL2	NAALADL2	DOWN	1
254887	505,0515064	1,183944827	0,325325184	3,639265827	0,000273416	0,002478929	ZDHHC23	ZDHHC23	UP	1
255324	521,7999636	1,073723393	0,266407726	4,030376335	5,56876E-05	0,000621189	EPGN	EPGN	UP	1
255743	6994,897773	1,621690316	0,082411195	19,67803431	3,32657E-86	2,03123E-83	NPNT	NPNT	UP	0
255783	449,5622402	1,622284436	0,349225777	4,645374264	3,3946E-06	5,18194E-05	INAFM1	INAFM1	UP	1
256691	1470,398831	-1,119143018	0,18820709	-5,946338259	2,74207E-09	7,23866E-08	MAMDC2	MAMDC2	DOWN	1
257000	68,76319073	3,142635687	1,092547342	2,876429758	0,004022017	0,023515906	TINCR	TINCR	UP	1
257019	927,8017604	3,11545032	0,323827538	9,620708424	6,53793E-22	5,53125E-20	FRMD3	FRMD3	UP	0
257194	1082,363032	-1,473777027	0,326822477	-4,509411477	6,50077E-06	9,20331E-05	NEGR1	NEGR1	DOWN	1
25822	979,8909964	1,125313782	0,354566053	3,173777558	0,00150469	0,010635058	DNAJB5	DNAJB5	UP	1
25837	310,7808262	1,749445481	0,662377997	2,641158806	0,008262298	0,04189015	RAB26	RAB26	UP	1
25840	712,4332115	3,094050805	0,774965848	3,992499558	6,53805E-05	0,000715669	TMT1A	TMT1A	UP	1
25841	4023,633678	1,015643399	0,164865142	6,160449597	7,25387E-10	2,09616E-08	ABTB2	ABTB2	UP	1
25862	377,0650932	2,012378283	0,601131482	3,347650796	0,000814996	0,006313187	USP49	USP49	UP	1
25884	35,16290531	-4,046511222	1,148438975	-3,523488239	0,000425906	0,003620717	CHRDL2	CHRDL2	DOWN	0
2591	845,1834558	-1,479266326	0,419479474	-3,526433154	0,000421197	0,003585029	GALNT3	GALNT3	DOWN	1
259173	2059,23838	1,261795058	0,148383994	8,503579264	1,83833E-17	1,13733E-15	ALS2CL	ALS2CL	UP	1
2593	1042,32169	1,055309691	0,361449115	2,919663232	0,003504098	0,02104857	GAMT	GAMT	UP	1
259307	22,27164086	-7,31401111	2,568195609	-2,847918237	0,004400623	0,025246057	IL4I1	IL4I1	DOWN	1
26027	279,1868044	2,019209522	0,610434681	3,30782242	0,000940244	0,007128877	ACOT11	ACOT11	UP	1
2619	6540,628497	1,000277513	0,083052623	12,04390038	2,08843E-33	3,45057E-31	GAS1	GAS1	UP	1
26207	4428,962176	1,793945854	0,116600184	15,38544615	2,04959E-53	7,77957E-51	PITPNC1	PITPNC1	UP	1
2621	880,253205	1,112285571	0,302876433	3,672407125	0,000240276	0,002224419	GAS6	GAS6	UP	1
26249	58,64531743	3,607544975	1,389772878	2,59578024	0,009437641	0,04662055	KLHL3	KLHL3	UP	1
2627	854,2872728	-1,196386992	0,300770397	-3,977741841	6,95728E-05	0,000752759	GATA6	GATA6	DOWN	1
26287	565,8530166	5,682309878	0,94756221	5,996767088	2,01284E-09	5,45722E-08	ANKRD2	ANKRD2	UP	1

ENTREZ	baseMean	log2FoldChange	lfcSE	stat	pvalue	padj	Symbol	delabel	diffexp	UNIQUE
26298	25,74586725	8,237700105	1,951767112	4,220636804	2,43613E-05	0,000298283	EHF	EHF	UP	1
26301	35,12890983	-9,020741896	1,797361264	-5,018880777	5,19734E-07	9,40611E-06	GBGT1	GBGT1	DOWN	0
26468	123,4333184	1,429046567	0,51070346	2,798192452	0,005138948	0,028684972	LHX6	LHX6	UP	1
26508	14,54929026	7,699078958	2,533192781	3,039278738	0,002371453	0,015347783	HEYL	HEYL	UP	1
2651	709,0209045	-1,13570148	0,23839581	-4,76393222	1,89856E-06	3,06829E-05	GCNT2	GCNT2	DOWN	1
26585	2366,426643	-1,277730392	0,189004934	-6,760301796	1,37705E-11	4,83481E-10	GREM1	GREM1	DOWN	1
2687	69,18446239	8,781650324	1,506020961	5,831027954	5,5087E-09	1,39144E-07	GGT5	GGT5	UP	1
26873	699,9578631	1,282891591	0,259606322	4,941680854	7,74519E-07	1,34787E-05	OPLAH	OPLAH	UP	1
2695	27,61904956	7,510355067	2,346927641	3,200079514	0,001373897	0,009844391	GIP	GIP	UP	1
2706	12,51881554	5,896004727	2,276135172	2,590357901	0,009587619	0,047178882	GJB2	GJB2	UP	1
27063	2779,251876	2,316588711	0,183969762	12,59222543	2,3301E-36	4,09049E-34	ANKRD1	ANKRD1	UP	1
27065	12,45967507	8,169512913	2,914353395	2,803199134	0,005059842	0,02833851	NSG1	NSG1	UP	1
2707	3517,334956	1,638402756	0,165858224	9,878332925	5,16856E-23	4,74426E-21	GJB3	GJB3	UP	0
27086	1262,802219	-1,213067121	0,216852204	-5,59398106	2,21921E-08	5,05951E-07	FOXP1	FOXP1	DOWN	0
27122	835,0506046	1,445983266	0,26156411	5,528217412	3,23501E-08	7,17733E-07	DKK3	DKK3	UP	1
27129	66,17372973	3,923228086	1,243091452	3,156025312	0,00159935	0,011174761	HSPB7	HSPB7	UP	1
27145	33,42970377	4,499137674	1,687567901	2,666048384	0,007674865	0,039586507	FILIP1	FILIP1	UP	0
27151	18,45553865	8,179510009	2,382639073	3,432962257	0,000597025	0,004869118	CPAMD8	CPAMD8	UP	1
27202	1662,636614	2,971282756	0,230155708	12,90988083	3,95913E-38	7,41361E-36	C5AR2	C5AR2	UP	1
27244	392,073355	1,098840278	0,341146179	3,221024724	0,001277331	0,009246825	SESN1	SESN1	UP	1
27284	89,26489781	5,466022768	1,268692636	4,30839008	1,64447E-05	0,000207875	SULT1B1	SULT1B1	UP	1
27345	310,4585744	1,814428679	0,603385824	3,007078735	0,002637714	0,016763333	KCNMB4	KCNMB4	UP	1
2752	13501,86802	1,529621994	0,076959438	19,8756908	6,60708E-88	4,21772E-85	GLUL	GLUL	UP	1
2766	630,8013346	5,830419382	1,073783399	5,429790951	5,64201E-08	1,20972E-06	GMPR	GMPR	UP	1
28231	4055,190618	1,859287074	0,098421228	18,89111851	1,34956E-79	7,28969E-77	SLCO4A1	SLCO4A1	UP	1
2825	2973,677686	1,209536407	0,164020312	7,37430867	1,652E-13	7,25022E-12	CMKLR2	CMKLR2	UP	1
283208	782,6864015	1,273141682	0,261434787	4,86982507	1,11697E-06	1,8809E-05	P4HA3	P4HA3	UP	1
283316	45,31358239	3,442441726	1,302502696	2,642944031	0,008218862	0,041760383	CD163L1	CD163L1	UP	0
283537	123,1358051	3,253373281	0,682751632	4,765090446	1,88769E-06	3,05775E-05	SLC46A3	SLC46A3	UP	1
283807	14,3769937	-6,314223715	2,216049663	-2,849315077	0,004381347	0,025159494	FBXL22	FBXL22	DOWN	1
283933	25,62645216	6,904326577	2,390663076	2,888038321	0,003876526	0,022845966	ZNF843	ZNF843	UP	1
283987	33,36655352	4,544113453	1,391560846	3,265479526	0,001092789	0,008120174	HID1	HID1	UP	1
2840	31,43135712	7,01882911	1,845108203	3,804020326	0,000142366	0,001416002	GPR17	GPR17	UP	0

ENTREZ	baseMean	log2FoldChange	lfcSE	stat	pvalue	padj	Symbol	delabel	diffexp	UNIQUE
284076	46,50705614	5,908505218	1,721241773	3,432699178	0,000597605	0,004871016	TTLL6	TTLL6	UP	0
284348	68,88719438	3,332256202	1,19450503	2,789654391	0,005276433	0,02932419	LYPD5	LYPD5	UP	1
285313	2943,466734	-1,940321818	0,197345042	-9,83212852	8,18698E-23	7,41793E-21	IGSF10	IGSF10	DOWN	0
286	62,20755627	3,661415358	1,137017783	3,220191815	0,001281048	0,009264184	ANK1	ANK1	UP	1
286343	689,0384195	1,298452147	0,36049224	3,601886535	0,000315916	0,002809834	LURAP1L	LURAP1L	UP	1
2875	47,68557313	4,069446488	1,348237985	3,018344338	0,002541599	0,016269016	GPT	GPT	UP	1
2882	189,4009545	1,366679141	0,527088775	2,592882275	0,009517534	0,046916199	GPX7	GPX7	UP	1
28984	360,6597136	3,665560441	0,657595254	5,57418932	2,48685E-08	5,61501E-07	RGCC	RGCC	UP	1
28996	3636,810227	1,015047057	0,156357338	6,491841489	8,47934E-11	2,72503E-09	HIPK2	HIPK2	UP	1
290	3331,144352	3,107953296	0,178165864	17,44415698	3,81303E-68	1,84656E-65	ANPEP	ANPEP	UP	1
2901	5,203081115	24,3613455	5,172804014	4,709504832	2,48319E-06	3,91403E-05	GRIK5	GRIK5	UP	1
2919	164,1427901	-2,431362606	0,910272065	-2,671028475	0,007561923	0,039115893	CXCL1	CXCL1	DOWN	0
2934	16406,53536	1,508955249	0,070753839	21,32683229	6,3998E-101	4,99325E-98	GSN	GSN	UP	1
29767	197,3749618	1,336495719	0,41964578	3,184818683	0,001448447	0,010310184	TMOD2	TMOD2	UP	1
29903	1372,262186	-1,057456655	0,225497092	-4,689446964	2,73944E-06	4,24644E-05	CCDC106	CCDC106	DOWN	1
29907	11,31821595	7,340299213	2,466210102	2,976347882	0,002917038	0,018183256	SNX15	SNX15	UP	1
29949	23,16984871	7,511312489	2,463558839	3,048968172	0,002296288	0,014942441	IL19	IL19	UP	1
30818	143,7412612	2,274779911	0,761162876	2,988558666	0,002802967	0,017597169	KCNIP3	KCNIP3	UP	1
3119	14,30253528	-19,85778747	2,790176993	-7,117035055	1,10273E-12	4,3748E-11	HLA-DQB1	HLA-DQB1	DOWN	0
316	1992,116291	1,780393486	0,164210445	10,84214521	2,17315E-27	2,67716E-25	AOX1	AOX1	UP	1
3162	11232,28565	-1,155768686	0,085393648	-13,53459793	9,77156E-42	2,17828E-39	HMOX1	HMOX1	DOWN	0
3175	7,708854112	21,72517819	3,337862258	6,508710218	7,57988E-11	2,46416E-09	ONECUT1	ONECUT1	UP	0
323	2197,168866	1,049386549	0,191231716	5,487513097	4,07632E-08	8,94497E-07	APBB2	APBB2	UP	1
3248	260,1127838	1,600688672	0,470286003	3,403649397	0,00066492	0,005305763	HPGD	HPGD	UP	0
33	480,1331255	1,669421234	0,297553032	5,610499821	2,01743E-08	4,60696E-07	ACADL	ACADL	UP	1
330	3273,414079	1,716355085	0,163148643	10,52019223	6,97312E-26	7,96183E-24	BIRC3	BIRC3	UP	1
3338	474,3945871	1,07631995	0,267086583	4,029854059	5,58115E-05	0,000622077	DNAJC4	DNAJC4	UP	1
3373	574,109448	1,132279445	0,32670992	3,465702682	0,000528848	0,004374051	HYAL1	HYAL1	UP	1
3383	1039,617051	1,199926227	0,302142436	3,97139257	7,14537E-05	0,000769552	ICAM1	ICAM1	UP	0
338322	20,25185509	7,003051183	2,200613513	3,182317632	0,001461015	0,010389111	NLRP10	NLRP10	UP	1
338761	68,02393587	8,118001111	1,818901661	4,463133596	8,07697E-06	0,000111537	C1QL4	C1QL4	UP	1
339453	21,09048687	7,075037846	2,30300538	3,072089152	0,002125662	0,013982577	TMEM240	TMEM240	UP	1
340273	33,28727199	7,51968378	2,126368354	3,536397523	0,000405624	0,003466454	ABCB5	ABCB5	UP	1

ENTREZ	baseMean	log2FoldChange	lfcSE	stat	pvalue	padj	Symbol	delabel	diffexp	UNIQUE
340371	1503,35255	1,123584376	0,200091265	5,615359454	1,96154E-08	4,49394E-07	NRBP2	NRBP2	UP	1
341350	15,78960213	20,5892546	2,581217307	7,976567699	1,50459E-15	8,12708E-14	OVCH1	OVCH1	UP	0
342346	41,01995634	7,944857366	1,798410654	4,417710353	9,9752E-06	0,000133931	C16orf96	C16orf96	UP	1
342667	8813,238301	2,212960056	0,103671933	21,34579712	4,2663E-101	3,52443E-98	STAC2	STAC2	UP	1
344558	152,3631667	2,225793142	0,456778434	4,872806978	1,10024E-06	1,85495E-05	SH3RF3	SH3RF3	UP	1
344658	9,976984649	-19,69192528	3,230590928	-6,095456131	1,09126E-09	3,08363E-08	SAMD7	SAMD7	DOWN	0
345651	22,36138138	8,15578171	2,054886667	3,968969113	7,21842E-05	0,000776229	ACTBL2	ACTBL2	UP	1
347051	20,44586183	-6,171647039	2,241542131	-2,753304055	0,005899707	0,032092491	SLC10A5	SLC10A5	DOWN	0
347733	53,27460507	8,637880873	1,670695878	5,170229356	2,33807E-07	4,47355E-06	TUBB2B	TUBB2B	UP	1
3479	29,31329709	-8,97097321	1,762794849	-5,089062528	3,59838E-07	6,66697E-06	IGF1	IGF1	DOWN	1
347902	3692,680904	-1,13370646	0,102559169	-11,05416971	2,09264E-28	2,67173E-26	AMIGO2	AMIGO2	DOWN	0
3484	99,39625977	3,560162555	0,71406522	4,985766645	6,17166E-07	1,093E-05	IGFBP1	IGFBP1	UP	1
3554	398,8642446	1,476746333	0,523075804	2,823197557	0,004754726	0,026838978	IL1R1	IL1R1	UP	1
357	169,2449066	3,508788676	0,895207384	3,919526066	8,87233E-05	0,000934756	SHROOM2	SHROOM2	UP	0
3570	484,6332513	1,14383583	0,366054554	3,124768752	0,001779449	0,012140142	IL6R	IL6R	UP	1
3589	868,6907407	-2,860693681	0,332415887	-8,605767037	7,58081E-18	4,90622E-16	IL11	IL11	DOWN	0
3601	364,943585	-1,23592525	0,423083384	-2,921233254	0,003486487	0,020962351	IL15RA	IL15RA	DOWN	0
3613	4639,190369	1,241961069	0,130388313	9,525094993	1,64891E-21	1,37025E-19	IMPA2	IMPA2	UP	1
3623	66,40302087	2,910488785	0,925014951	3,146423508	0,001652805	0,011474044	INHA	INHA	UP	0
3672	1842,034655	1,511557126	0,160330101	9,427781273	4,18855E-21	3,38069E-19	ITGA1	ITGA1	UP	1
3676	170,1218576	-1,786610071	0,643586128	-2,776023277	0,005502827	0,030259084	ITGA4	ITGA4	DOWN	1
3678	27928,63746	1,578405985	0,066984747	23,56366282	9,0936E-123	1,161E-119	ITGA5	ITGA5	UP	1
3691	5867,654086	1,465048928	0,116805882	12,5425955	4,36462E-36	7,5675E-34	ITGB4	ITGB4	UP	1
3708	5471,794358	-1,87817218	0,242568104	-7,742865393	9,72009E-15	4,84074E-13	ITPR1	ITPR1	DOWN	1
3710	18905,05374	1,000638148	0,159216794	6,284752508	3,28376E-10	9,8541E-09	ITPR3	ITPR3	UP	1
374	641,7631004	2,204802228	0,309270876	7,129032834	1,01077E-12	4,05577E-11	AREG	AREG	UP	1
374393	3352,841628	1,108349993	0,136917916	8,094996085	5,72662E-16	3,22991E-14	FAM111B	FAM111B	UP	1
3752	36,83293467	4,123657923	1,404102104	2,936864714	0,003315487	0,02012217	KCND3	KCND3	UP	1
375704	14,46652151	7,948443056	2,318477996	3,428302132	0,000607369	0,004926718	ENHO	ENHO	UP	1
3759	44,46438014	-4,287833538	1,614859394	-2,655236458	0,007925283	0,040530518	KCNJ2	KCNJ2	DOWN	1
3784	905,3166693	1,994080579	0,26138402	7,628930714	2,36709E-14	1,12309E-12	KCNQ1	KCNQ1	UP	1
3851	38,10192697	5,914884129	2,126711025	2,781235467	0,005415244	0,029859322	KRT4	KRT4	UP	1
3853	22227,37292	6,916533751	0,652314026	10,60307378	2,88344E-26	3,43179E-24	KRT6A	KRT6A	UP	0

ENTREZ	baseMean	log2FoldChange	lfcSE	stat	pvalue	padj	Symbol	delabel	diffexp	UNIQUE
3860	556,8982185	1,35390279	0,418329092	3,23645383	0,001210248	0,008855984	KRT13	KRT13	UP	0
3868	896,4654655	1,226797857	0,341373024	3,593716463	0,000325995	0,002881226	KRT16	KRT16	UP	0
387509	1601,615648	3,463642032	0,302100443	11,46520009	1,97303E-30	2,77092E-28	GPR153	GPR153	UP	1
387700	48,51151097	8,065471584	1,7217386	4,684492514	2,80654E-06	4,34087E-05	SLC16A12	SLC16A12	UP	1
387718	23,08617117	6,515364206	2,514817735	2,590789828	0,009575595	0,047152756	TEX36	TEX36	UP	1
387758	33,86058189	6,642851894	1,786007008	3,719387363	0,000199707	0,00189122	FIBIN	FIBIN	UP	0
388121	1117,418381	3,144532256	0,304645474	10,32193985	5,60785E-25	5,87736E-23	TNFAIP8L3	TNFAIP8L3	UP	1
388341	98,50988997	2,564957425	0,920396068	2,786797461	0,005323174	0,029498287	LRRC75A	LRRC75A	UP	1
3885	89,47804913	2,702405424	0,981523925	2,753275141	0,005900229	0,032092491	KRT34	KRT34	UP	1
388610	1799,287371	1,881996634	0,252216448	7,46183149	8,5328E-14	3,8532E-12	TRNP1	TRNP1	UP	1
3887	266,7663357	1,538093494	0,515379527	2,984389978	0,002841444	0,017783082	KRT81	KRT81	UP	1
389136	1043,26435	1,376774877	0,209527819	6,570845272	5,00304E-11	1,66901E-09	VGLL3	VGLL3	UP	1
3892	296,6138401	1,715415663	0,493766919	3,474140527	0,000512493	0,004253809	KRT86	KRT86	UP	1
389692	189,2343605	5,038739664	0,782848192	6,436419882	1,22324E-10	3,83465E-09	MAFA	MAFA	UP	1
389792	1786,600323	-1,739790278	0,15247085	-11,41064192	3,69989E-30	5,14467E-28	IER5L	IER5L	DOWN	1
3932	15,02209258	7,852110332	2,921625538	2,687582727	0,007197127	0,037559231	LCK	LCK	UP	1
3934	39,54184187	8,184414737	1,978745448	4,13616352	3,53161E-05	0,000412905	LCN2	LCN2	UP	0
3936	31,11306129	-7,84313806	2,301779799	-3,407423274	0,000655793	0,005241868	LCP1	LCP1	DOWN	0
399474	562,0621212	1,929728551	0,476347662	4,051092734	5,0979E-05	0,000574137	TMEM200B	TMEM200B	UP	1
399818	386,8709804	-1,502355624	0,33925487	-4,42839811	9,49355E-06	0,000128447	EEF1AKMT2	EEF1AKMT2	DOWN	1
400499	13,41048479	6,926515057	2,550564768	2,715678953	0,006614002	0,03514455	LOC400499	LOC400499	UP	1
401024	57,08200073	-6,072099622	1,463523771	-4,148958658	3,33991E-05	0,00039513	FSIP2	FSIP2	DOWN	1
4015	8635,638982	1,905951433	0,066992647	28,4501588	4,8511E-178	1,1355E-174	LOX	LOX	UP	1
402573	14,9384808	-19,94745218	4,19235027	-4,758059537	1,95463E-06	3,1513E-05	SPACDR	SPACDR	DOWN	0
404217	773,6107626	-1,234628383	0,235698975	-5,238157627	1,62188E-07	3,23087E-06	CTXN1	CTXN1	DOWN	1
404550	383,4117163	1,074995903	0,315384434	3,408525558	0,00065315	0,005226686	C16orf74	C16orf74	UP	1
404734	17,49540871	-7,164169735	2,148266799	-3,334860334	0,000853423	0,006563782	ANKHD1-EIF4EBP3	ANKHD1-EIF4EBP3	DOWN	0
4066	36,06595517	-8,312887606	1,87415179	-4,435546604	9,1839E-06	0,000124738	LYL1	LYL1	DOWN	0
41	640,2067233	1,06129751	0,40814536	2,600292965	0,00931442	0,046160314	ASIC1	ASIC1	UP	1
4128	334,5226109	2,772122058	0,601990198	4,604928894	4,12607E-06	6,19087E-05	MAOA	MAOA	UP	1
4133	4088,881156	2,548927108	0,135334611	18,83425891	3,95626E-79	2,05784E-76	MAP2	MAP2	UP	1
4222	314,2155729	-1,988136431	0,627632547	-3,1676758	0,001536628	0,010828098	MEOX1	MEOX1	DOWN	1
4240	2193,993043	2,353417806	0,264012156	8,91405095	4,92012E-19	3,40385E-17	MFGE8	MFGE8	UP	1

ENTREZ	baseMean	log2FoldChange	lfcSE	stat	pvalue	padj	Symbol	delabel	diffexp	UNIQUE
4354	442,9820513	2,276542357	0,320323985	7,106999355	1,18593E-12	4,67843E-11	MPP1	MPP1	UP	1
4355	1680,417422	1,078827532	0,266759054	4,044202123	5,25016E-05	0,000589395	MPP2	MPP2	UP	1
440345	70,09323106	-2,130428827	0,793256598	-2,685674261	0,007238362	0,037706066	NPIPB4	NPIPB4	DOWN	1
442117	47,93243737	-3,777168279	1,210515042	-3,120298509	0,001806679	0,012275275	GALNTL6	GALNTL6	DOWN	1
442590	12,71930003	-8,733867434	2,694775853	-3,241036698	0,001190959	0,008750965	SPDYE5	SPDYE5	DOWN	0
4493	9331,61303	1,317095993	0,109681664	12,00835171	3,21183E-33	5,24499E-31	MT1E	MT1E	UP	1
4495	17,19753013	7,146676002	2,324785196	3,074123155	0,002111223	0,013907134	MT1G	MT1G	UP	1
4501	507,1661416	2,395665518	0,486742807	4,921830346	8,57385E-07	1,47022E-05	MT1X	MT1X	UP	1
4502	35708,60125	2,385222558	0,087275985	27,32965514	1,8848E-164	3,7815E-161	MT2A	MT2A	UP	1
4582	3986,710852	2,474325931	0,152130832	16,2644607	1,76414E-59	7,50773E-57	MUC1	MUC1	UP	0
4600	115,727894	-6,055918874	2,251737717	-2,689442394	0,00715715	0,037421822	MX2	MX2	DOWN	0
4621	15,75387978	7,377744246	2,475880235	2,979846982	0,002883924	0,018000813	MYH3	MYH3	UP	1
4633	13,67893096	6,979748118	2,513835203	2,776533685	0,005494194	0,030235291	MYL2	MYL2	UP	1
4640	14,87257589	7,646617481	2,594663852	2,947055156	0,00320816	0,019597823	MYO1A	MYO1A	UP	1
4647	33,99599041	7,914637885	1,785400171	4,432976994	9,29408E-06	0,000125869	MYO7A	MYO7A	UP	1
477	30,17393625	6,242098262	2,111916149	2,955656296	0,003120046	0,019142823	ATP1A2	ATP1A2	UP	1
4773	476,6072164	-1,234698537	0,38153752	-3,23611302	0,001211694	0,008858423	NFATC2	NFATC2	DOWN	1
4810	558,8252381	-1,028886059	0,354202672	-2,904794744	0,00367494	0,021896843	NHS	NHS	DOWN	1
4811	1122,922623	1,235275256	0,274580094	4,498779343	6,83447E-06	9,61757E-05	NID1	NID1	UP	0
482	38,84875059	8,362981776	1,848801273	4,523461715	6,08363E-06	8,70933E-05	ATP1B2	ATP1B2	UP	1
4837	3530,27265	1,512019047	0,130699663	11,56865298	5,9408E-31	8,6909E-29	NNMT	NNMT	UP	1
4854	4315,287664	1,087383407	0,146061308	7,444705414	9,7161E-14	4,34563E-12	NOTCH3	NOTCH3	UP	1
4856	1435,119238	-1,148707192	0,202574692	-5,670536527	1,42351E-08	3,32089E-07	CCN3	CCN3	DOWN	1
489	4952,697995	1,547400243	0,119674915	12,93003004	3,047E-38	5,86192E-36	ATP2A3	ATP2A3	UP	1
4897	178,4086496	2,486597692	0,654538913	3,799006663	0,000145277	0,001440871	NRCAM	NRCAM	UP	1
4899	823,3218392	-1,202400206	0,287204316	-4,18656733	2,83205E-05	0,000338785	NRF1	NRF1	DOWN	1
4973	984,231272	-1,041540091	0,300480676	-3,466246498	0,000527779	0,004367785	OLR1	OLR1	DOWN	0
4982	701,7286947	-1,662844375	0,330936656	-5,0246606	5,04324E-07	9,1508E-06	TNFRSF11B	TNFRSF11B	DOWN	1
5054	11051,83961	1,574770535	0,076279096	20,64485061	1,08598E-94	7,62573E-92	SERPINE1	SERPINE1	UP	0
50617	88,35738291	2,638186581	0,830840238	3,175323558	0,001496695	0,010589212	ATP6V0A4	ATP6V0A4	UP	1
50651	90,23904978	5,143416585	1,028020913	5,003221742	5,638E-07	1,00866E-05	SLC45A1	SLC45A1	UP	1
50848	555,7863371	1,51535089	0,350172894	4,327436297	1,50855E-05	0,000192951	F11R	F11R	UP	1
5101	126,1797536	3,070758507	0,777585061	3,949096583	7,84467E-05	0,000836527	PCDH9	PCDH9	UP	1

ENTREZ	baseMean	log2FoldChange	lfcSE	stat	pvalue	padj	Symbol	delabel	diffexp	UNIQUE
51110	468,6358198	1,016187702	0,326420148	3,113128	0,001851157	0,012516924	LACTB2	LACTB2	UP	1
51129	1163,698171	3,705545892	0,277835839	13,33717746	1,40686E-40	2,99363E-38	ANGPTL4	ANGPTL4	UP	0
51147	528,0729812	1,021322973	0,384557058	2,655842485	0,007911056	0,040501257	ING4	ING4	UP	1
51161	383,9011022	1,37063176	0,387147526	3,540334549	0,00039962	0,003420027	C3orf18	C3orf18	UP	1
51196	1477,563901	1,727414282	0,212702047	8,121286571	4,61267E-16	2,63335E-14	PLCE1	PLCE1	UP	1
51334	134,3828951	-2,130048715	0,634983143	-3,354496475	0,000795096	0,006193191	PRR16	PRR16	DOWN	1
5145	27,36815257	8,382901739	2,199698623	3,81093194	0,000138444	0,001382863	PDE6A	PDE6A	UP	1
51463	162,6323896	1,467908287	0,571804479	2,567150734	0,010253803	0,049742455	GPR89B	GPR89B	UP	1
51513	36,32404751	7,576817791	2,042136838	3,710240004	0,000207063	0,001950363	ETV7	ETV7	UP	0
51560	262,7905252	1,194406989	0,436773393	2,734614808	0,006245331	0,033630917	RAB6B	RAB6B	UP	1
5159	284,6560913	1,557911303	0,494971009	3,147479901	0,001646844	0,011438319	PDGFRB	PDGFRB	UP	1
5166	7040,326072	2,649473165	0,104206198	25,42529346	1,3248E-142	2,0672E-139	PDK4	PDK4	UP	0
5167	810,5199913	1,597054544	0,297915445	5,360764515	8,28705E-08	1,72676E-06	ENPP1	ENPP1	UP	0
51673	32,36316487	7,115878345	2,021301937	3,520443044	0,000430827	0,003658118	TPPP3	TPPP3	UP	0
5187	5575,558651	3,068679568	0,096104726	31,93057932	1,0052E-223	2,8234E-220	PER1	PER1	UP	1
5210	748,1073143	-1,736743278	0,280207443	-6,198062612	5,71624E-10	1,67407E-08	PFKFB4	PFKFB4	DOWN	1
5270	8101,397142	-1,292601893	0,129257005	-10,00024637	1,52018E-23	1,49297E-21	SERPINE2	SERPINE2	DOWN	0
5272	354,952696	1,132616023	0,332139181	3,410064476	0,000649475	0,005200868	SERPINB9	SERPINB9	UP	1
5295	1337,741144	1,293857878	0,208324046	6,210794699	5,27173E-10	1,55212E-08	PIK3R1	PIK3R1	UP	1
5296	6,201577351	-28,69532632	4,922600402	-5,829302396	5,56596E-09	1,39875E-07	PIK3R2	PIK3R2	DOWN	0
5318	680,6114623	1,333195637	0,331938373	4,016395042	5,90952E-05	0,00065349	PKP2	PKP2	UP	1
5324	41,86874855	3,60803827	1,297761712	2,780200892	0,005432528	0,029931119	PLAG1	PLAG1	UP	1
5328	148,381716	-1,619555813	0,596230515	-2,71632493	0,00660111	0,035139056	PLAU	PLAU	DOWN	1
5362	3564,568083	1,470743022	0,15760796	9,331654454	1,04231E-20	8,22371E-19	PLXNA2	PLXNA2	UP	0
5365	190,9992657	1,738121265	0,677398692	2,565876323	0,010291552	0,049891112	PLXNB3	PLXNB3	UP	1
5372	1501,530404	1,168347179	0,179268562	6,51730102	7,15837E-11	2,33796E-09	PMM1	PMM1	UP	1
53833	522,1804108	2,143424081	0,698617039	3,068095912	0,002154275	0,014137679	IL20RB	IL20RB	UP	1
53836	62,92940621	3,286119188	1,053093186	3,120444829	0,001805781	0,012275117	GPR87	GPR87	UP	1
5409	456,9466845	11,73540005	1,643223436	7,141694668	9,21872E-13	3,72034E-11	PNMT	PNMT	UP	1
54206	5423,043097	1,978230222	0,085055445	23,25812558	1,1772E-119	1,2717E-116	ERRFI1	ERRFI1	UP	1
54436	2096,708665	1,970821309	0,203944635	9,663511439	4,30838E-22	3,68944E-20	SH3TC1	SH3TC1	UP	1
5445	8521,795122	1,351737838	0,07268906	18,59616627	3,45124E-77	1,73104E-74	PON2	PON2	UP	1
54491	3715,025965	1,272521532	0,116382413	10,9339676	7,93035E-28	9,94409E-26	OTULINL	OTULINL	UP	1

ENTREZ	baseMean	log2FoldChange	lfcSE	stat	pvalue	padj	Symbol	delabel	diffexp	UNIQUE
54535	1804,660396	1,040887578	0,188489349	5,522262057	3,34663E-08	7,37834E-07	CCHCR1	CCHCR1	UP	1
54541	6778,798693	2,121395442	0,157236131	13,491781	1,74826E-41	3,83633E-39	DDIT4	DDIT4	UP	1
54577	22,15214061	6,703030568	2,260523923	2,965255311	0,003024318	0,018717994	UGT1A7	UGT1A7	UP	1
54831	1827,884626	13,96200447	1,669754882	8,36170903	6,18162E-17	3,71003E-15	BEST2	BEST2	UP	1
54858	413,3386231	1,520180089	0,480477789	3,163892535	0,001556743	0,010936915	PGPEP1	PGPEP1	UP	1
5493	14217,59358	2,499508055	0,114690917	21,79342628	2,6784E-105	2,351E-102	PPL	PPL	UP	1
54946	3068,090621	1,226438403	0,13365712	9,176004991	4,47382E-20	3,35991E-18	SLC41A3	SLC41A3	UP	1
55062	227,8765069	1,236471916	0,47015026	2,62995051	0,00853973	0,042955576	WIPI1	WIPI1	UP	1
55089	91,55394373	5,304239366	0,953934912	5,560378698	2,6919E-08	6,0488E-07	SLC38A4	SLC38A4	UP	0
55112	698,9473112	1,186509896	0,293539672	4,042076794	5,29799E-05	0,000593814	DYNC2I1	DYNC2I1	UP	1
55130	287,9787724	-1,085882482	0,376013303	-2,887883151	0,003878439	0,022847651	ODAD2	ODAD2	DOWN	1
55222	675,2455857	1,114230605	0,266091441	4,187397387	2,82172E-05	0,000337836	LRRC20	LRRC20	UP	1
55224	1590,995007	3,618740607	0,235436859	15,37032319	2,58875E-53	9,56746E-51	ETNK2	ETNK2	UP	1
55273	35,48173046	4,202819585	1,559912497	2,694266245	0,007054379	0,037022309	TMEM100	TMEM100	UP	1
55301	100,6727648	10,38053782	2,095242396	4,954337426	7,25772E-07	1,27569E-05	OLAH	OLAH	UP	1
55332	1422,852638	-1,013319745	0,205195022	-4,93832518	7,87964E-07	1,36788E-05	DRAM1	DRAM1	DOWN	1
55351	614,7790622	-1,344988448	0,279708721	-4,808532396	1,52042E-06	2,50915E-05	STK32B	STK32B	DOWN	1
55356	198,7611187	2,267731822	0,598606454	3,788351772	0,00015165	0,001491438	SLC22A15	SLC22A15	UP	1
55367	500,5690081	1,999410851	0,388530075	5,146090305	2,65971E-07	5,04089E-06	PIDD1	PIDD1	UP	1
554	45,58319479	-4,230250987	1,281734248	-3,300411917	0,00096543	0,007289517	AVPR2	AVPR2	DOWN	1
55450	51,96792175	3,382381673	0,989794528	3,417256388	0,000632557	0,005100616	CAMK2N1	CAMK2N1	UP	0
55521	344,3676573	1,204969227	0,415021732	2,903388264	0,003691487	0,021967474	TRIM36	TRIM36	UP	1
55540	269,0693704	1,98564055	0,659341556	3,011550739	0,00259917	0,016569559	IL17RB	IL17RB	UP	1
55561	545,1568513	1,718052601	0,25639606	6,700776145	2,07315E-11	7,22466E-10	CDC42BPG	CDC42BPG	UP	0
55567	57,75639595	9,302846003	1,625098473	5,724481413	1,0375E-08	2,49071E-07	DNAH3	DNAH3	UP	1
55601	1484,392368	-1,566751038	0,215764438	-7,26139604	3,83115E-13	1,62552E-11	DDX60	DDX60	DOWN	0
55640	707,4121509	2,645014405	0,408860825	6,46922925	9,85041E-11	3,12278E-09	FLVCR2	FLVCR2	UP	1
55650	472,0845709	1,084878505	0,2416251	4,489924697	7,12484E-06	9,99612E-05	PIGV	PIGV	UP	1
55723	8336,219497	1,171145039	0,080774054	14,49902509	1,22883E-47	3,45153E-45	ASF1B	ASF1B	UP	1
55731	1460,667972	1,020548503	0,172104461	5,929820163	3,03267E-09	7,99076E-08	FAM222B	FAM222B	UP	1
55803	164,3553127	-2,714864489	0,692721697	-3,919127266	8,88702E-05	0,000935087	ADAP2	ADAP2	DOWN	1
55857	309,8554781	-1,139687849	0,440309995	-2,588376059	0,009642964	0,047417993	KIZ	KIZ	DOWN	1
5587	29,63714425	7,903643439	2,037654303	3,878795058	0,000104975	0,00108482	PRKD1	PRKD1	UP	1

ENTREZ	baseMean	log2FoldChange	lfcSE	stat	pvalue	padj	Symbol	delabel	diffexp	UNIQUE
55893	286,0468604	1,371668257	0,4102117	3,343805789	0,000826376	0,006383729	ZNF395	ZNF395	UP	1
55908	5,477506433	-31,54937858	4,848836508	-6,506587411	7,68772E-11	2,4877E-09	ANGPTL8	ANGPTL8	DOWN	0
5593	34,69957117	7,709302904	1,789708211	4,307575311	1,65054E-05	0,000208455	PRKG2	PRKG2	UP	0
5596	787,4227438	1,859675241	0,683893572	2,719246558	0,006543082	0,034935256	MAPK4	MAPK4	UP	1
55964	95,70692929	1,460019327	0,560108069	2,606674331	0,009142627	0,045483193	SEPTIN3	SEPTIN3	UP	1
56109	14,66620658	6,969496228	2,552445161	2,730517518	0,006323497	0,033960686	PCDHGA6	PCDHGA6	UP	1
56134	700,6269989	-1,349143528	0,262915361	-5,131474712	2,87481E-07	5,40479E-06	PCDHAC2	PCDHAC2	DOWN	1
56241	418,1036558	4,008898928	1,017325738	3,940624695	8,12697E-05	0,0008627	SUSD2	SUSD2	UP	1
5627	676,0885639	2,087365852	0,248351391	8,404888916	4,28267E-17	2,60371E-15	PROS1	PROS1	UP	1
56548	892,724658	2,699577203	0,336882649	8,013405302	1,11575E-15	6,10293E-14	CHST7	CHST7	UP	1
56704	1403,127735	1,114435116	0,189669465	5,875669647	4,21137E-09	1,08323E-07	JPH1	JPH1	UP	1
5675	16,96520321	6,264800365	2,305844695	2,71692208	0,006589212	0,035105801	PSG6	PSG6	UP	1
56892	3323,864091	-1,571657426	0,162694731	-9,660161794	4,4516E-22	3,78899E-20	TCIM	TCIM	DOWN	1
56917	543,0351983	1,388119156	0,238827201	5,812232228	6,16452E-09	1,54047E-07	MEIS3	MEIS3	UP	1
56928	1024,733519	1,04338938	0,284782674	3,6638092	0,000248492	0,002289908	SPPL2B	SPPL2B	UP	1
56952	153,3479182	1,414061467	0,507269499	2,787594109	0,005310103	0,029453035	PRTFDC1	PRTFDC1	UP	1
5698	350,9559315	-1,009248352	0,345994449	-2,916949551	0,003534729	0,021205357	PSMB9	PSMB9	DOWN	0
57016	127,4576244	2,647028472	0,92286178	2,868282692	0,004127066	0,023980354	AKR1B10	AKR1B10	UP	1
57139	160,8838957	1,37191239	0,475262249	2,886642889	0,003893759	0,022889892	RGL3	RGL3	UP	1
57149	843,0244658	1,180516936	0,282738475	4,1752964	2,97598E-05	0,000354794	LYRM1	LYRM1	UP	1
57156	635,7788673	2,519193833	0,241728815	10,42157026	1,97662E-25	2,13536E-23	TMEM63C	TMEM63C	UP	1
57211	3122,224484	1,179665973	0,166319486	7,092770658	1,31453E-12	5,11689E-11	ADGRG6	ADGRG6	UP	1
57216	12,16212948	-38,67226057	5,896639414	-6,558356015	5,44042E-11	1,80627E-09	VANGL2	VANGL2	DOWN	0
57224	321,6563557	1,33256066	0,429462415	3,102857462	0,001916619	0,012891284	NHSL1	NHSL1	UP	1
5732	214,3536272	2,550460517	0,617407746	4,13091759	3,61318E-05	0,00042041	PTGER2	PTGER2	UP	1
57335	295,620422	-1,2976599	0,433138638	-2,995945842	0,00273595	0,017261314	ZNF286A	ZNF286A	DOWN	1
5737	29,43797016	7,482309186	1,807662724	4,139217504	3,48492E-05	0,000408192	PTGFR	PTGFR	UP	0
5744	226,1999624	-1,857491321	0,511616146	-3,630634677	0,000282725	0,002555078	PTHLH	PTHLH	DOWN	0
57464	805,1359438	1,008589857	0,281672152	3,580722666	0,000342645	0,003003813	STRIP2	STRIP2	UP	1
57476	1898,943528	-1,105339478	0,189184412	-5,842656199	5,1375E-09	1,30472E-07	GRAMD1B	GRAMD1B	DOWN	1
57480	223,064984	1,774417895	0,472224587	3,757572022	0,00017157	0,001658313	PLEKHG1	PLEKHG1	UP	1
57523	459,3324908	1,979972632	0,340769929	5,810291525	6,23641E-09	1,54742E-07	NYNRIN	NYNRIN	UP	1
57535	196,7951477	-1,280102376	0,489343639	-2,615957935	0,008897752	0,044485592	ELAPOR1	ELAPOR1	DOWN	1

ENTREZ	baseMean	log2FoldChange	lfcSE	stat	pvalue	padj	Symbol	delabel	diffexp	UNIQUE
57568	1695,211275	1,253293264	0,219415109	5,711973397	1,11674E-08	2,66272E-07	SIPA1L2	SIPA1L2	UP	1
57595	270,7920303	2,527658831	0,664783462	3,802228809	0,0001434	0,001424266	PDZD4	PDZD4	UP	1
57596	3044,085082	-1,426842992	0,122287352	-11,66795233	1,85843E-31	2,80642E-29	BEGAIN	BEGAIN	DOWN	1
57608	16,42040129	7,364283949	2,579617017	2,854797399	0,004306429	0,02480701	JCAD	JCAD	UP	1
57624	89,1845088	-2,326339008	0,901583587	-2,580281008	0,009871995	0,048290593	NYAP2	NYAP2	DOWN	0
57669	341,3873809	1,044339377	0,339907241	3,072424626	0,002123274	0,013973415	EPB41L5	EPB41L5	UP	1
57822	16,43065122	-17,58739947	2,510506261	-7,005519064	2,46072E-12	9,14241E-11	GRHL3	GRHL3	DOWN	0
58476	850,0364041	1,823592402	0,311364058	5,85678519	4,71913E-09	1,2072E-07	TP53INP2	TP53INP2	UP	1
58500	341,7610108	2,011747852	0,635430598	3,165959992	0,001545721	0,010875801	ZNF250	ZNF250	UP	1
58516	2296,125716	-1,134581637	0,159274191	-7,123449368	1,05259E-12	4,1877E-11	SINHCAF	SINHCAF	DOWN	1
5873	1699,544101	1,03447961	0,185015264	5,591320356	2,25349E-08	5,12105E-07	RAB27A	RAB27A	UP	1
5920	96,08924508	-8,447909945	1,528980161	-5,525192649	3,29124E-08	7,26764E-07	PLAAT4	PLAAT4	DOWN	0
59344	38,69649928	5,72252351	1,876040434	3,050319921	0,002285977	0,014900091	ALOXE3	ALOXE3	UP	1
59352	381,0398772	-1,505791088	0,520753981	-2,891559434	0,003833351	0,022629499	LGR6	LGR6	DOWN	1
5970	59971,95526	1,01507972	0,261050053	3,888448633	0,000100887	0,001047971	RELA	RELA	UP	0
5971	706,3159953	-1,478742129	0,321764769	-4,595724173	4,31249E-06	6,43619E-05	RELB	RELB	DOWN	0
5973	15,81389371	7,971655511	2,663367791	2,993073483	0,002761832	0,017394634	RENBP	RENBP	UP	1
598	4770,550766	1,041995408	0,129316216	8,05773196	7,7723E-16	4,3144E-14	BCL2L1	BCL2L1	UP	1
5990	542,1197627	1,100282308	0,309766637	3,551971632	0,000382356	0,003296384	RFX2	RFX2	UP	1
5997	3781,938716	2,304412145	0,132536653	17,38698007	1,03548E-67	4,84745E-65	RGS2	RGS2	UP	0
6004	130,5229155	-1,74456736	0,622361368	-2,803142113	0,005060736	0,02833851	RGS16	RGS16	DOWN	1
60680	14,51174059	7,260090446	2,591449844	2,801555455	0,00508569	0,028466891	CELF5	CELF5	UP	1
6094	289,285057	1,292422936	0,426759779	3,028455352	0,002458074	0,015806406	ROM1	ROM1	UP	1
6097	128,1540383	2,349844878	0,810605995	2,89887429	0,00374505	0,022210931	RORC	RORC	UP	1
622	1133,806713	1,490745691	0,290726758	5,127652168	2,93378E-07	5,50093E-06	BDH1	BDH1	UP	1
6236	130,6293577	3,565986505	1,099827864	3,242313294	0,001185636	0,008717839	RRAD	RRAD	UP	0
6252	89,20487779	2,816125064	0,907095461	3,104552041	0,001905674	0,012834841	RTN1	RTN1	UP	1
6258	19,92552843	7,058212149	2,397336061	2,944189704	0,003238015	0,019758721	RXRG	RXRG	UP	1
6261	14,19582937	6,862289314	2,596931629	2,642460524	0,008230606	0,04179045	RYR1	RYR1	UP	1
6286	1931,65246	1,832476845	0,175327298	10,45174861	1,43848E-25	1,56605E-23	S100P	S100P	UP	1
6288	519,6291961	4,589304755	1,027647955	4,46583359	7,97576E-06	0,000110356	SAA1	SAA1	UP	1
6303	1641,737602	1,459199643	0,169212252	8,623486916	6,49456E-18	4,24231E-16	SAT1	SAT1	UP	1
6310	791,9457864	-1,005994904	0,343865217	-2,925550053	0,003438479	0,020743124	ATXN1	ATXN1	DOWN	1

ENTREZ	baseMean	log2FoldChange	lfcSE	stat	pvalue	padj	Symbol	delabel	diffexp	UNIQUE
6337	5348,694455	4,930669468	0,11648042	42,33045733	0	0	SCNN1A	SCNN1A	UP	1
6345	54,57106287	8,56708274	1,593539521	5,376134466	7,6102E-08	1,60236E-06	SRL	SRL	UP	1
638	30,63036633	6,731551794	2,041234754	3,297784236	0,00097451	0,007346225	BIK	BIK	UP	0
6382	3988,587837	1,148755535	0,104759554	10,96563984	5,59037E-28	7,07308E-26	SDC1	SDC1	UP	1
63924	21,82085752	7,312066653	2,204824539	3,316393901	0,000911872	0,006948633	CIDEC	CIDEC	UP	1
64005	1447,853821	1,939022986	0,197763406	9,804761281	1,07401E-22	9,66881E-21	MYO1G	MYO1G	UP	0
64061	2896,121397	1,6345679	0,163043036	10,025377	1,17909E-23	1,17013E-21	TSPYL2	TSPYL2	UP	1
64093	11442,6247	-1,063582938	0,066704334	-15,94473519	3,09951E-57	1,28028E-54	SMOC1	SMOC1	DOWN	1
64097	1649,488825	1,437629801	0,220563549	6,517984536	7,12583E-11	2,33276E-09	EPB41L4A	EPB41L4A	UP	1
64115	784,3245211	1,627531391	0,33564946	4,84890216	1,24147E-06	2,08057E-05	VSIR	VSIR	UP	1
64137	11,28413261	7,188306345	2,764440148	2,600275629	0,009314891	0,046160314	ABCG4	ABCG4	UP	1
64220	127,7396509	4,282272898	0,900668353	4,7545502	1,98889E-06	3,19953E-05	STRA6	STRA6	UP	1
64288	444,4736413	-1,592643442	0,373090588	-4,268784832	1,96541E-05	0,00024362	ZSCAN31	ZSCAN31	DOWN	1
643338	27,16635454	3,595702144	1,28085291	2,807271714	0,004996308	0,028044823	C15orf62	C15orf62	UP	0
64377	438,2737672	-1,240543038	0,361725681	-3,429513308	0,000604665	0,004914301	CHST8	CHST8	DOWN	1
64386	17,24696906	7,933264111	2,911767127	2,724553086	0,006438858	0,034487917	MMP25	MMP25	UP	1
6453	4958,627405	1,09625212	0,142730651	7,680565553	1,58388E-14	7,64398E-13	ITSN1	ITSN1	UP	1
647024	2297,247049	1,445077838	0,207703625	6,957403058	3,46602E-12	1,26433E-10	C6orf132	C6orf132	UP	1
6474	202,9895526	2,213724068	0,622889696	3,553958404	0,000379479	0,003277618	SHOX2	SHOX2	UP	1
64753	85,28156523	1,981826264	0,596342489	3,323302129	0,000889585	0,006804648	CCDC136	CCDC136	UP	1
64762	518,7527996	1,760734315	0,395831599	4,448190392	8,65968E-06	0,000118538	GAREM1	GAREM1	UP	1
64780	2044,018804	1,035468461	0,130313969	7,945951349	1,92706E-15	1,02904E-13	MICAL1	MICAL1	UP	1
6482	13121,46137	1,460470498	0,096066251	15,20274273	3,3912E-52	1,16161E-49	ST3GAL1	ST3GAL1	UP	1
64840	1095,963715	1,333924428	0,219747316	6,070264945	1,27699E-09	3,56543E-08	PORCN	PORCN	UP	0
64900	696,429782	1,318213663	0,229862806	5,73478452	9,76364E-09	2,36125E-07	LPIN3	LPIN3	UP	1
6493	1174,269251	-1,363267355	0,293716713	-4,641436107	3,45996E-06	5,25884E-05	SIM2	SIM2	DOWN	1
6494	2772,296911	1,057047795	0,190997253	5,534361245	3,12365E-08	6,94122E-07	SIPA1	SIPA1	UP	1
65055	1126,189875	1,994742306	0,24908655	8,008229708	1,16371E-15	6,33457E-14	REEP1	REEP1	UP	1
6518	399,1031831	4,470335827	0,581408773	7,688800089	1,48521E-14	7,19253E-13	SLC2A5	SLC2A5	UP	1
65249	499,601899	-1,010835262	0,357170047	-2,830123272	0,004653007	0,02636156	ZSWIM4	ZSWIM4	DOWN	1
6526	569,2674496	-1,148804378	0,259472283	-4,427464726	9,53472E-06	0,000128879	SLC5A3	SLC5A3	DOWN	1
6531	22,66833293	8,187224421	2,530509784	3,235405163	0,001214702	0,008875796	SLC6A3	SLC6A3	UP	1
653145	1691,379936	1,039524149	0,169391104	6,136828479	8,41852E-10	2,40794E-08	ANXA8	ANXA8	UP	1

ENTREZ	baseMean	log2FoldChange	lfcSE	stat	pvalue	padj	Symbol	delabel	diffexp	UNIQUE
6533	3227,330046	-1,029800452	0,118738277	-8,672860029	4,214E-18	2,81816E-16	SLC6A6	SLC6A6	DOWN	1
653820	304,4339489	-1,505863964	0,411735214	-3,657360146	0,000254826	0,002337022	FAM72B	FAM72B	DOWN	1
6542	4207,740409	1,504575638	0,122983808	12,23393276	2,04779E-34	3,46495E-32	SLC7A2	SLC7A2	UP	1
656	515,6190669	1,07660916	0,362842639	2,96715172	0,003005726	0,018620386	BMP8B	BMP8B	UP	1
6560	4262,640432	1,014545792	0,094134979	10,77756437	4,39365E-27	5,36561E-25	SLC12A4	SLC12A4	UP	1
6578	2252,902712	1,774594357	0,185996171	9,541026267	1,41426E-21	1,18225E-19	SLCO2A1	SLCO2A1	UP	1
6584	8022,571615	1,331927153	0,097419937	13,67201825	1,49199E-42	3,55144E-40	SLC22A5	SLC22A5	UP	1
6591	1660,154827	1,32343767	0,181917787	7,27492176	3,46621E-13	1,48413E-11	SNAI2	SNAI2	UP	1
66004	20,05031765	6,849544367	2,447328063	2,798784711	0,005129533	0,028643799	LYNX1	LYNX1	UP	1
6662	712,6320525	-1,003062012	0,260214665	-3,854748204	0,000115849	0,001183259	SOX9	SOX9	DOWN	1
6833	307,8658268	11,6599538	1,332865926	8,748032021	2,17106E-18	1,46588E-16	ABCC8	ABCC8	UP	0
687	2256,89784	1,444020428	0,183607094	7,864731117	3,69891E-15	1,92398E-13	KLF9	KLF9	UP	1
6877	578,5044141	-1,024522993	0,337815738	-3,032786446	0,00242307	0,015631418	TAF5	TAF5	DOWN	1
6916	326,5337661	1,906063103	0,40484625	4,708115988	2,50017E-06	3,93636E-05	TBXAS1	TBXAS1	UP	1
6941	7384,582201	1,301270035	0,086234189	15,08995496	1,88571E-51	6,15882E-49	TCF19	TCF19	UP	1
70	1300,917022	-2,020021698	0,348329341	-5,799171812	6,66432E-09	1,642E-07	ACTC1	ACTC1	DOWN	0
7020	1612,230713	-1,26113738	0,157380754	-8,01328844	1,11681E-15	6,10293E-14	TFAP2A	TFAP2A	DOWN	1
7042	510,2187687	-1,364570584	0,313241333	-4,356291583	1,32285E-05	0,000171226	TGFB2	TGFB2	DOWN	1
7047	68,10143309	6,438240745	1,52664618	4,217244853	2,47305E-05	0,000302014	TGM4	TGM4	UP	1
7051	943,5548121	1,062124644	0,258283425	4,11224469	3,91831E-05	0,000451102	TGM1	TGM1	UP	1
7052	21112,27233	3,904856814	0,081540658	47,88846355	0	0	TGM2	TGM2	UP	0
7056	967,179456	1,688692449	0,236139001	7,151264476	8,59821E-13	3,48998E-11	THBD	THBD	UP	1
7079	853,8898304	1,607656634	0,251075698	6,403075436	1,52278E-10	4,74189E-09	TIMP4	TIMP4	UP	1
7097	27,1975454	7,473644677	2,131598608	3,506121954	0,000454687	0,003839821	TLR2	TLR2	UP	1
7103	525,7160505	-1,928369367	0,268176827	-7,190663661	6,44771E-13	2,65547E-11	TSPAN8	TSPAN8	DOWN	0
7139	495,8585219	2,312967958	0,741580023	3,11897285	0,001814827	0,01231429	TNNT2	TNNT2	UP	1
7145	1642,981712	1,001155533	0,220399041	4,542467745	5,55995E-06	8,03333E-05	TNS1	TNS1	UP	1
7161	545,1444312	2,230858339	0,401381937	5,557944026	2,72971E-08	6,12397E-07	TP73	TP73	UP	1
718	4069,80911	1,144890397	0,146225656	7,829613695	4,89371E-15	2,49917E-13	C3	C3	UP	0
727910	382,7614535	1,006223214	0,359196369	2,801317888	0,005089436	0,028476508	TLCD2	TLCD2	UP	0
728	192,0829527	2,763882711	0,884527816	3,124698466	0,001779874	0,012140142	C5AR1	C5AR1	UP	1
729238	17,63575197	17,93427372	5,142967692	3,487144932	0,000488207	0,004090916	SFTPA2	SFTPA2	UP	0
729920	94,53293037	3,289894903	0,867505088	3,792363812	0,00014922	0,001470629	CRPPA	CRPPA	UP	1

ENTREZ	baseMean	log2FoldChange	lfcSE	stat	pvalue	padj	Symbol	delabel	diffexp	UNIQUE
7450	16,84403064	6,220538193	2,312234686	2,690271118	0,007139399	0,037356826	VWF	VWF	UP	1
7477	10836,76426	-1,100175202	0,08588454	-12,80993296	1,44259E-37	2,66576E-35	WNT7B	WNT7B	DOWN	1
7481	152,2816475	2,769316755	0,574473539	4,820616732	1,43115E-06	2,37859E-05	WNT11	WNT11	UP	0
7516	763,8100034	1,074007968	0,260701982	4,119677041	3,79404E-05	0,000438908	XRCC2	XRCC2	UP	1
7538	1171,46066	1,875009342	0,254347438	7,371842841	1,68285E-13	7,36261E-12	ZFP36	ZFP36	UP	1
7552	34,44312769	4,728940021	1,804811152	2,620185505	0,008788195	0,043953492	ZNF711	ZNF711	UP	0
7678	183,3737697	-1,663746838	0,615992167	-2,700922068	0,006914754	0,036412	ZNF124	ZNF124	DOWN	1
7704	113,1753909	9,346427351	1,402322368	6,664963467	2,64731E-11	9,13485E-10	ZBTB16	ZBTB16	UP	1
7803	42,1580992	6,509634294	1,453682802	4,478029378	7,53352E-06	0,000104857	PTP4A1	PTP4A1	UP	0
7832	804,8500063	1,444055007	0,300833411	4,800181611	1,58522E-06	2,60994E-05	BTG2	BTG2	UP	0
78990	66,06423859	3,135393644	1,080240151	2,902496857	0,003702009	0,022011436	OTUB2	OTUB2	UP	1
78991	440,4412889	1,120859011	0,358145184	3,129621904	0,001750314	0,011985087	PCYOX1L	PCYOX1L	UP	1
79056	512,7534254	2,039535529	0,360624933	5,655558848	1,5534E-08	3,60594E-07	PRRG4	PRRG4	UP	1
79078	114,9794078	1,514293364	0,497616146	3,043095321	0,002341581	0,015207144	C1orf50	C1orf50	UP	1
79094	212,9863925	1,57448212	0,519210465	3,032454516	0,002425737	0,015641434	CHAC1	CHAC1	UP	1
79154	750,8865394	1,005649365	0,249909434	4,024055229	5,72045E-05	0,000635083	DHRS11	DHRS11	UP	1
79173	147,9384602	3,147313363	0,771102953	4,081573477	4,47318E-05	0,000510743	BRME1	BRME1	UP	1
79652	9,5133045	-38,84919851	5,903574191	-6,580623408	4,6848E-11	1,574E-09	TMEM204	TMEM204	DOWN	0
79689	603,2932503	1,782643744	0,328381518	5,428575139	5,68057E-08	1,21613E-06	STEAP4	STEAP4	UP	0
79729	192,4294307	2,116817298	0,709528851	2,983412576	0,002850535	0,017824091	SH3D21	SH3D21	UP	1
79745	856,5838131	-1,213387605	0,276205938	-4,393054022	1,11769E-05	0,0001473	CLIP4	CLIP4	DOWN	1
79751	3227,580266	-1,013340737	0,156791253	-6,462992793	1,02652E-10	3,24695E-09	SLC25A22	SLC25A22	DOWN	1
79762	128,6396612	1,94449551	0,726524341	2,676435463	0,007440989	0,038575584	C1orf115	C1orf115	UP	1
79772	455,7682306	1,162077405	0,368784532	3,151101266	0,001626561	0,011325444	MCTP1	MCTP1	UP	1
79774	393,9677483	1,223923423	0,420817342	2,908443406	0,003632329	0,021688959	GRTP1	GRTP1	UP	1
79846	37,58991144	-4,774640828	1,524042452	-3,132879154	0,001731007	0,011870245	CFAP69	CFAP69	DOWN	1
79897	51,61979118	-2,574415334	0,968200154	-2,658970176	0,00783799	0,040203336	RPP21	RPP21	DOWN	1
79937	310,6381516	1,374559163	0,408310659	3,366454276	0,000761412	0,005973177	CNTNAP3	CNTNAP3	UP	1
79961	392,7593904	2,186490699	0,778496044	2,80860862	0,004975609	0,027962169	DENND2D	DENND2D	UP	1
79987	225,7997623	2,452829705	0,663282625	3,69801592	0,000217291	0,002038503	SVEP1	SVEP1	UP	1
8000	1339,369011	2,726317735	0,258066281	10,56440898	4,35705E-26	5,0992E-24	PSCA	PSCA	UP	1
80000	1225,828343	1,397396587	0,181256312	7,709505789	1,26306E-14	6,19591E-13	GREB1L	GREB1L	UP	1
80019	799,0624076	1,178782138	0,280890849	4,196584333	2,70971E-05	0,000326093	UBTD1	UBTD1	UP	1

ENTREZ	baseMean	log2FoldChange	lfcSE	stat	pvalue	padj	Symbol	delabel	diffexp	UNIQUE
80022	258,8344479	-1,413686055	0,449661403	-3,14389015	0,00166718	0,011536775	MYO15B	MYO15B	DOWN	0
80031	17,92992552	7,323435034	2,674097462	2,738656739	0,006169075	0,033322494	SEMA6D	SEMA6D	UP	1
8013	640,4918281	-1,274562531	0,330163651	-3,860396287	0,000113203	0,001159611	NR4A3	NR4A3	DOWN	1
80223	2275,334904	1,395883974	0,199023597	7,01366067	2,32162E-12	8,71787E-11	RAB11FIP1	RAB11FIP1	UP	1
80237	21,1666522	7,63436438	2,347314452	3,252382471	0,001144419	0,008459064	ELL3	ELL3	UP	0
80264	414,6099256	1,136960201	0,30878919	3,681994825	0,000231416	0,002147326	ZNF430	ZNF430	UP	1
80323	398,1505638	1,986461109	0,603038494	3,294086743	0,00098742	0,0074276	CCDC68	CCDC68	UP	1
80328	371,2883026	1,208439828	0,286365755	4,219917382	2,44392E-05	0,000298715	ULBP2	ULBP2	UP	1
8061	2197,015716	-1,022855677	0,181547234	-5,63410225	1,75973E-08	4,07143E-07	FOSL1	FOSL1	DOWN	0
80709	284,6025741	1,421798745	0,435571091	3,264217424	0,001097669	0,008147813	AKNA	AKNA	UP	1
80726	48,67825376	3,798379665	1,21186907	3,134315216	0,001722557	0,011829629	IQCN	IQCN	UP	1
8076	6255,152492	1,238519224	0,107678906	11,501967	1,28943E-30	1,82917E-28	MFAP5	MFAP5	UP	1
80820	369,6163464	1,516985344	0,346155376	4,382382732	1,17388E-05	0,000153644	EEPD1	EEPD1	UP	1
814	631,8971161	1,157597873	0,295597694	3,916126194	8,99832E-05	0,000943782	CAMK4	CAMK4	UP	1
81491	21,79903953	7,445168922	2,077714565	3,583345397	0,000339222	0,002980183	GPR63	GPR63	UP	1
81563	1251,883913	1,51627383	0,310122654	4,889271421	1,0121E-06	1,71666E-05	C1orf21	C1orf21	UP	1
81607	385,638537	2,86355846	0,553209193	5,176266944	2,2637E-07	4,35498E-06	NECTIN4	NECTIN4	UP	0
81618	3974,641609	1,052595612	0,105696582	9,958653256	2,31173E-23	2,20857E-21	ITM2C	ITM2C	UP	1
81928	550,8424041	1,296802525	0,333430749	3,889270949	0,000100546	0,0010452	CABLES2	CABLES2	UP	1
8193	753,0102917	1,525617462	0,40223606	3,792841106	0,000148933	0,001468835	DPF1	DPF1	UP	1
8291	65,28866392	-2,962778711	0,950038901	-3,118586731	0,001817206	0,012322959	DYSF	DYSF	DOWN	0
8332	13,33421915	7,294763785	2,790759796	2,613898837	0,008951554	0,044706835	H2AC16	H2AC16	UP	1
834	73,65356881	3,131860317	1,110342443	2,820625599	0,004793011	0,027044211	CASP1	CASP1	UP	0
8340	12,93052841	-8,093686978	2,329425662	-3,474541863	0,000511727	0,004249963	H2BC13	H2BC13	DOWN	1
83445	38,53226848	7,820512386	1,6663317	4,693250682	2,68898E-06	4,18206E-05	GSG1	GSG1	UP	1
83473	102,7855788	2,199588632	0,786183573	2,797805382	0,00514511	0,028707958	KATNAL2	KATNAL2	UP	1
83483	14,72474103	7,731353984	2,181530252	3,544004938	0,000394098	0,003385144	PLVAP	PLVAP	UP	0
83547	336,7551339	1,523034399	0,326622485	4,662980868	3,11662E-06	4,78882E-05	RILP	RILP	UP	1
83643	124,5234721	2,265369979	0,650640434	3,481754071	0,000498141	0,004156798	CCDC3	CCDC3	UP	1
8367	29,22688511	-8,218862905	2,038319156	-4,032176649	5,52626E-05	0,000617429	H4C5	H4C5	DOWN	0
83715	16,7408751	7,765878628	2,532856887	3,066055041	0,002169034	0,014227889	ESPN	ESPN	UP	1
83857	12157,95657	1,149351918	0,082942701	13,8571797	1,15109E-43	2,83612E-41	TMTC1	TMTC1	UP	1
83937	93,38283419	4,553181864	1,259398602	3,615362013	0,000299928	0,002689778	RASSF4	RASSF4	UP	1

ENTREZ	baseMean	log2FoldChange	lfcSE	stat	pvalue	padj	Symbol	delabel	diffexp	UNIQUE
84002	537,8438923	2,176318676	0,396502694	5,488786604	4,04704E-08	8,89463E-07	B3GNT5	B3GNT5	UP	1
84152	28,04817592	8,495664004	1,870570333	4,541750638	5,5789E-06	8,05242E-05	PPP1R1B	PPP1R1B	UP	0
84166	2041,563467	-1,172046095	0,171958288	-6,815874415	9,36921E-12	3,33962E-10	NLRC5	NLRC5	DOWN	1
84286	617,5630839	1,081235106	0,23055253	4,689755973	2,73531E-06	4,24472E-05	TMEM175	TMEM175	UP	1
84314	401,1516424	1,438724634	0,394472971	3,647207129	0,000265106	0,002413235	TMEM107	TMEM107	UP	1
8436	813,4313454	2,964632203	0,346288434	8,561164374	1,11733E-17	7,00528E-16	CAVIN2	CAVIN2	UP	1
84443	18,51667968	6,724875112	2,501120558	2,688744886	0,007172121	0,037486142	FRMPD3	FRMPD3	UP	1
84446	143,1440221	1,820731385	0,520827986	3,495840153	0,000472572	0,003976511	BRSK1	BRSK1	UP	0
84448	456,6287339	2,877683731	0,561532171	5,124699674	2,98012E-07	5,58038E-06	ABLIM2	ABLIM2	UP	1
84630	23,81651016	6,252220246	2,18149192	2,866029523	0,004156555	0,024111795	TTBK1	TTBK1	UP	1
84674	780,9941712	1,045678226	0,330496562	3,163960976	0,001556377	0,010936915	CARD6	CARD6	UP	1
8470	1711,889993	1,831967708	0,181685167	10,08319909	6,55563E-24	6,72023E-22	SORBS2	SORBS2	UP	1
84707	20,1402681	-6,832730362	2,525065531	-2,705961599	0,006810692	0,035985463	BEX2	BEX2	DOWN	1
8483	10,62886467	40,6346704	5,813535019	6,989666403	2,75541E-12	1,01834E-10	CILP	CILP	UP	0
84842	1127,902647	-1,272057954	0,257723732	-4,935742417	7,98464E-07	1,3844E-05	HPDL	HPDL	DOWN	1
84913	1706,845765	1,448370316	0,239006372	6,059965286	1,36151E-09	3,77141E-08	ATOH8	ATOH8	UP	1
84940	365,4189368	1,456700765	0,315701585	4,614169939	3,94669E-06	5,93441E-05	CORO6	CORO6	UP	1
84951	24547,34174	1,599203865	0,0831828	19,22517466	2,27871E-82	1,28009E-79	TNS4	TNS4	UP	1
84978	362,9355146	-1,902819548	0,610262199	-3,11803607	0,001820605	0,012340048	FRMD5	FRMD5	DOWN	1
84986	544,3539376	-1,019878467	0,332206607	-3,070012596	0,002140497	0,014053832	ARHGAP19	ARHGAP19	DOWN	1
85004	608,3086555	1,509526637	0,373950543	4,03670128	5,4208E-05	0,000606129	RERG	RERG	UP	1
8507	1428,263494	-1,054362453	0,189033012	-5,577663087	2,43771E-08	5,52181E-07	ENC1	ENC1	DOWN	1
8522	195,1738679	-2,414285509	0,869573871	-2,776400705	0,005496442	0,030235814	GAS7	GAS7	DOWN	0
8541	710,1434236	1,451777125	0,436730774	3,324192415	0,00088675	0,006786657	PPFIA3	PPFIA3	UP	1
8543	4660,649688	-1,177117464	0,128766616	-9,141480145	6,16033E-20	4,57755E-18	LMO4	LMO4	DOWN	1
85449	18,5346876	7,821270526	2,792932332	2,800379528	0,005104256	0,028548055	KIAA1755	KIAA1755	UP	1
85455	108,6365828	4,425395667	0,90723646	4,877885603	1,07229E-06	1,81218E-05	DISP2	DISP2	UP	1
85458	1749,081726	1,032812096	0,181387509	5,693953811	1,24131E-08	2,9299E-07	DIXDC1	DIXDC1	UP	1
8549	413,3458105	-2,22125995	0,548378314	-4,050597725	5,1087E-05	0,000574892	LGR5	LGR5	DOWN	1
8613	676,8994003	1,280459661	0,390376557	3,280062897	0,001037839	0,007754396	PLPP3	PLPP3	UP	0
8638	1336,769335	-1,672853	0,338032257	-4,948796947	7,46736E-07	1,30437E-05	OASL	OASL	DOWN	0
8645	548,7672137	1,053873847	0,304865047	3,456853636	0,000546522	0,004501671	KCNK5	KCNK5	UP	1
8651	173,8820895	2,547584063	0,783720529	3,250628212	0,001151503	0,008502478	SOCS1	SOCS1	UP	1

ENTREZ	baseMean	log2FoldChange	lfcSE	stat	pvalue	padj	Symbol	delabel	diffexp	UNIQUE
8685	35,1379131	8,350129673	2,105045302	3,966722078	7,28679E-05	0,000781594	MARCO	MARCO	UP	1
8701	174,5870134	2,310688423	0,551272835	4,1915514	2,77053E-05	0,000332843	DNAH11	DNAH11	UP	1
8710	91,92983545	1,834348891	0,688454128	2,664446062	0,007711523	0,039694097	SERPINB7	SERPINB7	UP	1
8736	18,87472294	7,387510357	2,43807076	3,030063966	0,002445019	0,015729663	MYOM1	MYOM1	UP	1
8739	421,3480354	3,294721007	0,413641998	7,965151071	1,65022E-15	8,87958E-14	HRK	HRK	UP	1
8740	7,770862314	40,45640909	3,757733332	10,76617352	4,9724E-27	6,02003E-25	TNFSF14	TNFSF14	UP	0
8796	675,2502013	6,19725886	1,169183339	5,300502202	1,15485E-07	2,34713E-06	SCEL	SCEL	UP	1
8858	12,08436161	-22,30632537	3,153997664	-7,072397555	1,52279E-12	5,8432E-11	PROZ	PROZ	DOWN	0
8878	27021,28362	-1,396521646	0,063466091	-22,00421699	2,6241E-107	2,6324E-104	SQSTM1	SQSTM1	DOWN	1
8900	55,94402637	4,155614638	1,286316593	3,230631292	0,001235172	0,008983299	CCNA1	CCNA1	UP	1
8913	159,5377258	2,192306674	0,738179474	2,969883005	0,002979132	0,018496431	CACNA1G	CACNA1G	UP	1
8974	7030,754358	1,077180058	0,115023909	9,364836094	7,61684E-21	6,04356E-19	P4HA2	P4HA2	UP	1
898	2437,088055	1,014730517	0,152809672	6,640486194	3,1265E-11	1,07356E-09	CCNE1	CCNE1	UP	1
89958	3584,253016	-1,260217372	0,148817539	-8,468204615	2,49204E-17	1,52831E-15	SAPCD2	SAPCD2	DOWN	1
9002	10,93152202	29,67824542	2,874552852	10,32447374	5,46175E-25	5,76728E-23	F2RL3	F2RL3	UP	1
9028	249,3203766	1,206552106	0,416949134	2,893763312	0,00380655	0,022509131	RHBDL1	RHBDL1	UP	1
90293	4392,774781	-1,160076789	0,139372828	-8,323550601	8,53668E-17	5,08005E-15	KLHL13	KLHL13	DOWN	1
90313	2333,364415	1,06623162	0,231608599	4,60359254	4,15265E-06	6,22409E-05	TP53I13	TP53I13	UP	1
9051	16,21378655	7,724711798	2,457158758	3,143757713	0,001667935	0,011536775	PSTPIP1	PSTPIP1	UP	1
9056	99,81326954	5,56688219	1,690651196	3,292744361	0,000992146	0,007451179	SLC7A7	SLC7A7	UP	1
9064	2664,071406	1,355911942	0,156595237	8,658704894	4,77154E-18	3,1759E-16	MAP3K6	MAP3K6	UP	1
90853	725,2399408	-1,148442931	0,214947584	-5,342897595	9,14724E-08	1,89475E-06	SPOCD1	SPOCD1	DOWN	0
9099	752,6576865	1,013783406	0,299082293	3,389647028	0,000699827	0,005534171	USP2	USP2	UP	0
90990	1030,811389	1,461908534	0,311040074	4,700064893	2,60079E-06	4,07731E-05	KIFC2	KIFC2	UP	1
91156	1129,606697	-1,373646408	0,312162975	-4,400414267	1,08044E-05	0,000143149	IGFN1	IGFN1	DOWN	1
9124	5435,745965	1,009387648	0,106385432	9,488025075	2,35453E-21	1,93374E-19	PDLIM1	PDLIM1	UP	1
9134	1164,457906	1,821650181	0,242043696	7,526121142	5,22698E-14	2,40681E-12	CCNE2	CCNE2	UP	1
91461	787,3357763	-1,651286031	0,287946345	-5,734700447	9,76848E-09	2,36125E-07	PKDCC	PKDCC	DOWN	1
91662	3,91861701	-24,19183739	5,89192694	-4,105929627	4,02692E-05	0,000462799	NLRP12	NLRP12	DOWN	0
9170	244,9641341	1,31283336	0,473437834	2,772979401	0,005554564	0,030461633	LPAR2	LPAR2	UP	1
9182	281,3176616	1,081993921	0,372376151	2,905647743	0,003664938	0,021846514	RASSF9	RASSF9	UP	1
9201	312,74992	3,235507315	0,475297207	6,807335004	9,94232E-12	3,53494E-10	DCLK1	DCLK1	UP	1
9232	4898,163964	-1,066152311	0,118762221	-8,977200849	2,77743E-19	1,96011E-17	PTTG1	PTTG1	DOWN	1

ENTREZ	baseMean	log2FoldChange	lfcSE	stat	pvalue	padj	Symbol	delabel	diffexp	UNIQUE
92370	966,1704457	1,903515431	0,306010028	6,220434802	4,95779E-10	1,46276E-08	PXYLP1	PXYLP1	UP	1
9245	98,27068281	5,197443422	1,128517088	4,605551373	4,11374E-06	6,17898E-05	GCNT3	GCNT3	UP	0
9249	617,9263012	1,399441582	0,320563392	4,365568928	1,26792E-05	0,00016503	DHRS3	DHRS3	UP	1
92558	337,5897644	1,187155055	0,361353446	3,285301606	0,001018733	0,007638591	BICDL1	BICDL1	UP	1
92714	1990,669832	1,004941361	0,126248789	7,960007916	1,72028E-15	9,22124E-14	ARRDC1	ARRDC1	UP	1
9315	2193,974644	1,114039007	0,145208991	7,67196987	1,69375E-14	8,14623E-13	NREP	NREP	UP	1
93233	10,2076927	32,10595411	5,350987873	6,000005022	1,97311E-09	5,35985E-08	ODAD1	ODAD1	UP	0
9340	3602,408928	-1,392757296	0,132821304	-10,48594807	1,00235E-25	1,09998E-23	GLP2R	GLP2R	DOWN	1
9362	9,643042429	20,19800739	3,229027871	6,255135662	3,97171E-10	1,18175E-08	CPNE6	CPNE6	UP	1
9363	12,20996269	18,82559506	5,742712898	3,27817103	0,001044821	0,007796738	RAB33A	RAB33A	UP	0
93649	287,6130935	-2,135585199	0,588411044	-3,629410461	0,000284069	0,002563926	MYOCD	MYOCD	DOWN	1
9399	1033,508694	1,014436174	0,212133493	4,782065102	1,73503E-06	2,83665E-05	STOML1	STOML1	UP	1
94234	2500,457057	2,600927832	0,21956955	11,84557616	2,26854E-32	3,5797E-30	FOXQ1	FOXQ1	UP	1
94240	26,18660009	7,649270452	2,171294512	3,522907837	0,00042684	0,00362646	EPSTI1	EPSTI1	UP	1
9495	218,871333	2,438670398	0,614711861	3,967176415	7,27292E-05	0,000780893	AKAP5	AKAP5	UP	1
9496	48,21009662	9,482575629	1,447362183	6,551625944	5,6914E-11	1,88514E-09	TBX4	TBX4	UP	0
9534	242,2986294	1,100437412	0,32705519	3,364684148	0,000766313	0,006002289	ZNF254	ZNF254	UP	1
9537	205,1978586	3,485216903	0,667806225	5,218904483	1,79985E-07	3,54517E-06	TP53I11	TP53I11	UP	1
9540	3712,980645	2,326875527	0,151830161	15,32551577	5,1643E-53	1,81319E-50	TP53I3	TP53I3	UP	1
9590	11171,78808	1,117833862	0,099444484	11,24078292	2,57092E-29	3,47173E-27	AKAP12	AKAP12	UP	1
9603	278,3286267	-1,661506386	0,490231838	-3,389225784	0,000700903	0,00553938	NFE2L3	NFE2L3	DOWN	0
9649	135,8900428	1,889834927	0,722816672	2,614542525	0,008934704	0,044638555	RALGPS1	RALGPS1	UP	0
9659	974,2815179	1,155886952	0,262502963	4,403329168	1,06602E-05	0,000141639	PDE4DIP	PDE4DIP	UP	1
9672	1375,572721	2,041954254	0,177146014	11,52695569	9,64959E-31	1,3971E-28	SDC3	SDC3	UP	1
9674	6970,463893	1,614175783	0,093175296	17,32407471	3,09636E-67	1,40275E-64	KIAA0040	KIAA0040	UP	1
9681	493,7945586	1,182036809	0,317757432	3,719934424	0,000199275	0,001888402	DEPDC5	DEPDC5	UP	1
9750	139,4262837	5,151271063	0,975196627	5,282289664	1,27579E-07	2,57802E-06	RIPOR2	RIPOR2	UP	1
9762	170,2190597	2,269103534	0,640563835	3,542353485	0,000396574	0,003400713	LZTS3	LZTS3	UP	1
9806	56,8347683	3,153693759	0,974939803	3,234757417	0,001217461	0,008891329	SPOCK2	SPOCK2	UP	1
9823	3626,466739	1,129147935	0,136423114	8,276808116	1,26521E-16	7,46579E-15	ARMCX2	ARMCX2	UP	1
9935	812,5170964	2,311409128	0,29624808	7,802275479	6,08007E-15	3,07153E-13	MAFB	MAFB	UP	1
9955	9,207881149	6,407582789	2,43995584	2,626106048	0,00863679	0,043366137	HS3ST3A1	HS3ST3A1	UP	1
9975	877,0109241	-1,608549405	0,28190707	-5,705956238	1,15692E-08	2,7492E-07	NR1D2	NR1D2	DOWN	1
