# Supplementary material for: The NF-κB Isoform p65 iso5 Is Associated with Distinct Transcriptional Programs and Signaling Pathways
Source: Cells. 2026 Sep 10;15(18):1643. doi: 10.3390/cells15181643 (PMC13605157; doi:10.3390/cells15181643)
Supplement: Supplementary file 1 [file cells-15-01643-s001.zip › TABLE S4.pdf]

ENTREZ	p65 iso5	p65 iso5_Dex	Dex	GFP_Dex	p65 iso5_Dex_vs_p65 iso5	Symbol
10002	1	1	0	1	1	NR2E3
1001	0	1	0	0	0	CDH3
100130311	1	1	0	1	0	C17orf107
100132247	0	1	0	0	0	NPIPB5
1002	0	0	0	0	1	CDH4
100381270	1	0	0	0	1	ZBED6
100419008	0	0	0	0	1	PRR20G
100533111	0	0	0	0	1	ZNF670-ZNF695
10076	0	0	0	0	1	PTPRU
10083	1	0	0	0	1	USH1C
1009	0	0	0	0	1	CDH11
10100	0	0	0	0	1	TSPAN2
101059915	0	0	0	0	1	LOC101059915
10107	1	0	0	0	1	TRIM10
10125	0	0	0	0	1	RASGRP1
10129	0	0	0	0	1	FRY
1013	0	1	0	0	1	CDH15
1014	0	0	0	0	1	CDH16
10149	0	0	0	0	1	ADGRG2
10157	0	0	0	0	1	AASS
10160	0	0	0	0	1	FARP1
10186	0	0	0	0	1	LHFPL6
10202	1	0	0	0	1	DHRS2
10234	0	0	0	0	1	LRRC17
10253	0	0	1	0	1	SPRY2
1026	0	0	0	0	1	CDKN1A
1028	0	0	0	0	1	CDKN1C
1032	0	0	0	0	1	CDKN2D
10347	0	0	0	0	1	ABCA7
10371	0	0	0	0	1	SEMA3A
10439	0	0	0	0	1	OLFM1
1051	0	0	0	0	1	CEBPB

ENTREZ	p65 iso5	p65 iso5_Dex	Dex	GFP_Dex	p65 iso5_Dex_vs_p65 iso5	Symbol
10516	0	0	0	0	1	FBLN5
1052	0	0	0	0	1	CEBPD
10529	0	0	0	0	1	NEBL
10538	1	1	0	0	0	BATF
10561	0	1	1	0	1	IFI44
10578	0	0	0	0	1	GNLY
10610	0	0	0	0	1	ST6GALNAC2
10620	1	0	0	0	0	ARID3B
10644	0	0	0	0	1	IGF2BP2
107	0	1	0	1	1	ADCY1
10747	0	1	0	0	0	MASP2
10769	0	0	0	0	1	PLK2
107984640	0	0	0	0	1	LBHD2
10826	0	0	0	0	1	FAXDC2
10882	0	0	1	0	1	C1QL1
10891	0	0	0	0	1	PPARGC1A
10957	0	0	0	0	1	PNRC1
10964	1	0	0	0	1	IFI44L
10991	0	1	0	0	0	SLC38A3
11005	0	0	1	0	1	SPINK5
11009	0	0	0	0	1	IL24
11057	0	0	0	0	1	ABHD2
11067	0	0	0	0	1	DEPP1
11095	0	0	0	0	1	ADAMTS8
11138	0	0	0	0	1	TBC1D8
11151	0	0	0	0	1	CORO1A
11154	0	1	0	0	0	AP4S1
11170	0	0	0	0	1	FAM107A
11173	0	0	0	0	1	ADAMTS7
11213	0	0	0	0	1	IRAK3
1122	0	0	0	0	1	CHML
11238	0	0	0	0	1	CA5B

ENTREZ	p65 iso5	p65 iso5_Dex	Dex	GFP_Dex	p65 iso5_Dex_vs_p65 iso5	Symbol
112487	0	0	0	0	1	DTD2
11254	0	0	0	0	1	SLC6A14
11264	0	0	0	0	1	PXMP4
11274	1	0	0	0	0	USP18
112812	0	1	0	0	0	FDX2
112817	0	0	0	0	1	HOGA1
113622	0	0	0	0	1	ADPRHL1
114569	0	0	1	0	0	MAL2
114769	0	1	0	0	0	CARD16
114794	0	1	0	0	0	ELFN2
114804	0	0	0	0	1	RNF157
114805	0	0	1	0	0	GALNT13
114879	0	0	0	0	1	OSBPL5
114881	0	0	0	0	1	OSBPL7
114897	1	1	0	0	0	C1QTNF1
114898	0	0	0	0	1	C1QTNF2
114907	0	0	0	0	1	FBXO32
114987	0	0	0	0	1	WDR31
115572	0	0	0	0	1	TENT5B
115703	0	0	1	0	0	ARHGAP33
115704	0	0	0	0	1	EVI5L
115749	0	1	0	0	0	C12orf56
116154	0	0	0	0	1	PHACTR3
116372	0	0	0	0	1	LYPD1
1164	0	0	0	0	1	CKS2
116496	0	0	1	0	0	NIBAN1
117283	0	0	0	0	1	IP6K3
117583	0	0	1	0	0	PARD3B
118429	0	0	0	0	1	ANTXR2
1187	0	0	0	0	1	CLCNKA
121227	0	0	0	0	1	LRIG3
121512	0	0	0	0	1	FGD4

ENTREZ	p65 iso5	p65 iso5_Dex	Dex	GFP_Dex	p65 iso5_Dex_vs_p65 iso5	Symbol
121601	0	0	0	0	1	ANO4
122402	0	0	0	0	1	TDRD9
122622	0	0	0	0	1	ADSS1
122970	0	0	1	0	0	ACOT4
123	0	0	0	0	1	PLIN2
123096	0	0	1	0	0	SLC25A29
1233	0	0	0	0	1	CCR4
123591	1	0	0	0	0	TMEM266
1238	0	0	0	0	1	ACKR2
1244	0	0	0	0	1	ABCC2
124857	0	1	0	1	0	WFIKKN2
126695	1	0	0	0	0	KDF1
1268	0	0	0	0	1	CNR1
127002	0	0	1	0	0	ATXN7L2
127255	1	0	0	0	0	LRRIQ3
127294	0	0	0	0	1	MYOM3
1280	0	0	0	0	1	COL2A1
1281	0	0	1	0	0	COL3A1
128218	1	0	0	0	1	TMEM125
1296	1	0	0	0	1	COL8A2
129804	1	1	0	0	1	FBLN7
129807	0	0	0	0	1	NEU4
129880	0	1	0	0	0	BBS5
1299	0	0	1	0	0	COL9A3
130367	1	1	0	0	0	SGPP2
1306	0	0	1	0	0	COL15A1
130612	0	0	0	0	1	TMEM198
1307	0	0	0	0	1	COL16A1
131578	0	0	1	0	0	LRRC15
132321	0	0	0	0	1	C4orf33
133522	0	0	0	0	1	PPARGC1B
134265	0	0	0	0	1	AFAP1L1

ENTREZ	p65 iso5	p65 iso5_Dex	Dex	GFP_Dex	p65 iso5_Dex_vs_p65 iso5	Symbol
135398	0	0	1	0	0	C6orf141
1363	0	0	0	0	1	CPE
1365	0	1	0	0	0	CLDN3
1366	0	0	0	0	1	CLDN7
1368	0	0	0	0	1	CPM
137075	0	0	0	0	1	CLDN23
1382	0	0	1	0	0	CRABP2
1396	0	0	0	0	1	CRIP1
139818	0	0	1	0	1	DOCK11
140462	0	0	1	0	0	ASB9
140606	0	0	1	0	0	SELENOM
140831	0	0	0	0	1	ZSWIM3
140885	0	0	0	0	1	SIRPA
140893	1	0	0	0	0	RBBP8NL
141	0	0	0	0	1	ADPRH
1410	0	0	0	0	1	CRYAB
1435	1	0	0	0	0	CSF1
1438	1	0	0	0	1	CSF2RA
144100	0	0	0	0	1	PLEKHA7
144535	0	1	0	0	0	CFAP54
145748	0	0	0	0	1	LYSMD4
145864	0	0	1	1	0	HAPLN3
146198	0	0	0	0	1	ZFP90
146330	0	0	1	0	1	FBXL16
1464	0	0	1	0	0	CSPG4
146664	0	0	0	0	1	MGAT5B
147	0	0	0	0	1	ADRA1B
147015	0	0	0	0	1	DHRS13
147138	0	1	0	1	1	TMC8
147381	0	1	0	1	1	CBLN2
147710	0	0	0	0	1	IGSF23
147837	0	0	0	0	1	ZNF563

ENTREZ	p65 iso5	p65 iso5_Dex	Dex	GFP_Dex	p65 iso5_Dex_vs_p65 iso5	Symbol
147906	0	0	0	0	1	DACT3
147912	0	0	1	0	0	SIX5
148229	0	0	0	0	1	ATP8B3
149076	0	0	1	0	0	ZNF362
149461	1	1	0	0	0	CLDN19
150	0	0	0	0	1	ADRA2A
150696	0	1	0	0	0	PROM2
151056	0	0	0	0	1	PLB1
151176	0	0	0	0	1	ERFE
151242	0	1	0	0	1	PPP1R1C
151636	1	1	0	0	0	DTX3L
151742	0	0	1	0	0	PPM1L
153020	0	0	0	0	1	RASGEF1B
153562	0	0	0	0	1	MARVELD2
1543	0	0	0	0	1	CYP1A1
154790	0	1	0	0	0	CLEC2L
155185	0	0	0	0	1	AMZ1
155382	0	0	0	0	1	VPS37D
157638	0	0	0	0	1	LRATD2
1580	0	0	0	0	1	CYP4B1
158219	0	0	0	0	1	TTC39B
158763	0	0	0	0	1	ARHGAP36
159195	0	0	0	0	1	USP54
160428	0	0	0	0	1	ALDH1L2
161176	0	0	0	0	1	SYNE3
162282	0	0	0	0	1	ANKFN1
162394	1	0	0	0	0	SLFN5
162494	0	0	0	0	1	RHBDL3
163259	0	0	0	0	1	DENND2C
1634	0	0	0	0	1	DCN
1645	0	0	0	0	1	AKR1C1
168667	0	0	0	0	1	BMPER

ENTREZ	p65 iso5	p65 iso5_Dex	Dex	GFP_Dex	p65 iso5_Dex_vs_p65 iso5	Symbol
1687	0	0	0	0	1	GSDME
169436	1	0	0	0	0	STKLD1
169611	0	0	1	0	0	OLFML2A
169841	0	1	0	0	0	ZNF169
170302	0	1	0	1	0	ARX
1734	0	0	0	0	1	DIO2
1745	0	0	0	0	1	DLX1
1746	0	0	0	0	1	DLX2
1747	0	0	0	0	1	DLX3
1749	0	1	0	0	0	DLX5
1755	0	0	0	0	1	DMBT1
1794	0	0	1	0	0	DOCK2
1808	0	0	0	0	1	DPYSL2
1831	0	0	0	0	1	TSC22D3
1836	0	0	0	0	1	SLC26A2
1842	0	0	0	0	1	ECM2
1843	0	0	0	0	1	DUSP1
1844	0	0	0	0	1	DUSP2
1846	0	0	1	0	0	DUSP4
1847	0	0	0	0	1	DUSP5
1870	0	0	0	0	1	E2F2
1906	0	0	0	1	0	EDN1
1907	1	0	1	0	1	EDN2
1942	0	0	0	0	1	EFNA1
1958	0	0	0	0	1	EGR1
1969	0	0	1	0	0	EPHA2
198437	0	1	0	0	0	LKAAEAR1
199800	0	0	0	0	1	ADM5
2	0	0	1	0	0	A2M
200150	0	0	0	0	1	PLD5
200159	0	1	0	0	0	SPMIP3
2006	0	0	1	0	1	ELN

ENTREZ	p65 iso5	p65 iso5_Dex	Dex	GFP_Dex	p65 iso5_Dex_vs_p65 iso5	Symbol
201164	0	0	0	0	1	PLD6
2012	0	0	0	0	1	EMP1
201255	0	0	0	0	1	LRRC45
2013	0	0	0	0	1	EMP2
201780	0	1	0	0	0	SLC10A4
202	0	0	1	0	0	CRYBG1
2026	0	0	1	0	0	ENO2
2027	0	0	0	0	1	ENO3
203238	0	0	0	0	1	CCDC171
203260	0	0	0	0	1	CCDC107
2034	0	0	0	0	1	EPAS1
2039	0	0	1	0	0	DMTN
2040	0	0	0	0	1	STOM
2048	0	0	1	0	1	EPHB2
2053	0	0	0	0	1	EPHX2
2059	0	0	0	0	1	EPS8
2065	0	0	0	0	1	ERBB3
2069	0	0	1	0	0	EREG
2072	0	0	0	0	1	ERCC4
2081	0	0	0	0	1	ERN1
2086	0	1	0	0	0	ERV3-1
2115	0	0	1	0	0	ETV1
2118	0	0	1	0	0	ETV4
2119	0	0	1	0	0	ETV5
2139	1	1	0	1	1	EYA2
2149	0	0	0	0	1	F2R
2150	0	0	0	0	1	F2RL1
2151	0	0	0	1	0	F2RL2
2152	0	0	1	0	0	F3
2159	0	0	0	0	1	F10
218	0	0	1	0	0	ALDH3A1
2185	0	0	0	0	1	PTK2B

ENTREZ	p65 iso5	p65 iso5_Dex	Dex	GFP_Dex	p65 iso5_Dex_vs_p65 iso5	Symbol
219285	1	0	0	0	0	SAMD9L
219699	0	0	0	0	1	UNC5B
220	0	0	1	0	0	ALDH1A3
220213	0	0	0	0	1	OTUD1
220963	0	0	0	0	1	SLC16A9
221002	0	1	0	1	1	RASGEF1A
221188	0	0	0	0	1	ADGRG5
221481	0	0	0	0	1	ARMC12
221527	0	0	0	0	1	ZBTB12
222235	0	0	0	0	1	FBXL13
222663	1	0	0	0	0	SCUBE3
222950	0	0	0	0	1	NYAP1
222962	0	0	0	0	1	SLC29A4
2244	0	0	0	0	1	FGB
2257	0	0	0	0	1	FGF12
22795	0	0	0	0	1	NID2
22801	0	0	1	0	0	ITGA11
2281	0	1	0	0	0	FKBP1B
22822	0	0	1	0	1	PHLDA1
22885	0	0	0	0	1	ABLIM3
2289	0	0	0	0	1	FKBP5
22943	0	0	0	0	1	DKK1
22987	0	1	0	0	1	SV2C
2300	0	0	1	0	0	FOXL1
23034	0	0	0	0	1	SAMD4A
23037	1	0	0	0	0	PDZD2
23051	0	0	0	0	1	ZHX3
2307	0	1	0	0	0	FOXS1
2308	0	0	1	0	0	FOXO1
23086	0	0	0	0	1	EXPH5
23089	0	0	1	0	0	PEG10
23105	0	0	1	0	1	FSTL4

ENTREZ	p65 iso5	p65 iso5_Dex	Dex	GFP_Dex	p65 iso5_Dex_vs_p65 iso5	Symbol
23151	0	0	0	0	1	GRAMD4
23175	0	0	1	0	1	LPIN1
23208	0	0	0	0	1	SYT11
23224	0	0	1	0	1	SYNE2
23266	0	0	0	0	1	ADGRL2
23281	0	0	0	0	1	MTUS2
23284	0	0	0	0	1	ADGRL3
23303	0	0	0	0	1	KIF13B
23308	0	0	0	0	1	ICOSLG
23329	0	0	1	0	0	TBC1D30
2335	0	0	0	0	1	FN1
23363	0	0	0	0	1	OBSL1
23371	0	0	0	0	1	TNS2
23424	1	0	0	0	0	TDRD7
23428	0	0	0	0	1	SLC7A8
23452	1	0	0	0	0	ANGPTL2
23508	0	0	0	0	1	TTC9
2353	0	0	0	0	1	FOS
2354	0	0	0	0	1	FOSB
23541	0	0	0	0	1	SEC14L2
23551	0	0	0	0	1	RASD2
23586	1	1	1	0	1	RIGI
23596	0	0	0	0	1	OPN3
23623	0	0	0	0	1	RUSC1
23635	0	0	0	0	1	SSBP2
23650	0	0	0	0	1	TRIM29
23657	0	0	0	0	1	SLC7A11
23705	0	0	0	0	1	CADM1
23764	1	0	1	0	0	MAFF
23767	0	0	0	0	1	FLRT3
239	0	0	0	0	1	ALOX12
24	0	0	0	0	1	ABCA4

ENTREZ	p65 iso5	p65 iso5_Dex	Dex	GFP_Dex	p65 iso5_Dex_vs_p65 iso5	Symbol
241	0	0	0	0	1	ALOX5AP
250	0	0	0	0	1	ALPP
2520	0	1	0	0	0	GAST
2534	0	0	0	0	1	FYN
2537	1	1	1	1	0	IFI6
254268	0	0	0	0	1	AKNAD1
254427	0	0	0	0	1	PROSER2
254552	0	0	0	0	1	NUDT8
254827	0	0	0	0	1	NAALADL2
254887	0	0	0	0	1	ZDHHC23
255057	0	0	1	0	0	CBARP
255324	0	0	0	0	1	EPGN
255743	0	0	1	0	1	NPNT
255783	0	0	0	0	1	INAFM1
256691	0	0	0	0	1	MAMDC2
257000	0	0	0	0	1	TINCR
257019	0	0	1	0	1	FRMD3
257194	0	0	0	0	1	NEGR1
25822	0	0	0	0	1	DNAJB5
25837	0	0	0	0	1	RAB26
25840	0	0	0	0	1	TMT1A
25841	0	0	0	0	1	ABTB2
25862	0	0	0	0	1	USP49
25884	0	1	0	0	1	CHRDL2
2591	0	0	0	0	1	GALNT3
259173	0	0	0	0	1	ALS2CL
2593	0	0	0	0	1	GAMT
259307	0	0	0	0	1	IL4I1
26027	0	0	0	0	1	ACOT11
26112	0	1	0	0	0	CCDC69
2615	1	0	0	0	0	LRRC32
2619	0	0	0	0	1	GAS1

ENTREZ	p65 iso5	p65 iso5_Dex	Dex	GFP_Dex	p65 iso5_Dex_vs_p65 iso5	Symbol
26207	0	0	0	0	1	PITPNC1
2621	0	0	0	0	1	GAS6
26249	0	0	0	0	1	KLHL3
2627	0	0	0	0	1	GATA6
26287	0	0	0	0	1	ANKRD2
26298	0	0	0	0	1	EHF
26301	0	1	0	1	1	GBGT1
2633	1	0	0	0	0	GBP1
2634	1	1	0	0	0	GBP2
2635	1	1	0	0	0	GBP3
2646	0	1	0	0	0	GCKR
26468	0	0	0	0	1	LHX6
26508	0	0	0	0	1	HEYL
2651	0	0	0	0	1	GCNT2
26579	0	0	1	0	0	MYEOV
26585	0	0	0	0	1	GREM1
2687	0	0	0	0	1	GGT5
26873	0	0	0	0	1	OPLAH
2695	0	0	0	0	1	GIP
27006	0	1	0	0	0	FGF22
2706	0	0	0	0	1	GJB2
27063	0	0	0	0	1	ANKRD1
27065	0	0	0	0	1	NSG1
2707	0	0	1	0	1	GJB3
27086	0	0	1	0	1	FOXP1
27122	0	0	0	0	1	DKK3
27129	0	0	0	0	1	HSPB7
27133	0	0	1	0	0	KCNH5
27145	0	1	0	0	1	FILIP1
27151	0	0	0	0	1	CPAMD8
27202	0	0	0	0	1	C5AR2
27244	0	0	0	0	1	SESN1

ENTREZ	p65 iso5	p65 iso5_Dex	Dex	GFP_Dex	p65 iso5_Dex_vs_p65 iso5	Symbol
27284	0	0	0	0	1	SULT1B1
27345	0	0	0	0	1	KCNMB4
2752	0	0	0	0	1	GLUL
2766	0	0	0	0	1	GMPR
28231	0	0	0	0	1	SLCO4A1
2825	0	0	0	0	1	CMKLR2
283208	0	0	0	0	1	P4HA3
283316	0	1	0	0	1	CD163L1
2835	0	1	0	0	0	GPR12
283537	0	0	0	0	1	SLC46A3
283807	0	0	0	0	1	FBXL22
283933	0	0	0	0	1	ZNF843
283987	0	0	0	0	1	HID1
2840	1	0	0	0	1	GPR17
284076	1	0	0	0	1	TTLL6
284348	0	0	0	0	1	LYPD5
284443	0	1	0	0	0	ZNF493
285313	0	0	1	0	1	IGSF10
285315	0	1	0	0	0	C3orf33
286	0	0	0	0	1	ANK1
286343	0	0	0	0	1	LURAP1L
2875	0	0	0	0	1	GPT
2882	0	0	0	0	1	GPX7
28955	0	1	0	0	0	DEXI
28984	0	0	0	0	1	RGCC
28996	0	0	0	0	1	HIPK2
290	0	0	0	0	1	ANPEP
2901	0	0	0	0	1	GRIK5
29098	0	1	0	0	0	RANGRF
2919	0	1	0	0	1	CXCL1
2934	0	0	0	0	1	GSN
29767	0	0	0	0	1	TMOD2

ENTREZ	p65 iso5	p65 iso5_Dex	Dex	GFP_Dex	p65 iso5_Dex_vs_p65 iso5	Symbol
29903	0	0	0	0	1	CCDC106
29904	0	0	1	0	0	EEF2K
29907	0	0	0	0	1	SNX15
29949	0	0	0	0	1	IL19
30817	0	0	1	0	0	ADGRE2
30818	0	0	0	0	1	KCNIP3
30819	1	0	0	0	0	KCNIP2
3119	0	1	0	1	1	HLA-DQB1
3134	1	1	0	0	0	HLA-F
314	0	1	0	0	0	AOC2
316	0	0	0	0	1	AOX1
3162	0	0	1	1	1	HMOX1
3175	0	1	0	0	1	ONECUT1
3224	0	1	0	0	0	HOXC8
323	0	0	0	0	1	APBB2
3242	0	0	1	0	0	HPD
3248	0	0	1	0	1	HPGD
33	0	0	0	0	1	ACADL
330	0	0	0	0	1	BIRC3
333	0	0	1	0	0	APLP1
3338	0	0	0	0	1	DNAJC4
3373	0	0	0	0	1	HYAL1
3383	1	1	0	1	1	ICAM1
338322	0	0	0	0	1	NLRP10
338645	0	1	0	0	0	LUZP2
338761	0	0	0	0	1	C1QL4
339453	0	0	0	0	1	TMEM240
340024	1	0	0	0	0	SLC6A19
340273	0	0	0	0	1	ABCB5
340371	0	0	0	0	1	NRBP2
341350	1	0	0	0	1	OVCH1
3418	0	0	1	0	0	IDH2

ENTREZ	p65 iso5	p65 iso5_Dex	Dex	GFP_Dex	p65 iso5_Dex_vs_p65 iso5	Symbol
342346	0	0	0	0	1	C16orf96
342667	0	0	0	0	1	STAC2
3428	1	0	0	0	0	IFI16
3429	1	1	1	0	0	IFI27
3430	1	0	0	0	0	IFI35
3431	1	0	0	0	0	SP110
3433	1	1	0	0	0	IFIT2
3434	1	0	0	0	0	IFIT1
3437	1	1	0	0	0	IFIT3
344558	0	0	0	0	1	SH3RF3
344658	0	1	0	1	1	SAMD7
345651	0	0	0	0	1	ACTBL2
347051	0	1	0	0	1	SLC10A5
347273	0	1	0	0	0	CAVIN4
347454	1	0	0	1	0	SOWAHD
347733	0	0	0	0	1	TUBB2B
3479	0	0	0	0	1	IGF1
347902	0	0	1	1	1	AMIGO2
3484	0	0	0	0	1	IGFBP1
3489	1	0	0	0	0	IGFBP6
353355	0	1	0	0	0	ZNF233
3553	1	1	0	0	0	IL1B
3554	0	0	0	0	1	IL1R1
357	1	0	0	0	1	SHROOM2
3570	0	0	0	0	1	IL6R
3576	1	0	1	1	0	CXCL8
3589	0	0	1	1	1	IL11
3601	0	0	1	0	1	IL15RA
3604	0	1	0	0	0	TNFRSF9
3613	0	0	0	0	1	IMPA2
3623	1	0	0	0	1	INHA
3627	0	1	0	0	0	CXCL10

ENTREZ	p65 iso5	p65 iso5_Dex	Dex	GFP_Dex	p65 iso5_Dex_vs_p65 iso5	Symbol
3656	1	0	1	0	0	IRAK2
3659	0	0	1	0	0	IRF1
3665	1	0	0	0	0	IRF7
3672	0	0	0	0	1	ITGA1
3676	0	0	0	0	1	ITGA4
3678	0	0	0	0	1	ITGA5
3679	0	0	0	1	0	ITGA7
3691	0	0	0	0	1	ITGB4
3708	0	0	0	0	1	ITPR1
3710	0	0	0	0	1	ITPR3
3730	0	0	1	0	0	ANOS1
374	0	0	0	0	1	AREG
374393	0	0	0	0	1	FAM111B
374918	1	0	0	0	0	IGFL1
3752	0	0	0	0	1	KCND3
375704	0	0	0	0	1	ENHO
3759	0	0	0	0	1	KCNJ2
376267	0	0	1	0	0	RAB15
3768	0	0	1	0	0	KCNJ12
3770	0	1	0	0	0	KCNJ14
3784	0	0	0	0	1	KCNQ1
3822	0	0	0	1	0	KLRC2
3848	1	1	0	0	0	KRT1
3851	0	0	0	0	1	KRT4
3853	0	0	1	0	1	KRT6A
3860	0	0	1	0	1	KRT13
3861	0	0	1	0	0	KRT14
3868	0	0	1	1	1	KRT16
387509	0	0	0	0	1	GPR153
387700	0	0	0	0	1	SLC16A12
387718	0	0	0	0	1	TEX36
387758	1	0	0	0	1	FIBIN

ENTREZ	p65 iso5	p65 iso5_Dex	Dex	GFP_Dex	p65 iso5_Dex_vs_p65 iso5	Symbol
3880	0	0	1	0	0	KRT19
388121	0	0	0	0	1	TNFAIP8L3
388341	0	0	0	0	1	LRRC75A
3885	0	0	0	0	1	KRT34
388610	0	0	0	0	1	TRNP1
3887	0	0	0	0	1	KRT81
389136	0	0	0	0	1	VGLL3
3892	0	0	0	0	1	KRT86
389692	0	0	0	0	1	MAFA
389792	0	0	0	0	1	IER5L
389941	0	1	0	0	0	C1QL3
3908	0	1	0	0	0	LAMA2
3909	1	0	0	0	0	LAMA3
3913	0	0	1	0	0	LAMB2
3932	0	0	0	0	1	LCK
3934	1	0	0	0	1	LCN2
3936	0	1	0	0	1	LCP1
3965	1	0	0	0	0	LGALS9
3976	0	0	1	0	0	LIF
399474	0	0	0	0	1	TMEM200B
399818	0	0	0	0	1	EEF1AKMT2
400499	0	0	0	0	1	LOC400499
400745	0	0	1	0	0	SH2D5
401024	0	0	0	0	1	FSIP2
4015	0	0	0	0	1	LOX
402573	0	1	0	1	1	SPACDR
402682	0	1	0	0	0	UFSP1
404217	0	0	0	0	1	CTXN1
404550	0	0	0	0	1	C16orf74
404734	0	0	0	1	1	ANKHD1-EIF4EBP3
4059	0	0	1	0	0	BCAM
4064	0	0	0	1	0	CD180

ENTREZ	p65 iso5	p65 iso5_Dex	Dex	GFP_Dex	p65 iso5_Dex_vs_p65 iso5	Symbol
4066	0	1	0	1	1	LYL1
4072	0	1	0	0	0	EPCAM
4082	0	0	1	0	0	MARCKS
41	0	0	0	0	1	ASIC1
4128	0	0	0	0	1	MAOA
4130	0	0	1	0	0	MAP1A
4133	0	0	0	0	1	MAP2
4137	1	0	0	0	0	MAPT
4147	0	0	1	0	0	MATN2
4208	0	1	0	0	0	MEF2C
4222	0	0	0	0	1	MEOX1
4237	0	0	1	0	0	MFAP2
4240	0	0	0	0	1	MFGE8
4303	0	0	1	0	0	FOXO4
4354	0	0	0	0	1	MPP1
4355	0	0	0	0	1	MPP2
440	0	1	0	0	0	ASNS
440335	1	0	0	0	0	SMIM22
440345	0	0	0	0	1	NPIPB4
441168	0	1	0	0	0	CALHM6
442117	0	0	0	0	1	GALNTL6
442590	1	0	0	0	1	SPDYE5
4493	0	0	0	0	1	MT1E
4495	0	0	0	0	1	MT1G
4501	0	0	0	0	1	MT1X
4502	0	0	0	0	1	MT2A
4582	1	0	0	0	1	MUC1
4599	1	0	0	0	0	MX1
4600	0	1	0	0	1	MX2
4621	0	0	0	0	1	MYH3
4633	0	0	0	0	1	MYL2
4640	0	0	0	0	1	MYO1A

ENTREZ	p65 iso5	p65 iso5_Dex	Dex	GFP_Dex	p65 iso5_Dex_vs_p65 iso5	Symbol
4645	0	0	1	0	0	MYO5B
4647	0	0	0	0	1	MYO7A
477	0	0	0	0	1	ATP1A2
4773	0	0	0	0	1	NFATC2
4808	0	1	0	0	0	NHLH2
4810	0	0	0	0	1	NHS
4811	0	0	1	0	1	NID1
482	0	0	0	0	1	ATP1B2
4837	0	0	0	0	1	NNMT
4854	0	0	0	0	1	NOTCH3
4856	0	0	0	0	1	CCN3
489	0	0	0	0	1	ATP2A3
4897	0	0	0	0	1	NRCAM
4899	0	0	0	0	1	NRF1
4900	0	0	1	0	0	NRGN
4907	0	0	1	0	0	NT5E
4938	1	1	0	0	0	OAS1
4939	0	0	1	0	0	OAS2
4940	1	1	0	0	0	OAS3
4973	1	1	0	0	1	OLR1
4982	0	0	0	0	1	TNFRSF11B
50515	0	0	1	0	0	CHST11
5054	0	0	1	1	1	SERPINE1
50617	0	0	0	0	1	ATP6V0A4
5064	0	0	1	0	0	PALM
50651	0	0	0	0	1	SLC45A1
5080	0	0	1	0	0	PAX6
50848	0	0	0	0	1	F11R
5101	0	0	0	0	1	PCDH9
51110	0	0	0	0	1	LACTB2
51127	1	0	0	0	0	TRIM17
51129	0	0	1	0	1	ANGPTL4

ENTREZ	p65 iso5	p65 iso5_Dex	Dex	GFP_Dex	p65 iso5_Dex_vs_p65 iso5	Symbol
51147	0	0	0	0	1	ING4
51161	0	0	0	0	1	C3orf18
51191	1	0	0	0	0	HERC5
51196	0	0	0	0	1	PLCE1
5129	0	0	1	0	0	CDK18
51296	1	1	0	0	0	SLC15A3
51309	0	0	1	0	0	ARMCX1
51330	0	0	1	0	0	TNFRSF12A
51334	0	0	0	0	1	PRR16
5137	0	1	0	0	0	PDE1C
5145	0	0	0	0	1	PDE6A
51463	0	0	0	0	1	GPR89B
51513	1	0	0	0	1	ETV7
5155	0	0	0	1	0	PDGFB
51560	0	0	0	0	1	RAB6B
51561	0	0	0	1	0	IL23A
5159	0	0	0	0	1	PDGFRB
5166	0	0	1	0	1	PDK4
5167	1	0	1	0	1	ENPP1
51673	1	0	0	0	1	TPPP3
5187	0	0	0	0	1	PER1
5205	0	0	1	0	0	ATP8B1
5210	0	0	0	0	1	PFKFB4
5266	1	1	1	0	0	PI3
5270	0	0	1	0	1	SERPINE2
5272	0	0	0	0	1	SERPINB9
5295	0	0	0	0	1	PIK3R1
5296	1	0	0	0	1	PIK3R2
5318	0	0	0	0	1	PKP2
5324	0	0	0	0	1	PLAG1
5328	0	0	0	0	1	PLAU
5337	0	0	1	0	0	PLD1

ENTREZ	p65 iso5	p65 iso5_Dex	Dex	GFP_Dex	p65 iso5_Dex_vs_p65 iso5	Symbol
5359	1	0	0	0	0	PLSCR1
5362	1	0	0	0	1	PLXNA2
5365	0	0	0	0	1	PLXNB3
5371	1	0	0	0	0	PML
5372	0	0	0	0	1	PMM1
53833	0	0	0	0	1	IL20RB
53836	0	0	0	0	1	GPR87
5409	0	0	0	0	1	PNMT
5414	0	1	0	0	0	SEPTIN4
54206	0	0	0	0	1	ERRFI1
54436	0	0	0	0	1	SH3TC1
5445	0	0	0	0	1	PON2
54491	0	0	0	0	1	OTULINL
54535	0	0	0	0	1	CCHCR1
54541	0	0	0	0	1	DDIT4
54577	0	0	0	0	1	UGT1A7
54584	0	0	1	0	0	GNB1L
54625	1	1	0	0	0	PARP14
54809	1	0	0	0	0	SAMD9
54831	0	0	0	0	1	BEST2
54852	0	0	1	0	0	PAQR5
54858	0	0	0	0	1	PGPEP1
5493	0	0	0	0	1	PPL
54946	0	0	0	0	1	SLC41A3
55008	1	1	1	0	0	HERC6
55062	0	0	0	0	1	WIPI1
55084	0	1	0	0	0	SOBP
55089	1	0	0	0	1	SLC38A4
55112	0	0	0	0	1	DYNC2I1
55130	0	0	0	0	1	ODAD2
5521	0	0	1	0	0	PPP2R2B
55222	0	0	0	0	1	LRRC20

ENTREZ	p65 iso5	p65 iso5_Dex	Dex	GFP_Dex	p65 iso5_Dex_vs_p65 iso5	Symbol
55224	0	0	0	0	1	ETNK2
55273	0	0	0	0	1	TMEM100
55301	0	0	0	0	1	OLAH
55332	0	0	0	0	1	DRAM1
55337	1	0	0	0	0	SHFL
55351	0	0	0	0	1	STK32B
55356	0	0	0	0	1	SLC22A15
55367	0	0	0	0	1	PIDD1
554	0	0	0	0	1	AVPR2
55450	0	1	0	0	1	CAMK2N1
55521	0	0	0	0	1	TRIM36
55540	0	0	0	0	1	IL17RB
55561	0	0	1	0	1	CDC42BPG
55567	0	0	0	0	1	DNAH3
55601	1	1	0	1	1	DDX60
55603	0	0	1	0	0	TENT5A
55616	0	0	1	0	0	ASAP3
55620	0	0	0	1	0	STAP2
55640	0	0	0	0	1	FLVCR2
55650	0	0	0	0	1	PIGV
55723	0	0	0	0	1	ASF1B
55731	0	0	0	0	1	FAM222B
55733	0	0	1	0	0	HHAT
5579	0	0	1	0	0	PRKCB
55803	0	0	0	0	1	ADAP2
55857	0	0	0	0	1	KIZ
5587	0	0	0	0	1	PRKD1
55893	0	0	0	0	1	ZNF395
55908	1	0	0	1	1	ANGPTL8
5593	1	0	0	0	1	PRKG2
55930	0	0	1	0	0	MYO5C
5596	0	0	0	0	1	MAPK4

ENTREZ	p65 iso5	p65 iso5_Dex	Dex	GFP_Dex	p65 iso5_Dex_vs_p65 iso5	Symbol
55964	0	0	0	0	1	SEPTIN3
56033	0	0	1	0	0	BARX1
56109	0	0	0	0	1	PCDHGA6
56134	0	0	0	0	1	PCDHAC2
56137	0	1	0	0	0	PCDHA12
56241	0	0	0	0	1	SUSD2
5627	0	0	0	0	1	PROS1
56521	0	0	1	0	0	DNAJC12
56548	0	0	0	0	1	CHST7
56704	0	0	0	0	1	JPH1
5675	0	0	0	0	1	PSG6
56829	1	0	0	0	0	ZC3HAV1
56892	0	0	0	0	1	TCIM
56917	0	0	0	0	1	MEIS3
56928	0	0	0	0	1	SPPL2B
56937	0	0	1	0	0	PMEPA1
56952	0	0	0	0	1	PRTFDC1
5698	1	1	0	0	1	PSMB9
57016	0	0	0	0	1	AKR1B10
57096	0	1	0	0	0	RPGRIP1
57121	0	0	1	1	0	LPAR5
57139	0	0	0	0	1	RGL3
57149	0	0	0	0	1	LYRM1
57156	0	0	0	0	1	TMEM63C
57169	1	0	0	0	0	ZNFX1
57211	0	0	0	0	1	ADGRG6
57214	1	0	0	0	0	CEMIP
57216	0	1	0	1	1	VANGL2
57221	0	0	1	0	0	ARFGEF3
57224	0	0	0	0	1	NHSL1
5732	0	0	0	0	1	PTGER2
57326	0	0	1	0	0	PBXIP1

ENTREZ	p65 iso5	p65 iso5_Dex	Dex	GFP_Dex	p65 iso5_Dex_vs_p65 iso5	Symbol
57333	0	0	1	0	0	RCN3
57335	0	0	0	0	1	ZNF286A
5737	1	0	0	0	1	PTGFR
57402	0	0	1	0	0	S100A14
5744	0	0	1	0	1	PTHLH
57464	0	0	0	0	1	STRIP2
57474	0	1	0	0	0	ZNF490
57476	0	0	0	0	1	GRAMD1B
57480	0	0	0	0	1	PLEKHG1
57507	1	0	0	0	0	ZNF608
57523	0	0	0	0	1	NYNRIN
57528	0	1	0	0	0	KCTD16
57535	0	0	0	0	1	ELAPOR1
57554	0	1	0	0	0	LRRC7
57559	0	0	1	0	0	STAMBPL1
57568	0	0	0	0	1	SIPA1L2
57576	1	0	0	0	0	KIF17
57595	0	0	0	0	1	PDZD4
57596	0	0	0	0	1	BEGAIN
57608	0	0	0	0	1	JCAD
57624	0	1	0	0	1	NYAP2
57626	0	1	0	0	0	KLHL1
57664	1	1	0	0	0	PLEKHA4
57669	0	0	0	0	1	EPB41L5
57822	0	1	0	1	1	GRHL3
5791	0	0	1	0	0	PTPRE
582	0	1	0	0	0	BBS1
58476	0	0	0	0	1	TP53INP2
58500	0	0	0	0	1	ZNF250
58516	0	0	0	0	1	SINHCAF
5858	0	1	0	0	0	PZP
5873	0	0	0	0	1	RAB27A

ENTREZ	p65 iso5	p65 iso5_Dex	Dex	GFP_Dex	p65 iso5_Dex_vs_p65 iso5	Symbol
59	0	0	1	0	0	ACTA2
5920	0	1	0	1	1	PLAAT4
59344	0	0	0	0	1	ALOXE3
59352	0	0	0	0	1	LGR6
595	0	0	1	0	0	CCND1
5970	0	0	1	1	1	RELA
5971	0	0	1	0	1	RELB
5973	0	0	0	0	1	RENBP
598	0	0	0	0	1	BCL2L1
5990	0	0	0	0	1	RFX2
5997	0	0	1	0	1	RGS2
6004	0	0	0	0	1	RGS16
60680	0	0	0	0	1	CELF5
6094	0	0	0	0	1	ROM1
6097	0	0	0	0	1	RORC
619373	1	1	0	1	0	MBOAT4
622	0	0	0	0	1	BDH1
6236	1	0	0	0	1	RRAD
6252	0	0	0	0	1	RTN1
6258	0	0	0	0	1	RXRG
6261	0	0	0	0	1	RYR1
6272	0	0	1	0	0	SORT1
6286	0	0	0	0	1	S100P
6288	0	0	0	0	1	SAA1
6303	0	0	0	0	1	SAT1
63035	0	0	1	0	0	BCORL1
6310	0	0	0	0	1	ATXN1
6337	0	0	0	0	1	SCNN1A
6345	0	0	0	0	1	SRL
6352	0	0	1	0	0	CCL5
6364	1	0	0	0	0	CCL20
638	1	0	0	0	1	BIK

ENTREZ	p65 iso5	p65 iso5_Dex	Dex	GFP_Dex	p65 iso5_Dex_vs_p65 iso5	Symbol
6382	0	0	0	0	1	SDC1
6385	1	0	1	0	0	SDC4
63924	0	0	0	0	1	CIDEC
64005	1	0	0	0	1	MYO1G
64061	0	0	0	0	1	TSPYL2
64093	0	0	0	0	1	SMOC1
64097	0	0	0	0	1	EPB41L4A
64115	0	0	0	0	1	VSIR
64135	1	0	1	0	0	IFIH1
64137	0	0	0	0	1	ABCG4
64220	0	0	0	0	1	STRA6
64288	0	0	0	0	1	ZSCAN31
643338	0	1	0	0	1	C15orf62
64377	0	0	0	0	1	CHST8
64386	0	0	0	0	1	MMP25
644844	0	1	0	0	0	PHGR1
6453	0	0	0	0	1	ITSN1
645426	0	1	0	0	0	TMEM191C
64699	0	0	1	0	0	TMPRSS3
647024	0	0	0	0	1	C6orf132
6474	0	0	0	0	1	SHOX2
64753	0	0	0	0	1	CCDC136
64761	1	1	0	0	0	PARP12
64762	0	0	0	0	1	GAREM1
64780	0	0	0	0	1	MICAL1
6482	0	0	0	0	1	ST3GAL1
64838	0	1	0	0	0	FNDC4
64840	0	0	1	0	1	PORCN
64900	0	0	0	0	1	LPIN3
6493	0	0	0	0	1	SIM2
6494	0	0	0	0	1	SIPA1
65055	0	0	0	0	1	REEP1

ENTREZ	p65 iso5	p65 iso5_Dex	Dex	GFP_Dex	p65 iso5_Dex_vs_p65 iso5	Symbol
6518	0	0	0	0	1	SLC2A5
652	0	0	1	0	0	BMP4
65249	0	0	0	0	1	ZSWIM4
6526	0	0	0	0	1	SLC5A3
6531	0	0	0	0	1	SLC6A3
653145	0	0	0	0	1	ANXA8
6532	0	1	0	0	0	SLC6A4
6533	0	0	0	0	1	SLC6A6
653820	0	0	0	0	1	FAM72B
6539	0	1	0	0	0	SLC6A12
6542	0	0	0	0	1	SLC7A2
656	0	0	0	0	1	BMP8B
6560	0	0	0	0	1	SLC12A4
6578	0	0	0	0	1	SLCO2A1
6584	0	0	0	0	1	SLC22A5
6591	0	0	0	0	1	SNAI2
66004	0	0	0	0	1	LYNX1
66035	1	0	0	0	0	SLC2A11
6662	0	0	0	0	1	SOX9
6714	0	0	1	0	0	SRC
6737	1	0	0	0	0	TRIM21
6772	1	0	0	0	0	STAT1
6804	0	0	1	0	0	STX1A
6833	1	0	0	0	1	ABCC8
6865	1	1	0	1	0	TACR2
687	0	0	0	0	1	KLF9
6876	0	0	0	1	0	TAGLN
6877	0	0	0	0	1	TAF5
6890	1	0	0	0	0	TAP1
6905	0	1	0	0	0	TBCE
6916	0	0	0	0	1	TBXAS1
6941	0	0	0	0	1	TCF19

ENTREZ	p65 iso5	p65 iso5_Dex	Dex	GFP_Dex	p65 iso5_Dex_vs_p65 iso5	Symbol
70	0	0	1	0	1	ACTC1
7020	0	0	0	0	1	TFAP2A
7042	0	0	0	0	1	TGFB2
7047	0	0	0	0	1	TGM4
7051	0	0	0	0	1	TGM1
7052	0	0	1	0	1	TGM2
7056	0	0	0	0	1	THBD
7068	0	0	1	0	0	THRB
7079	0	0	0	0	1	TIMP4
7097	0	0	0	0	1	TLR2
7099	0	0	1	0	0	TLR4
7103	0	0	0	1	1	TSPAN8
7122	0	0	0	1	0	CLDN5
7128	1	1	0	0	0	TNFAIP3
7133	1	0	0	0	0	TNFRSF1B
7134	0	0	1	0	0	TNNC1
7139	0	0	0	0	1	TNNT2
7145	0	0	0	0	1	TNS1
715	1	1	0	0	0	C1R
716	1	1	0	0	0	C1S
7161	0	0	0	0	1	TP73
718	1	1	0	0	1	C3
72	0	0	1	0	0	ACTG2
727	0	1	0	0	0	C5
727910	0	0	1	0	1	TLCD2
728	0	0	0	0	1	C5AR1
729238	1	0	0	1	1	SFTPA2
729873	0	1	0	0	0	TBC1D3
729920	0	0	0	0	1	CRPPA
7347	0	0	1	0	0	UCHL3
7351	0	0	1	0	0	UCP2
7424	0	0	1	0	0	VEGFC

ENTREZ	p65 iso5	p65 iso5_Dex	Dex	GFP_Dex	p65 iso5_Dex_vs_p65 iso5	Symbol
7436	0	0	1	0	0	VLDLR
7450	0	0	0	0	1	VWF
7477	0	0	0	0	1	WNT7B
7481	1	0	0	0	1	WNT11
7516	0	0	0	0	1	XRCC2
7538	0	0	0	0	1	ZFP36
7552	1	0	0	0	1	ZNF711
7678	0	0	0	0	1	ZNF124
7704	0	0	0	0	1	ZBTB16
7710	0	1	0	0	0	ZNF154
774	1	0	0	0	0	CACNA1B
7803	1	0	0	0	1	PTP4A1
7832	0	0	1	0	1	BTG2
7846	0	0	1	0	0	TUBA1A
78990	0	0	0	0	1	OTUB2
78991	0	0	0	0	1	PCYOX1L
79008	0	1	0	1	0	SLX1B
79056	0	0	0	0	1	PRRG4
79078	0	0	0	0	1	C1orf50
79094	0	0	0	0	1	CHAC1
79132	1	0	1	0	0	DHX58
79154	0	0	0	0	1	DHRS11
79173	0	0	0	0	1	BRME1
79652	1	1	0	1	1	TMEM204
79689	0	0	1	0	1	STEAP4
79729	0	0	0	0	1	SH3D21
79745	0	0	0	0	1	CLIP4
79751	0	0	0	0	1	SLC25A22
79762	0	0	0	0	1	C1orf115
79772	0	0	0	0	1	MCTP1
79774	0	0	0	0	1	GRTP1
79789	1	0	0	0	0	CLMN

ENTREZ	p65 iso5	p65 iso5_Dex	Dex	GFP_Dex	p65 iso5_Dex_vs_p65 iso5	Symbol
79846	0	0	0	0	1	CFAP69
79897	0	0	0	0	1	RPP21
79931	0	0	0	1	0	TNIP3
79937	0	0	0	0	1	CNTNAP3
79961	0	0	0	0	1	DENND2D
79987	0	0	0	0	1	SVEP1
8000	0	0	0	0	1	PSCA
80000	0	0	0	0	1	GREB1L
80019	0	0	0	0	1	UBTD1
80022	0	0	1	0	1	MYO15B
80031	0	0	0	0	1	SEMA6D
8013	0	0	0	0	1	NR4A3
80223	0	0	0	0	1	RAB11FIP1
80237	0	1	0	0	1	ELL3
80264	0	0	0	0	1	ZNF430
80323	0	0	0	0	1	CCDC68
80328	0	0	0	0	1	ULBP2
8061	0	0	1	0	1	FOSL1
80709	0	0	0	0	1	AKNA
80726	0	0	0	0	1	IQCN
80741	0	1	0	0	0	LY6G5C
8076	0	0	0	0	1	MFAP5
80820	0	0	0	0	1	EEPD1
80830	1	1	0	0	0	APOL6
80833	0	1	0	0	0	APOL3
814	0	0	0	0	1	CAMK4
81491	0	0	0	0	1	GPR63
81563	0	0	0	0	1	C1orf21
81607	1	0	0	0	1	NECTIN4
81618	0	0	0	0	1	ITM2C
81928	0	0	0	0	1	CABLES2
8193	0	0	0	0	1	DPF1

ENTREZ	p65 iso5	p65 iso5_Dex	Dex	GFP_Dex	p65 iso5_Dex_vs_p65 iso5	Symbol
8291	0	1	0	0	1	DYSF
8332	0	0	0	0	1	H2AC16
834	1	0	0	0	1	CASP1
8340	0	0	0	0	1	H2BC13
83445	0	0	0	0	1	GSG1
83473	0	0	0	0	1	KATNAL2
83483	0	1	0	0	1	PLVAP
83547	0	0	0	0	1	RILP
83604	0	0	1	0	0	TMEM47
83643	0	0	0	0	1	CCDC3
83666	1	1	0	0	0	PARP9
8367	0	1	0	1	1	H4C5
83715	0	0	0	0	1	ESPN
83851	0	1	0	0	0	SYT16
83857	0	0	0	0	1	TMTC1
83937	0	0	0	0	1	RASSF4
84000	1	1	0	0	0	TMPRSS13
84002	0	0	0	0	1	B3GNT5
84059	0	1	0	0	0	ADGRV1
84070	1	0	0	0	0	FAM186B
84152	0	1	0	0	1	PPP1R1B
84166	0	0	0	0	1	NLRC5
84171	0	0	1	0	0	LOXL4
84286	0	0	0	0	1	TMEM175
84306	0	0	1	0	0	PDCD2L
84314	0	0	0	0	1	TMEM107
8436	0	0	0	0	1	CAVIN2
84436	0	1	0	0	0	ZNF528
84443	0	0	0	0	1	FRMPD3
84446	1	0	0	0	1	BRSK1
84448	0	0	0	0	1	ABLIM2
84630	0	0	0	0	1	TTBK1

ENTREZ	p65 iso5	p65 iso5_Dex	Dex	GFP_Dex	p65 iso5_Dex_vs_p65 iso5	Symbol
84632	0	1	0	0	0	AFAP1L2
84674	0	0	0	0	1	CARD6
8470	0	0	0	0	1	SORBS2
84707	0	0	0	0	1	BEX2
8482	0	0	1	0	0	SEMA7A
8483	1	0	0	0	1	CILP
84842	0	0	0	0	1	HPDL
84875	1	1	0	0	0	PARP10
84913	0	0	0	0	1	ATOH8
84940	0	0	0	0	1	CORO6
84951	0	0	0	0	1	TNS4
84959	0	0	1	0	0	UBASH3B
84978	0	0	0	0	1	FRMD5
84985	0	0	1	0	0	FAM83A
84986	0	0	0	0	1	ARHGAP19
85004	0	0	0	0	1	RERG
8507	0	0	0	0	1	ENC1
8519	1	1	0	0	0	IFITM1
8522	0	0	1	0	1	GAS7
8541	0	0	0	0	1	PPFIA3
8542	1	1	0	0	0	APOL1
8543	0	0	0	0	1	LMO4
85441	1	1	0	0	0	HELZ2
85449	0	0	0	0	1	KIAA1755
85455	0	0	0	0	1	DISP2
85458	0	0	0	0	1	DIXDC1
8549	0	0	0	0	1	LGR5
8553	0	0	1	0	0	BHLHE40
8601	0	0	1	0	0	RGS20
8613	0	0	1	0	1	PLPP3
8614	0	0	1	0	0	STC2
8638	1	1	1	0	1	OASL

ENTREZ	p65 iso5	p65 iso5_Dex	Dex	GFP_Dex	p65 iso5_Dex_vs_p65 iso5	Symbol
8645	0	0	0	0	1	KCNK5
8651	0	0	0	0	1	SOCS1
8685	0	0	0	0	1	MARCO
8693	1	0	0	0	0	GALNT4
8701	0	0	0	0	1	DNAH11
8710	0	0	0	0	1	SERPINB7
8728	0	0	0	1	0	ADAM19
8736	0	0	0	0	1	MYOM1
8739	0	0	0	0	1	HRK
8740	1	0	0	0	1	TNFSF14
8743	0	1	0	1	0	TNFSF10
8786	0	1	0	0	0	RGS11
8796	0	0	0	0	1	SCEL
8858	0	0	0	1	1	PROZ
8863	0	0	1	0	0	PER3
8870	0	0	1	0	0	IER3
8878	0	0	0	0	1	SQSTM1
8900	0	0	0	0	1	CCNA1
8913	0	0	0	0	1	CACNA1G
8974	0	0	0	0	1	P4HA2
898	0	0	0	0	1	CCNE1
8991	0	0	1	0	0	SELENBP1
8993	0	1	0	1	0	PGLYRP1
89932	0	0	1	0	0	PAPLN
89958	0	0	0	0	1	SAPCD2
9002	0	0	0	0	1	F2RL3
90113	1	0	0	0	0	VWA5B2
9028	0	0	0	0	1	RHBDL1
90293	0	0	0	0	1	KLHL13
90313	0	0	0	0	1	TP53I13
90427	0	0	1	0	0	BMF
9051	0	0	0	0	1	PSTPIP1

ENTREZ	p65 iso5	p65 iso5_Dex	Dex	GFP_Dex	p65 iso5_Dex_vs_p65 iso5	Symbol
9056	0	0	0	0	1	SLC7A7
9064	0	0	0	0	1	MAP3K6
9076	0	0	1	0	0	CLDN1
90853	0	0	1	0	1	SPOCD1
9099	0	0	1	0	1	USP2
90990	0	0	0	0	1	KIFC2
91050	0	1	0	0	0	CCDC149
91147	0	1	0	0	0	TMEM67
91156	0	0	0	0	1	IGFN1
9124	0	0	0	0	1	PDLIM1
9134	0	0	0	0	1	CCNE2
91461	0	0	0	0	1	PKDCC
91543	1	0	1	0	0	RSAD2
91662	1	0	0	0	1	NLRP12
9170	0	0	0	0	1	LPAR2
9182	0	0	0	0	1	RASSF9
9185	0	0	1	0	0	REPS2
9201	0	0	0	0	1	DCLK1
9232	0	0	0	0	1	PTTG1
92370	0	0	0	0	1	PXYLP1
9245	1	0	0	0	1	GCNT3
9246	1	0	0	0	0	UBE2L6
9249	0	0	0	0	1	DHRS3
92558	0	0	0	0	1	BICDL1
92714	0	0	0	0	1	ARRDC1
9308	1	0	0	0	0	CD83
9315	0	0	0	0	1	NREP
93233	1	0	0	1	1	ODAD1
9340	0	0	0	0	1	GLP2R
9348	0	1	0	0	0	NDST3
9362	0	0	0	0	1	CPNE6
9363	1	0	0	0	1	RAB33A

ENTREZ	p65 iso5	p65 iso5_Dex	Dex	GFP_Dex	p65 iso5_Dex_vs_p65 iso5	Symbol
93649	0	0	0	0	1	MYOCD
9399	0	0	0	0	1	STOML1
94234	0	0	0	0	1	FOXQ1
94240	0	0	0	0	1	EPSTI1
9495	0	0	0	0	1	AKAP5
9496	1	0	0	0	1	TBX4
9500	1	0	0	0	0	MAGED1
9534	0	0	0	0	1	ZNF254
9536	0	0	1	0	0	PTGES
9537	0	0	0	0	1	TP53I11
9540	0	0	0	0	1	TP53I3
9590	0	0	0	0	1	AKAP12
9603	0	0	1	0	1	NFE2L3
9636	1	0	1	0	0	ISG15
9649	1	0	0	0	1	RALGPS1
9659	0	0	0	0	1	PDE4DIP
9672	0	0	0	0	1	SDC3
9674	0	0	0	0	1	KIAA0040
9681	0	0	0	0	1	DEPDC5
9750	0	0	0	0	1	RIPOR2
9762	0	0	0	0	1	LZTS3
9783	0	1	0	0	0	RIMS3
9788	1	0	0	0	0	MTSS1
9806	0	0	0	0	1	SPOCK2
9823	0	0	0	0	1	ARMCX2
9881	1	0	0	0	0	TRANK1
9900	0	0	1	0	0	SV2A
9935	0	0	0	0	1	MAFB
9955	0	0	0	0	1	HS3ST3A1
9975	0	0	0	0	1	NR1D2
